# Beyond the First Coordination Sphere—Manipulating the Excited-State Landscape in Iron(II) Chromophores with Protons

**DOI:** 10.1021/jacs.4c00552

**Published:** 2024-07-11

**Authors:** Kamil Witas, Shruthi Santhosh Nair, Tamar Maisuradze, Linda Zedler, Heiner Schmidt, Pablo Garcia-Porta, Alexandra Stefanie Jessica Rein, Tim Bolter, Sven Rau, Stephan Kupfer, Benjamin Dietzek-Ivanšić, Dieter U. Sorsche

**Affiliations:** † Institute for Inorganic Chemistry 1, Ulm University (UUlm), Albert-Einstein-Allee 11, Ulm 89081, Germany; ‡ Research Department Functional Interfaces, Leibniz Institute of Photonic Technology (Leibniz-IPHT), Albert-Einstein-Straße 9, Jena 07745, Germany; § Institute for Physical Chemistry, Friedrich-Schiller-Universität Jena (FSU Jena), Lessingstraße 4, Jena 07743, Germany

## Abstract

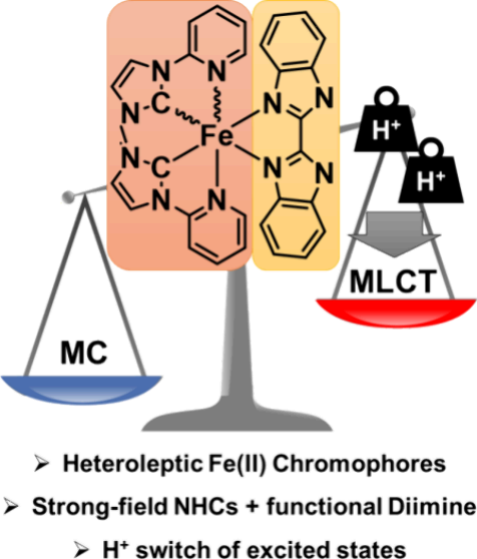

Molecular transition metal chromophores play a central role in light harvesting and energy conversion. Recently, earth-abundant transition-metal-based chromophores have begun to challenge the dominance of platinum group metal complexes in this area. However, the development of new chromophores with optimized photophysical properties is still limited by a lack of synthetic methods, especially with respect to heteroleptic complexes with functional ligands. Here, we demonstrate a facile and efficient method for the combination of strong-field carbenes with the functional 2,2′-bibenzimidazole ligand in a heteroleptic iron(II) chromophore complex. Our approach yields two isomers that differ predominantly in their excited-state lifetimes based on the symmetry of the ligand field. Deprotonation of both isomers leads to a significant red-shift of the metal-to-ligand charge transfer (MLCT) absorption and a shortening of excited-state lifetimes. Femtosecond transient absorption spectroscopy in combination with quantum chemical simulations and resonance Raman spectroscopy reveals the complex relationship between protonation and photophysical properties. Protonation is found to tip the balance between MLCT and metal-centered (MC) excited states in favor of the former. This study showcases the first example of fine-tuning of the excited-state landscape in an iron(II) chromophore through second-sphere manipulations and provides a new perspective to the challenge of excited-state optimizations in 3d transition metal chromophores.

## Introduction

**Figure 1:**
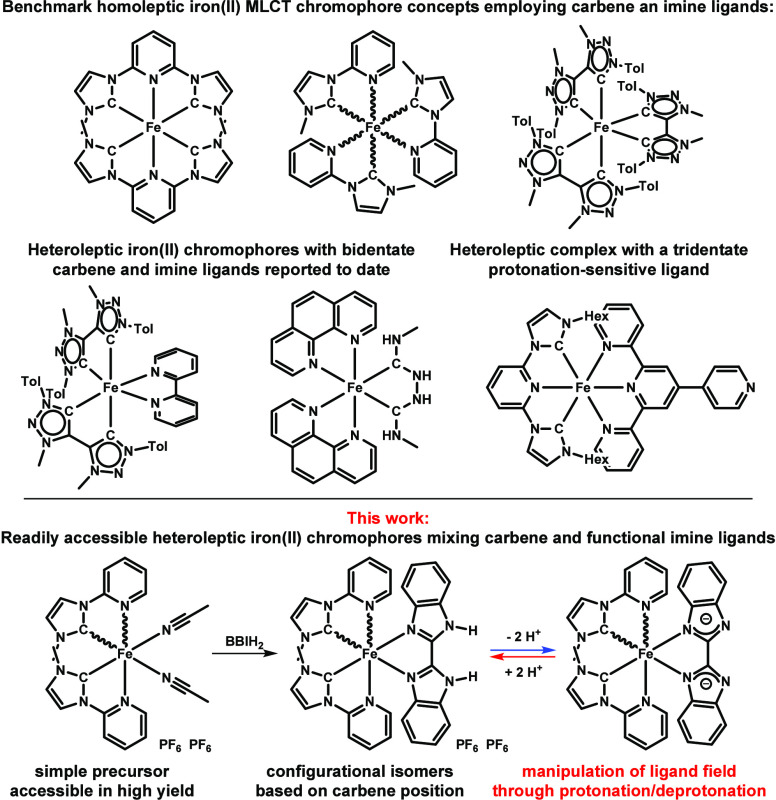
Benchmark iron(II) MLCT chromophores; top: homoleptic carbene complexes; center: iron(II) chromophores with heteroleptic ligand architectures comprising bidentate ligands (left, center) and pH-sensitive tridentate ligands (right); bottom: heteroleptic iron(II) chromophores with bidentate carbene/pyridine and a protonation-switchable 2,2′-bibenzimidazole ligand reported here; wavy bonds indicate isomers **C2** and **C1**.

Metal complex chromophores play a pivotal role in the development of light harvesting systems for solar-to-chemical energy conversion as a means toward a sustainable future energy landscape.^1^ Photoactive polypyridyl complexes of ruthenium and iridium have dominated this field for decades thanks to the relative ease of tailoring their structure and photophysical properties to integrate them into functional materials and supramolecular assemblies.^2−4^ The resulting plethora of systematically developed chromophores has enabled a deep understanding of structure–property relationships, which in turn led to advanced photochemical concepts such as photocatalyst repair^5,6^ and temporal decoupling of photoinduced processes from catalytic turnover.^7−9^ With regards to upscaling and sustainability, however, it is necessary to move away from rare and expensive platinum group metals to more sustainable 3d transition metals.^10−12^ Iron is particularly interesting in this regard, as it is the lighter homologue of ruthenium but significantly more abundant. However, iron being a 3d transition metal requires overall stronger ligands to stabilize charge transfer states, which are desirable with regards to photoredox processes.^13−15^ Introduction of N-heterocyclic carbenes (NHCs) has proven to be a powerful strategy to enable charge-separation on the picosecond-to-nanosecond scale, thus giving rise to applications in redox photochemistry.^16−18^ However, more advanced applications and optimization strategies are still hampered by a lack of universal synthetic procedures to access heteroleptic iron(II) chromophores. Benchmark chromophores developed within the past 10 years are shown in Figure 1.

Here, we report a facile and efficient approach for the synthesis of heteroleptic iron(II) chromophores combining the bidentate strong-field NHC/pyridine ligands ^**Me**^**NHCpy** with the functional bidentate diimine 2,2′-bibenzimidazole (**BBIH**_**2**_). This allows the manipulation of ligand field properties through the peripheral diamine function of the ligand, specifically tuning of the redox potentials and photophysical properties of the respective complexes. Through femtosecond transient absorption spectroscopy (TA) in combination with density functional and time-dependent density functional theory (DFT and (TD)DFT) and resonance Raman spectroscopy (rR), we show how protonation of the ligand leads to a switching of the location of metal-to-ligand charge transfer (MLCT) excited states.

This protonation-induced switch of the MLCT is described by a **BBIH**_**2**_-centered MLCT state in the protonated complexes as opposed to an alternative ^**Me**^**NHCpy**-centered MLCT state in the deprotonated complex.^19−23^ Concomitantly, protonation is found to tip the balance of excited states in favor of the MLCT states relative to the less desired metal-centered (MC) excited states. The balancing of these excited states is a central challenge in the design of 3d transition metal chromophores, which, in the case of iron, thus far has been addressed almost exclusively by redesigning entire ligand scaffolds.^24−34^ Our study hence showcases a new strategy for the optimization of the excited-state landscape through manipulation of a precoordinated functional ligand in the second coordination sphere. The principle approach of combining NHCs as strong-field ligands and functional diimine ligands in heteroleptic architectures provides a convenient new route for faster and more efficient development of new and advanced iron(II) chromophores going forward.

## Results and Discussion

### Synthesis and Characterization

Only few examples of heteroleptic iron(II) chromophores with bidentate ligands are known to date (Figure 1).^35,36^ In the conventional approach to tris(bidentate) complexes with NHC ligands, a divalent salt such as FeCl_2_ is reacted with the imidazolium precursor of the carbene in the presence of a base. Under these conditions, the stoichiometry between ligand and metal cannot be controlled and gives low-to-moderate yields for the homoleptic tris-complexes. The plethora of ruthenium(II) chromophores, on the other hand, benefits from the availability of convenient and versatile precursors such as [(^R^bpy)_2_RuCl_2_] from which functional chromophores can be easily derived through substitution of the chloride ligands with a third bidentate ligand.^37^

Heteroleptic iron(II) chromophores with bis(tridentate) ligand architectures have been synthesized by using the iron precursor Fe(hmds)_2_ (hmds = hexamethyldisilamide). Using this dibasic precursor has the advantage of providing a strong base with the metal, hence avoiding the *in situ* formation of free carbene species in solution and controlling the stoichiometry between iron and NHC ligands to be 1:2.^38,39^ We adopted this strategy to access heteroleptic tris(bidentate) iron(II) chromophores based on NHC-pyridine ligands, which were previously explored by Gros et al.^24,40^ Reaction of the imidazolium salt ^**Me**^**NHCpy**H(PF_6_) with Fe(hmds)_2_ in acetonitrile yields the complex [(^Me^NHCpy)_2_Fe(NCMe)_2_](PF_6_)_2_ (**P1**) as a deep-red crystalline solid in 72% isolated yield.^41,42^**P1** crystallizes in space group *P*2_1_2_1_2_1_ as the respective Λ or Δisomer (Figure S66). Strikingly, the ^1^H NMR spectrum in deuterated acetonitrile (MeCN-*d*_3_) shows the formation of two isomers with *C_2_*- or C_1_-symmetry in a ratio of 3:2 as a result of flipping of one bidentate ligand (Figures S3–S5). This observation is consistent with reports by Gros et al. showing the formation of *mer*- and *fac-*isomers of the respective tris-homoleptic iron(II) chromophores of ^R^NHCpy ligands.^24,40^ Analogous iron(II) complexes were reported by Meggers et al., who used an electrochemical synthesis route and notably obtained stable isomers from aryl-substituted ^Ar^NHCpy ligands.^43−45^

Coordination of the 2,2′-bibenzimidazole was carried out in dimethylformamide (DMF), and the room temperature reaction yields two isomers with either *C*_*2*_ symmetry (NHC ligands both *trans* with respect to **BBIH**_**2**_ ligand), namely, **C2**, or *C_1_* symmetry (one ^**Me**^**NHCpy** ligand flipped compared to **C2**), namely, **C1**, in a 3:2 ratio (**C2**:**C1**).

**Figure 2:**
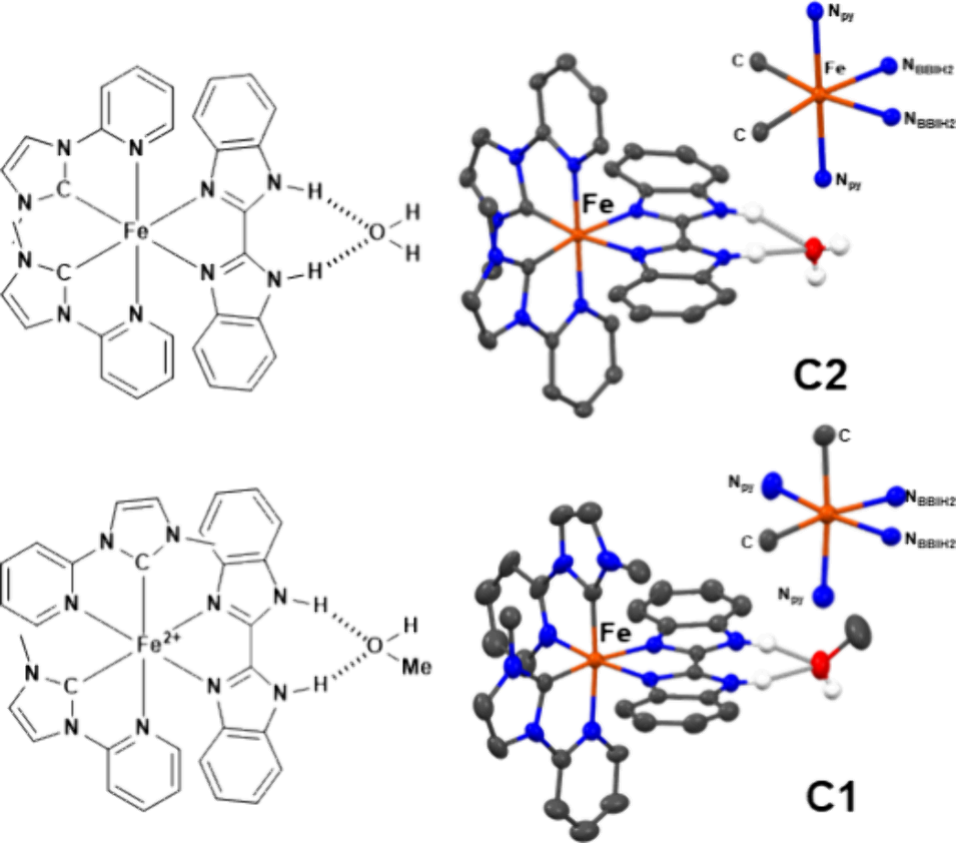
Schematic depiction and solid-state structures of heteroleptic complexes **C2** (top) and **C1** (bottom); structures depicted as balls-and-sticks, hydrogen atoms except those engaging in hydrogen bonds (H_2_O in the case of **C2**, MeOH in the case of **C1**), solvent molecules, and PF_6_ counterions omitted for clarity; octahedra showing the primary coordination spheres.

Separation of isomers was achieved using a mixture of ACN and methanol, from which **C2** and **C1** could be crystallized subsequently as hydrogen bond adducts with solvent molecules (see Figure 2). Careful optimization of the crystallization over several steps yielded isomerically pure samples of **C2** as small bright-red crystals and **C1** in roughly 96% isomeric excess as large dark-red crystals. In contrast, **C2** and **C1** were found to cocrystallize from acetone (see Figure S67). Both isomers were found to be stable toward isomerization in ACN solution according to UV–vis and ^1^H NMR spectroscopy (see Figures S90, S94, S99, and S100).

Single crystals suitable for X-ray diffraction were obtained for both isomers. Fe–C bonds *trans* to the bibenzimidazole are remarkably short (1.898 Å for **C1**, 1.900 and 1.902 Å for **C2**) compared to the Fe–C distance in **C1***trans* to pyridine of 1.920 Å. At the same time, the Fe–N_py_ bond *trans* to the carbene in **C1** is elongated (2.019 Å) compared to the Fe–N_py_ bonds in **C2** (1.979 Å, 1.993 Å), and the Fe–N_py_ bond *trans* to bibenzimidazole in **C1** is comparatively short at 1.898 Å. Fe–N_BI_ bonds are relatively long between 2.009 Å (*trans* to pyridine in **C1**) and 2.044 Å (*trans* to carbene in **C1**) due to the double five-membered ring structure of the two imidazole rings, which creates greater ligand strain. This leads to a characteristic bending of the **BBIH**_**2**_ ligand as characterized by the deviation from an ideal 180° angle of the two benzimidazole moieties (see Figure S71). The angles were determined as 13° for **C2** and 13.2° for **C1**, which is well within the range reported for analogous ruthenium complexes (12.8°–15.4°) despite the comparatively smaller ionic radius of iron(II).^19,46^

### Photophysics and Electrochemistry

**Figure 3:**
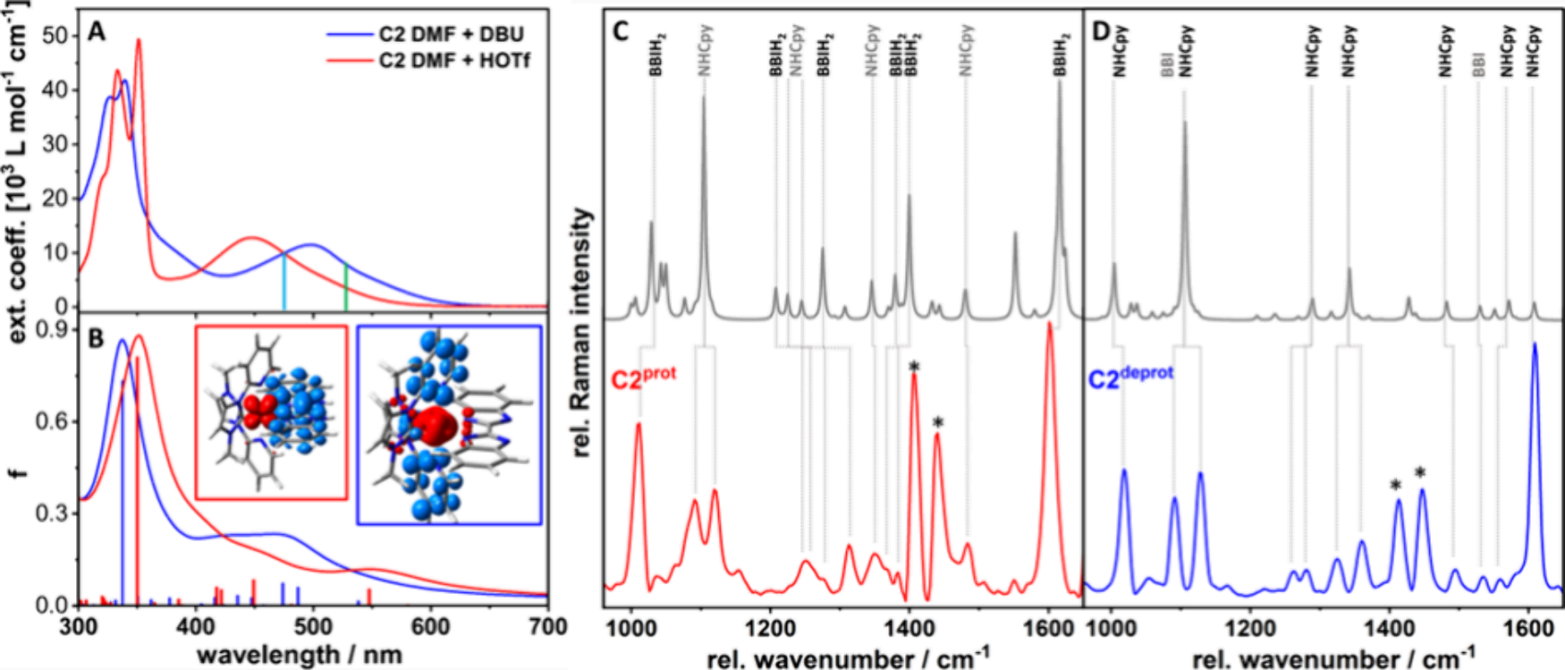
UV–vis absorption spectra of protonated (red) and deprotonated (blue) **C2** in DMF/HOTf and DMF/DBU, respectively, (A) and (TD)DFT-calculated UV–vis absorption spectra including calculated molecular orbitals charge density difference images illustrating the excited-state relocalization upon protonation for **C2** (B). RR excitation wavelengths are displayed as continuous vertical lines in the spectra. Comparison of the experimental (red for **C2**^**prot**^, panel C, and blue for **C2**^**deprot**^, panel D) and calculated (gray) rR spectra recorded at an excitation wavelength of 473 (**C2**^**prot**^) and 532 nm (**C2**^**deprot**^). Ligand-affiliated Raman modes are labeled with **BBI** and **NHCpy**. Bands marked with an asterisk are residues of solvent features due to the subtraction of the solvent spectrum; for **C1** see Figure S36.

Steady-state UV–vis absorption spectra of both isomers in ACN exhibit sharp ligand-centered absorptions at 331 and 349 nm as well as a broad MLCT absorption band in the blue (see Figures S23 and S24). The MLCT absorption maximum of **C2** was found to be at 444 nm. **C1**, in contrast, exhibits a broader MLCT band with a slightly blue-shifted absorption maximum at 439 nm and a shoulder centered at 520 nm extending further to the red. Due to limited solubility upon deprotonation, further experiments of the protonation dependence were carried out in DMF (Figures 3A and Figure S36A, also Figures S25 and S26). Strikingly, the initial UV–vis absorption spectra show much broader and unstructured MLCT absorptions for both isomers in DMF solution, which was attributed to hydrogen bond interactions with the solvent. Addition of trifluoromethanesulfonic acid (HOTf) yields the structured features observed in ACN as discussed above. Upon deprotonation with excess base 1,8-diazabicyclo[5.4.0]undec-7-en (DBU), the MLCT transitions become significantly broadened and shifted bathochromically to 506 nm (Figure 3A and Figure S36A, also Figures S25 and S26).

This behavior is explained by a cathodic shift of the Fe^2+/3+^ redox process due to increased σ,π-donation from the deprotonated 2,2′-bibenzimidazolate ligand (see Tables S7–S10, Figure S34).^21,23^ In the case of **C1**, this shift was determined as 473 mV, while a notably larger shift of 534 mV was obtained for **C2** indicating a greater influence of protonation on the latter. Notably, for the protonated complexes, the Fe^2+/3+^ redox couple appears at roughly the same voltage for both isomers (0.098 V for **C1**, 0.087 V for **C2**, values in DMF referenced versus Fc/Fc^+^).

To elucidate the impact of deprotonation on the electronic structure and assess the nature of the electronic transitions, (TD)DFT simulations were performed (Figure 3B and Figures S36B, S38, and S43). In the case of the deprotonated species in DMF, the red-shifted visible absorptions can be related predominantly to a d_Fe_ →π*_NHCpy_ MLCT transition, namely, into S_5_, at 503 and 487 nm for **C1**^**deprot**^ and **C2**^**deprot**^, respectively. Further dipole-allowed MLCT transitions into S_9_, S_11_, and S_13_ (**C1**^**deprot**^) and S_6_, S_9_, and S_13_ (**C2**^**deprot**^), related to the population of **^Me^NHCpy** ligands, were also identified.

Upon protonation, the MLCT absorptions related to d_Fe_ →π*_NHCpy_ transitions are blue-shifted to 460 nm (**C1**^**prot**^) and 449 nm (**C2**^**prot**^), which fits well with the experimentally observed spectra (Figure 3A). In contrast, the calculations also indicate a significant red-shift of d_Fe_ →π*_BBIH2_ transitions from 416 nm (**C2**^**deprot**^, S_17_) and 445 nm (**C1**^**deprot**^, S_11_) to 547 nm (**C2**^**prot**^, S_3_) and 556 nm (**C1**^**prot**^, S_3_, Figure 3B). Notably, the transition dipole moments for these latter transitions (S_0_ → S_3_) are likely slightly overestimated, with respect to the experimental data.

To identify the ligands involved in the respective MLCT transitions experimentally, resonance Raman (rR) studies were performed. Excitation of **C1^prot^** and **C2^prot^** at 473 nm reveals prominent bands between 1600 and 1000 cm^–1^ (Figure 3C bottom and Figure S36C, red labeled Raman bands).^47^ These features are mainly assigned to **BBIH**_**2**_-based vibrations and agree excellently with the calculated rR pattern of **C1**^**prot**^ and **C2**^**prot**^ (Figure 3C, top, Figure S36C, top, also Figure S65, and Tables S34 and S36). Thus, the lowest energy MLCT transition results in charge transfer to the protonated bibenzimidazole ligand. In contrast, intensive features at 1608, 1559, 1495, 1357, 1090, and 1018 cm^–1^ were observed for **C2**^**deprot**^ (Figure 3D) and **C1**^**deprot**^ (Figure S 36D), which are associated with the ^**Me**^**NHCpy** ligand based on the non-resonant reference compound **P1** and supporting theoretical calculations (Figures S36 and S37). (TD)DFT calculations relate these features to a predominant population of the MLCT transitions from the iron(II) center to the ^**Me**^**NHCpy** ligand sphere, while MLCT_BBI_ transitions are shifted to higher excitation energies upon deprotonation (**C1** and **C2**, see Tables S33 and S35).

**Figure 4:**
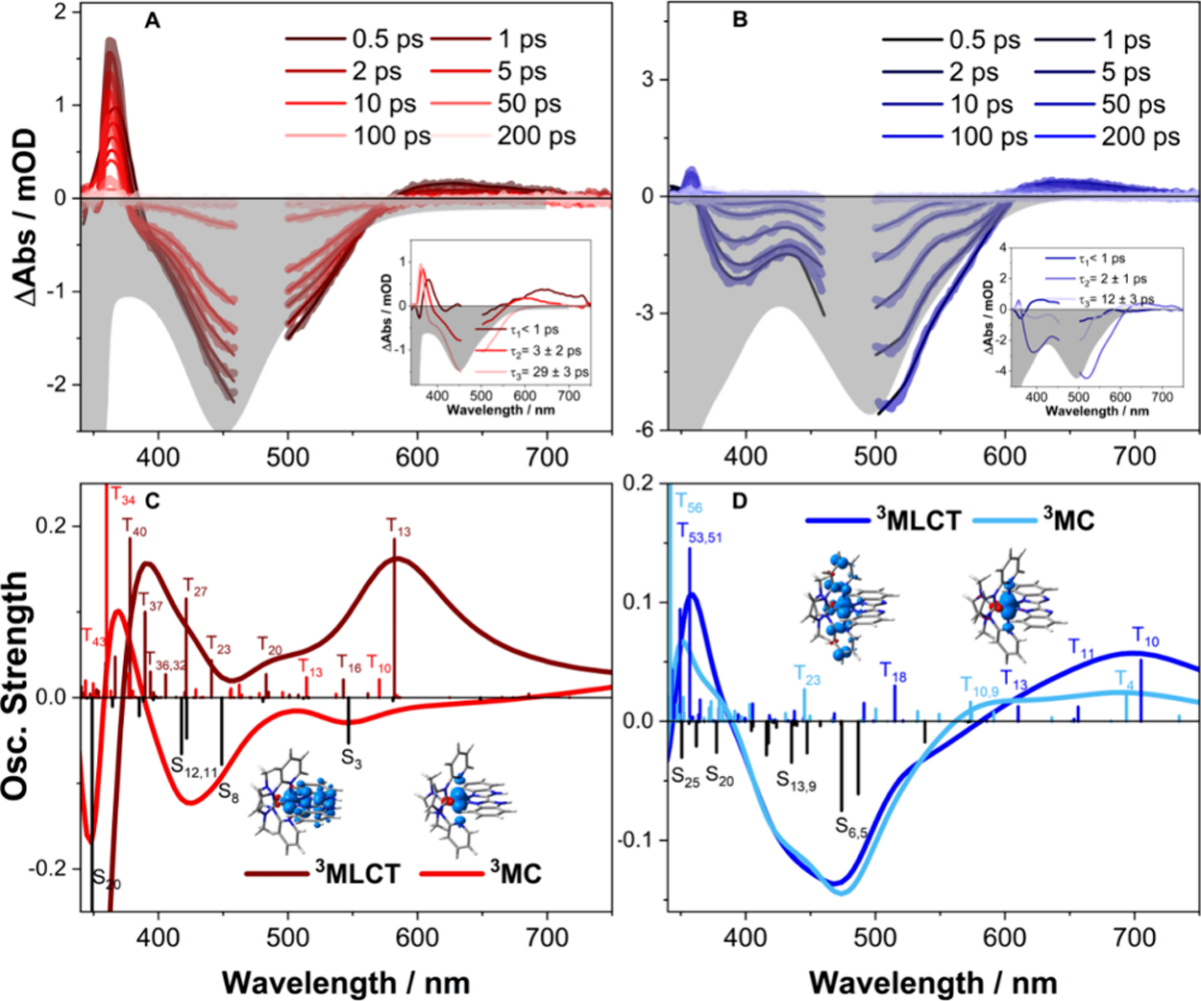
Transient absorption spectra at selected delay times (A,B), the corresponding DAS as inset and inverted steady-state absorption as gray area corresponding to **C2**^**prot**^ (A, red: DMF+HOtf); and **C2**^**deprot**^ (B, blue: DMF+DBU). Simulated TAS at the (TD)DFT level of theory of **C2**^**prot**^ in ACN (C) and **C2**^**deprot**^ in DMF (D).

Femtosecond transient absorption was recorded for all species **C1** (Figure S27A,C) and **C2** (Figure 4A,B) in DMF in the respective protonated and deprotonated states. For the protonated complexes (Figure 4A and Figure S27A), excitation at 470 nm leads to an instantaneous ground-state bleach (GSB) between 380 and 600 nm accompanied by an excited-state absorption (ESA) signal at about 370 nm and a weak, broad excited-state absorption feature from 600 to 750 nm. The ESA in the blue region at approximately 370 nm along with broad ESA signal in the red region is typically interpreted as signatures of MLCT excited states.^16,18^ All transient features gradually decay over a period of 100 ps, while the weak ESA feature in the red region rapidly disappears within the first few ps. Based on the absence of slow ground-state recovery in the 100 ps-to-ns range typically associated with relaxation via ^5^MC states, we assume that the ^5^MC states are significantly destabilized and do not play a role in the excited-state physics.

For the protonated species, both DFT and TDDFT methods were employed to characterize the lowest energy triplet state to be of a ^3^MLCT_BBIH2_ nature.

Assignment of transient features on the picosecond time scale is challenging especially for iron(II) chromophores and needs to be handled with caution.^51^ The availability of a small group of closely related compounds in this case enabled a more reliable distinction between features and artifacts and gives higher fidelity to the simulation of TA features by quantum chemical means.

The simulated TAS of complex **C2**^**prot**^ within the equilibrated ^3^MLCT_BBIH2_ geometry in ACN reveals that the ESA in the region between 370 and 450 nm is attributed to several mixed charge transfer transitions, while the prominent ESA at 582 nm is associated with an interligand-charge-transfer (^3^ILCT_BBIH2_) state (T_13_; π_**BBIH2**_ → π*_BBIH2_), see Figure S57, Table S28. In a similar fashion, the TAS of **C1**^**prot**^ within the equilibrated ^3^MLCT in ACN features an ESA between 370 and 450 nm, due to mixed ^3^MLCT and ^3^ILCT transitions (i.e., T_41_ and T_43_), and a prominent ESA band at 579 nm, related to a ^3^ILCT_BBIH2_ transition (T_12_), see Figure S51 and Table S22.

In comparison, the TAS of the **C1**^**prot**^ and **C2**^**prot**^ obtained within the lowest fully relaxed ^3^MC state shows almost no ESA within the visible region, as the electronic structure of the chromophore is mostly unaffected by the spin flip at the metal center. For **C1**^**prot**^, prominent spin and dipole-allowed triplet–triplet signals are predicted at ∼340–360 nm. These transitions are mainly of the ^3^MLCT character (T_35–37_) with an admixture of ^3^LLCT transitions (T_40–42,44_) (Figure S52, Table S23). In the case of the ^3^MC structure of **C2**^**prot**^ the signals at 320–330 and 360–380 nm can be associated with ^3^MLCT (T_30,45_), ^3^ILCT (T_34_), and ^3^LMCT (T_41,43_) transitions based on the performed quantum chemical simulations (Figure S58, Table S29). Unlike the ^3^MC species, the simulated ESA contributions within the ^5^MC equilibrium yield dipole-allowed ^5^MLCT_NHCpy_ and ^5^MLCT_BBIH2_ transitions in the visible region (see ^5^MLCT states Q_5_, Q_6_, and Q_10_ for **C1**^**prot**^ and states Q_6_ and Q_9_ for **C2**^**prot**^ in Figures S53 and S59, Tables S24 and S30). Thus, the calculations allow us to assign distinct spectral characteristics as well as their relative energies stemming from ^3^MLCT, ^3^MC, and ^5^MC contributions, which in turn provides the basis for the assignment of these states during the excited-state relaxation. **C1**^**deprot**^ shows an ESA in the UV region at around 300–350 nm in both ^3^MC and ^5^MC geometries. Unfortunately, the ^3^MLCT structure of **C1**^**deprot**^ could not be optimized (see the Supporting Information for details). In all other cases, the lowest energy ^3^MLCT was fully relaxed at the TDDFT level of theory and reoptimized using (unrestricted) DFT. Thereby, both methods, i.e. DFT and TDDFT, allow to draw a consistent picture and yield equilibrated ^3^MLCT energies within a range of 1.5 and 1.8 eV. Among the metal-centered states of interest, the ^3^MC states are observed with relative energies ranging in a rather close energy window from 0.7 to 0.8 eV. The respective ^5^MC equilibrium structures are well above the ^3^MC states with energies of 1.3–1.4 eV. Thus, and as the population of the quintet states would only be possible upon a second ISC process, we conclude that the population of such quintet MC states is of minor importance for the present photoactive Fe(II) complexes.

**Figure 5:**
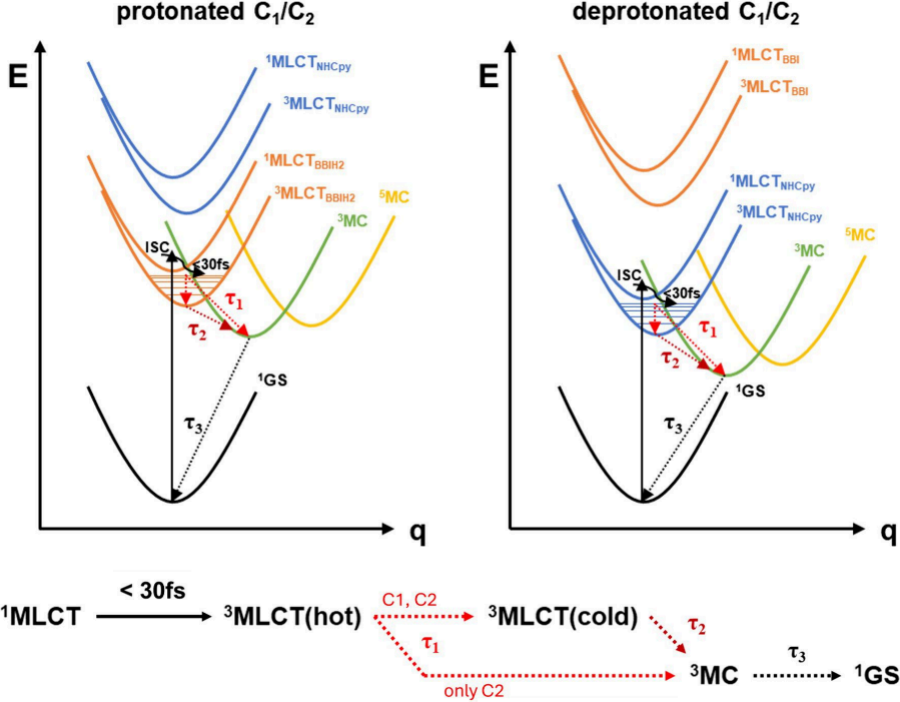
Top: simplified Jablonski diagrams for the protonated (left) and deprotonated complexes (right) showing important excited states and the reaction cascade occurring after ^1^MLCT excitation. According to (TD)DFT calculations and rR measurements, the ^1/3^MLCT involving the BBIH_2_ ligand is lower-lying for protonated **C1/C2**, while for deprotonated **C1/C2**, the ^1/3^MLCT involving the NHCpy ligands is lower-lying. Bottom: excited-state dynamics and lifetimes in the “hot-branching” model after ^1^MLCT excitation. **C2** branches from ^3^MLCT(hot) into ^3^MLCT(cold) and ^3^MC(cold), while **C1** mainly relaxes via vibrational cooling from the “hot” ^3^MLCT to the “cold” ^3^MLCT.

Global analysis of the TA data using a three-exponential model was done for all four species (inset in Figure 4A,B for **C2** and Figure S27D–F for **C1** and Table S3). Lifetimes τ_1_, τ_2_, and τ_3_ are interpreted based on the “hot-branching” model as summarized in Figure 5 based on the decay associated spectra (DAS, inset in Figure 4A,B) as elaborated below, and supported by simulated TA spectra using TDDFT methods with the exception of **C1**^**deprot**^.^48,49^

Initial excitation at 470 nm populates the ^1^MLCT state followed by intersystem crossing to a vibrationally excited ^3^MLCT(hot) state, which is reported to happen in less than 30 fs, i.e., within the temporal resolution of the experiment.^50^ Following this, τ_1_ describes an ultrafast process (<1 ps), whose spectral changes reflect the buildup of a weak and narrow ESA signal at 360 nm (associated with buildup of population of ^3^MC). Concurrently, a decay of ESA at 380 nm and a blue shift of the ESA at 650 nm are associated with vibrational relaxation within the triplet MLCT manifold. This process is interpreted as an excited-state branching where the vibrationally excited ^3^MLCT(hot) state simultaneously transforms into both a relaxed ^3^MLCT(cold) and a ^3^MC. τ_2_ describes the decay of the ESA band at 370 nm and the blue-shifted ESA band at 600 nm and the GSB signal. We ascribe the process associated with τ_2_ to a population transfer from the ^3^MLCT(cold) state into the ^3^MC state. Finally, the spectral characteristics associated with τ_3_ reflect the decay of the ESA band at 365 nm and the major contribution to GSB recovery, with virtually no more contributions from the red ESA. This has been attributed to the relaxation of ^3^MC back to the ground state. To obtain values for the branching ratio Φ of our previous discussed model, we performed target analysis. The branching ratio Φ denotes the fraction of molecules in ^3^MLCT(hot) undergoing vibrational relaxation relative to the ^3^MLCT(cold) state.

Using the lifetimes calculated by global analysis as input for the target model, branching ratios of 1 for **C1** and 0.5 for **C2** are obtained. Therefore, we conclude that photoexcited **C1** mainly relaxes via initial cooling from the ^3^MLCT(hot) to ^3^MLCT(cold). **C2** on the other hand branches from the ^3^MLCT(hot) both into ^3^MLCT(cold) and ^3^MC as discussed (see Supporting Information for more details).

The TAS of the deprotonated species (Figure 4B for **C2**^**deprot**^, Figure S27C for **C1**^**deprot**^) exhibits the same spectral characteristics, with the difference that the intensity of the ESA band at 370 nm is diminished with respect to the GSB compared to the protonated species. The TAS of parent complex **P1**, which has only the ^**Me**^**NHCpy** ligand without the **BBIH**_**2**_ ligand lacks the prominent ESA band at 370 nm, suggesting that this band can be assigned to transitions mainly involving the **BBIH**_**2**_ ligand (see Figure S29). This observation explains the diminishment of the ESA band at 370 nm upon deprotonation, where we predominantly populate the orbitals associated with ^**Me**^**NHCpy**, aligning with the findings from the theoretical calculations.

When comparing the DAS, in terms of spectral simulations, all three components of both **C1**^**prot**^ (Figure S27D) and **C1**^**deprot**^ (Figure S27F) mirror the same behavior as that of **C2**^**prot**^ (inset in Figure 4A). Therefore, we assign the same process to all three components. Kinetic analysis of **C2**^**deprot**^ (inset in Figure 4B) indicates that, while τ_1_ reflects the same spectral behavior as for the protonated species, τ_2_ and τ_3_ show slightly different behavior. The spectral characteristics associated with τ_2_ reveal a larger GS recovery compared to τ_3_, which in turn has additional spectral features with some remaining blue-shifted ESA contributions at 600 nm. This suggests the presence of a direct non-radiative relaxation path both from ^3^MLCT and ^3^MC to the singlet ^1^GS.

### Isomerism Versus Protonation/Deprotonation

Introduction of functional ligand **BBIH**_**2**_ as a model functional ligand demonstrates the ability to modify photophysical properties in iron chromophores beyond the manipulation of ligand architectures and the first coordination sphere. This is showcased by us through protonation/deprotonation as a simple and reversible mode of manipulating the ligand properties and, subsequently, the ligand field.^52^ However, it is worth noting that bibenzimidazole complexes can be manipulated through other supramolecular effects, such as coordination of cations or the formation of hydrogen bonds. This could hence be used to construct supramolecular architectures and fine-tune photophysical properties.^20,53,54^

In addition to introducing the functional **BBIH**_**2**_ ligand, we have also obtained the two isomers **C1** and **C2**, which differ from each other based on their primary coordination sphere. This provides us with the opportunity to directly compare and evaluate the effects of both first- and second-sphere manipulations on the excited-state landscape.

As discussed above, lifetimes τ_2_ and τ_3_ (Table S3) are tentatively associated with the ^3^MLCT and ^3^MC states, respectively. Both lifetimes are consistently found to be longer in **C1** compared to **C2**, which is most clearly visible in the longer component τ_3_ that was determined to 63 ± 9 ps in **C1**^**prot**^ compared to 29 ± 3 ps in **C2**^**prot**^. We interpret this as a consequence of the carbene ligands both occupying equatorial positions in **C2**, which formally opens up a deactivation channel on the *z*-axis defined by the pyridine ligands. In contrast, in **C1**, one NHC ligand occupies the equatorial plane, whereas the other occupies one of the axial positions. In this lower symmetry ligand field, all accessible ^3^MC states are higher in energy, which leads to the absence of a “hot-branching” mechanism as discussed above (Φ = 1), slower population of the ^3^MC from the ^3^MLCT(cold) and thus a longer ^3^MLCT lifetime for **C1**. The longer ^3^MC lifetime is explained as a consequence of an increased energy difference between ^3^MC and ^1^GS following the *energy gap law* (cp processes described in Figure 5).

For both **C1** and **C2**, protonation is found to lead to an increase in all lifetimes. We explain this by stabilization of the **BBIH**_**2**_ π*-acceptor orbital upon protonation in combination with a reduction of its σ,π-donor strength, thus creating a stronger ligand field. This also correlates to the *MLCT switch*, as described above. While both τ_2_ and τ_3_ show a systematic increase, only the values for τ_3_ are discussed since changes for τ_2_ are too small relative to the margin of error. Notably, in the case of **C2**, protonation is found to have a stronger effect on the excited-state lifetimes. Namely, for **C2**^**prot**^ τ_3_ was determined as 29 ± 3 ps compared to 12 ± 3 ps for **C2**^**deprot**^, which corresponds to a 2.4-fold increase. In contrast, for **C1**, the lifetime τ_3_ is increased by only about 10% from 57 ± 7 ps for **C1**^**deprot**^ to 63 ± 9 ps in **C1**^**prot**^. This indicates a more dominant effect of the primary coordination sphere in **C1** compared to **C2**. The lesser effect of protonation on the photophysics of **C1** may be explained by the generally more destabilized ^3^MC states, as discussed before. In addition, the stronger anodic shift of the Fe^2+/3+^ redox couple (*vide supra*) in **C2** should also be considered, as it likely corresponds to a higher degree of destabilization of metal-centered states.

**Figure 6:**
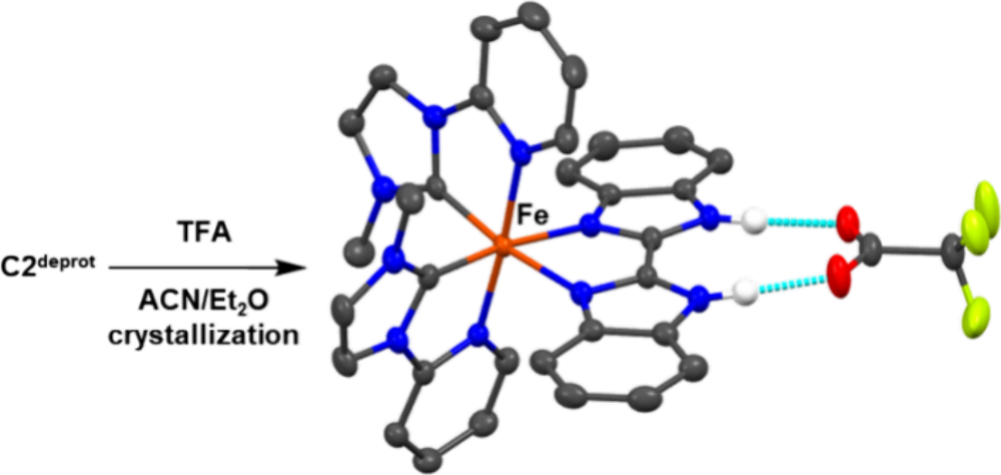
Solid-state structure of **C2**^**prot**^ trifluoroacetate salt obtained from protonation of **C2**^**deprot**^ showing the bifurcated hydrogen bond between cation and one anion; hydrogen atoms except those engaging in hydrogen bonds, and counterion trifluoroacetate omitted for clarity.

Notably, the highest lifetime attributed to the ^3^MLCT state, namely, τ_2_, was determined as 9 ± 6 ps, which is comparable to iron chromophores that have been used in photochemical applications such as photocatalytic hydrogen formation and photovoltaics.^34,39,55^ In the latter case, immobilization of the chromophore on the electron-accepting semiconductor surface was shown to enable efficient electron injection. Similarly, we anticipate the use of hydrogen bonds through the **BBIH**_**2**_ ligand to construct photochemical donor–acceptor pairs to salvage the short-lived excited states in our iron chromophores. To showcase the approach, we reacted deprotonated complex **C2**^**deprot**^ with trifluoroacetic acid to obtain the respective trifluoroacetic acid salt, which could be crystallized to obtain a solid-state structure with the anticipated bifurcated hydrogen bond framework (Figure 6). Fe–C bonds (1.894 Å, 1.941 Å), Fe–N_py_ bonds (1.994 Å, 1.972 Å), and Fe–N_BI_ bonds (2.045 Å, 2.056 Å) compare well with the structures above; the bending of **BBIH_2_** ligand toward the metal is slightly extended (14.03°).

The N–O distances characterizing the hydrogen bonds (2.658 Å, 2.693 Å) compare well to the analogous ruthenium(II) complex (2.665 Å).^19^ The resonance for the N–H protons in the ^1^H NMR spectrum measured in ACN-*d*_3_ is strongly shifted to 16.3 ppm, compared to 12 ppm for the PF_6_ salt, indicating a significant interaction also in solution.

### Reactivity Studies

**Figure 7:**
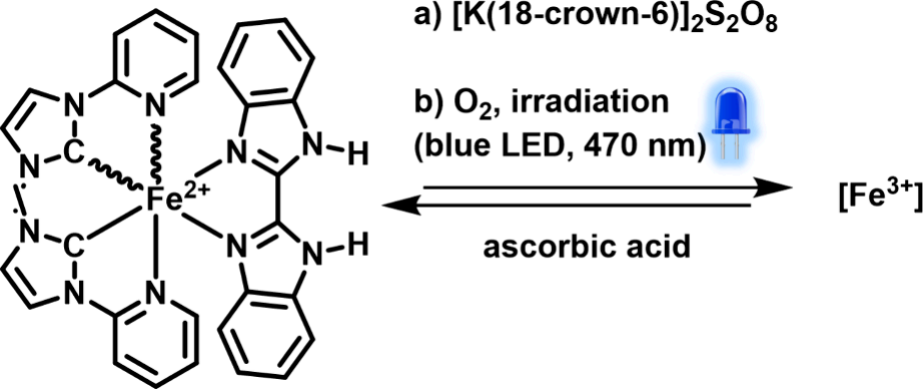
Redox reactivity of **C1** and **C2**; (a) oxidation by peroxodisulfate in ACN was tracked over 6 h using UV–vis spectroscopy; (b) incomplete oxidation by oxygen is observed under irradiation with an LED at 470 nm; the iron(II) species is regenerated upon reduction of the respective iron(III) complex with ascorbic acid.

Complexes **C1** and **C2** were oxidized using peroxodisulfate, a common sacrificial electron acceptor in photoredox catalysis (see Figure 7). Complexes **C1**^**prot**^ and **C2**^**prot**^ were mixed with a 10-fold excess of 18-crown-6 potassium peroxodisulfate in dry ACN, and the reaction was tracked using UV–vis spectroscopy (see Figures S74–S78).^56^ Oxidation of both isomers was observed over a period of 6 h and is indicated by a loss of the MLCT absorption band and the appearance of a broad low-intensity absorption spanning from 600 to 800 nm with a maximum at roughly 680 nm (Figures S74–S82). These features agree well with calculated absorption spectra for oxidized **C2** (Figure S63). Interestingly, oxidation of **C1** was found to happen slightly faster compared to that of **C2**.

We subsequently also investigated the stability of **C1** and **C2** in ACN solution (i) under aerobic versus anaerobic conditions, and (ii) under irradiation at 470 nm versus exclusion of light. Partial oxidation as characterized by a loss of the MLCT absorption band (∼20% for both isomers within 72 h, Figure S86) and appearance of a new broad absorption band spanning from 600 to 800 nm (Figures S83 and S84) was only observed in the presence of both light and oxygen, hence showing a first example of photoredox reactivity for these new chromophores. Photooxidation is also indicated by significant line-broadening in the respective ^1^H NMR spectra when samples were irradiated under air (Figures S97 and S98).

Addition of ascorbic acid to fully oxidized samples of **C1** and **C2** (obtained from oxidation with peroxodisulfate after 18 h reaction time, Figures S79 and S80) recovered the spectral features of the reduced complexes as indicated by the characteristic MLCT absorption bands and the two diagnostic ligand-based absorptions in the UV range, hence demonstrating full reversibility of the iron-based redox chemistry.

## Conclusions

Introduction of the functional 2,2′-bibenzimidazole ligand through a facile and efficient synthetic route toward heteroleptic iron(II) chromophores is demonstrated to enable the manipulation of excited states in the respective complexes *via* protonation of the coordinated ligand in the second coordination sphere. Based on isomerization of the bidentate carbene ligand ^**Me**^**NHCpy**, we isolated the two complexes **C1** and **C2** that exhibit strong visible light absorption features associated with MLCT transitions. The position of the carbene ligands in isomers **C1** and **C2** is shown to have only a minor effect on the ground-state properties but a significant effect on the excited-state lifetimes. In contrast, deprotonation of the **BBIH**_**2**_ ligand is shown to cause a significant red-shift of the MLCT absorption, which is associated with an *MLCT switch* from a d_Fe_ →π*_BBIH2_ to a d_Fe_ →π*_NHCpy_ type transition. Protonation, in contrast, gives rise to the **BBIH**_**2**_-centered MLCT state, which is slightly more favored with respect to MC states as compared to the ^**Me**^**NHCpy**-centered MLCT state in the deprotonated complex. Femtosecond transient absorption spectroscopy, quantum chemical simulations, and resonance Raman spectroscopy were employed to resolve the excited-state dynamics of these new chromophores and identify three distinct lifetimes leading back to the ground state within a 100 ps time window. The underlying processes were described by the “hot-branching” model with ps-MLCT lifetimes. Remarkably, whereas deprotonation of the coordinated ligand is shown to lead to faster deactivation through lower-lying ^3^MC states, protonation is shown to lead to significantly prolonged lifetimes associated with a **BBIH**_**2**_-localized MLCT state. This fine-tuning of excited states through manipulations of a coordinated functional ligand in the second coordination sphere creates new opportunities for new design approaches for iron(II) MLCT chromophores beyond ligand field manipulations in the primary coordination sphere. The facile synthetic route shown here promises to accelerate the development of new and more versatile iron(II) chromophores in the future and to extend the potential use of these complexes in supramolecular chemistry. The reported complexes showcase the potential for supramolecular integration through the formation of hydrogen bonds, as observed in the reported solid-state structures. Ongoing research focuses on salvaging this feature in combination with the observed **BBIH**_**2**_-located ^3^MLCT for the construction of photoactive supramolecular assemblies and materials.

## Supporting Information Available

Details about the methods and materials used in this study, spectroscopic and spectrometric data, (TD)DFT calculations, and scXRD data are provided in the Supporting Information (PDF)
