# Supplementary material for: Beyond the First Coordination Sphere—Manipulating the Excited-State Landscape in Iron(II) Chromophores with Protons
Source: J Am Chem Soc. 2024 Jul 11;146(29):19710–9. doi: 10.1021/jacs.4c00552 (PMC11273614; doi:10.1021/jacs.4c00552)
Supplement: Supplementary file 1 — ja4c00552_si_001.pdf [file ja4c00552_si_001.pdf]

Supporting Information

Beyond the first coordination sphere – manipulating the excited state landscape in iron(II) chromophores with protons

Authors: Kamil Witas‡, Shruthi Santhosh Nair‡, Tamar Maisuradze‡, Linda Zedler, Heiner Schmidt, Pablo Garcia-Porta, Alexandra Stefanie Jessica Rein, Tim Bolter, Sven Rau, Stephan Kupfer*, Benjamin Dietzek-Ivanšić*, Dieter U. Sorsche*

Content

1. Materials 3

2. Instrumentation and Methods 4

2.1 Cyclic Voltammetry 4

2.2 High Resolution Mass Spectrometry (HRMS) 4

2.3 NMR Spectroscopy 4

2.4 UV/Vis Spectroscopy 4

2.5 XRD Measurements 4

2.6 Computational Details 5

2.7 Resonance and non-resonant Raman Spectroscopy 6

2.8 Transient Absorption Spectroscopy 7

2.9 Stability and reactivity measurements: 10

3. Synthesis 11

3.1 Synthesis of Ligand L1 11

3.2 Synthesis of Complex P1 11

3.3 Synthesis of Complexes C1 and C2 12

3.3.1 Isolation of C2 13

3.3.2 Isolation of C1 13

3.3.3 Preparation of deprotonated complexes for cyclic voltammetry 14

3.3.4 Preparation of single crystals of C2^{prot}-TFA 15
3.4 Preparation of $[(18\text{-crown-6})K]_2S_2O_8$ 16
4. NMR Spectroscopy 17
5. Mass Spectrometry 26
6. Steady-state UV/Vis Spectroscopy 27
7. Transient Absorption Spectroscopy 31
8. Electrochemical Investigations 35
9. Raman Spectroscopy 40
10. Theoretical Calculations 41
11. Crystallography 82
12. Reactivity Studies 91
12.1 Oxidation of C1 and C2 with $[(18\text{-crown-6})K]_2S_2O_8$ 91
12.2 Photostability 99
12.3 Counter ion exchange 108
13. References 109

1. Materials

If not otherwise stated all solvents for the synthesis were 99.9+ and extra dry from ACROS Organics and used as bought. 2-bromopyridine 99% was purchased from ACROS Organics, 1-methylimidazole 99% was purchased from Sigma Aldrich, NH_4PF_6 99.5% was purchased from abcr.

$Fe(HMDS)_2$ was synthesized according to literature,[1] as well as bibenzimidazole.[2]

2. Instrumentation and Methods

2.1 Cyclic Voltammetry

All measurements were performed in a nitrogen glove box under room temperature with a scan rate of 100 mV/s. 10 mL of a 0.1M [nBu_4N][PF_6] solution was used as the electrolyte with an analyte concentration of 1 mM. A silver wire inside a frit was used as the reference electrode, a platinum wire was used as the counter electrode and glassy carbon electrode was used as the working electrode.

2.2 High Resolution Mass Spectrometry (HRMS)

Mass spectrometry was performed at the mass spectrometry service department of Ulm University by Mr. Dr. Markus Wunderlin, using a Fourier Transform Ion Cyclotron Resonance (FT-ICR) mass spectrometer solariX (Bruker Daltonics) equipped with a 7.0 T superconducting magnet and interfaced to an Apollo II Dual ESI/MALDI source. For all MALDI measurements trans-2-[3-(4-tert-butylphenyl)-2-methyl-2-propenylidene]malononitrile (DCTB) was used as the matrix. Spectra were analyzed with Compass Data Analysis Viewer Version 4.4.

2.3 NMR Spectroscopy

NMR spectroscopy was performed either on a Bruker Avance 600 MHz or Bruker Avance 400 MHz spectrometer. The shift values are given in ppm and are referenced to the corresponding solvent residual peaks.

2.4 UV/Vis Spectroscopy

Extinction coefficient measurements were performed on a Horiba Duetta with EzSpec device placed in a nitrogen atmosphere glovebox Optical quartz glass cuvettes with a pathlength of 10 mm were used.

2.5 XRD Measurements

Crystals suitable for X-ray crystallography were mounted using a MicroLoop and Perfluoropolyalkyl ether (viscosity 1800 cSt). X-ray diffraction intensity data were measured at 150 K on a Bruker D8 Quest single crystal diffractometer with a PHOTON II detector using Mo - Kα radiation (wavelength λ = 0.71073 Å). Structure solution and refinement was carried out using the SHELXL package[3,4] via Olex2. Corrections for incident and diffracted beam absorption effects were applied using multi-scan refinements. Structures were solved by direct methods and refined against F2 by the full-53 matrix least-squares technique. The hydrogen atoms were included at

calculated positions with fixed thermal parameters. All non-hydrogen atoms were refined anisotropically unless otherwise mentioned. MERCURY was used for structural representations.[5]

2.6 Computational Details

The structural and electronic properties of the two isomers, **C1** and **C2**, of the iron(II) complex were studied in their protonated and deprotonated states using the Gaussian 16 package.[6] Fully relaxed equilibrium geometries within the singlet ground state (S_0, GS) were obtained at the density functional level of theory (DFT) using a functional which is denoted B3LYP10. This functional based on B3LYP[7,8], comprises 10% of exact-exchange, respectively, 58.5% of non-local B88 exchange and the LYP correlation. The def2-SVP[9,10] basis set was applied for all atoms. A vibrational analysis was carried out for each GS geometry to verify the correspondence of ground state geometries to minima on the respective potential energy surfaces (PES). For all calculations Grimme's dispersion correction with Becke-Johnson damping (D3BJ)[11] was utilized.

Excited state properties of the 100 lowest singlet excited states such as excitation energies, oscillator strengths and electronic characters were obtained at the previously obtained ground state equilibria by applying the same XC functional and basis set at time-dependent DFT (TDDFT) level of theory. Furthermore, the fs-transient absorption spectra of all complexes were modeled with assumption of 1:1 population of singlet and respective triplet or quintet ground states. Therefore, the excited-state absorption (ESA) was predicted based on the first 100 spin and dipole-allowed triplet-triplet transitions within the triplet ground state geometries (^{3}MC and ^{3}MLCT) as well as by means of quintet-quintet excitations within the quintet ground-state (^{5}MC) geometry. The equilibrium structures of the lowest ^{3}MC and ^{3}MLCT and ^{5}MC geometries were obtained by DFT. The equilibrated ^{3}MLCT structure of **C1^{deprot}** could not be obtained by several different methods. Ground state bleach (GSB) was accounted for by the singlet-singlet absorption within the Frank-Condon geometry (S_0). This computational setup has been successfully applied[12–19] to elucidate UV-vis absorption, transient absorption, resonance Raman, (spectro-)electrochemistry, and electron transfer dynamics, as it allows a balanced description of the ground and excited state properties in transition metal complexes.

Implicit solvent effects (acetonitrile ACN: ε = 35.688, n = 1.344; *N,N*-dimethylformamide, DMF: ε = 37.219, n = 2.046) were accounted for using the solute electron density (SMD) variant of the integral equation formalism of the polarizable continuum model[20] in case of all simulations. The non-equilibrium procedure of solvation was used for the calculation of the excitation energies within the Franck-Condon point, which is well adapted for processes where only the fast reorganization of the electronic distribution of the solvent is important. Triplet-triplet and quintet-quintet excited state calculation of protonated complexes were exclusively carried within ACN, while for deprotonated complexes DMF was utilized.

Resonance-Raman (rR) spectra were simulated based on the independent mode displaced harmonic oscillator model (IMDHOM), which assumes the potential energy surfaces of the electronic ground- and excited-states to be harmonic and sharing the same set of vibrational coordinates and vibrational frequencies. Detailed information on the computational protocol to simulate rR spectra within the IMDHOM method can be found in the following references [21–23]. The rR spectra of the protonated and deprotonated species were calculated at excitation wavelengths of 532 and 473 nm. Thereby, the dipole-allowed electronic transitions in the visible spectral region were considered to contribute to the rR intensity pattern, while the rR intensity of each normal mode depends on the transition dipole moment, the excitation energy as well as on the excited state gradient. A dumping factor of 0.1 eV was used. The following states were taken into account for **C1**$^{\mathbf{deprot}}$ (S_5, S_8, S_9, S_{11}, S_{13}, S_{14}, S_{16}, S_{17}, S_{23}, S_{24}, S_{26}, S_{27}, S_{28}, S_{29}, S_{30}), **C2**$^{\mathbf{deprot}}$ (S_3, S_5, S_6, S_9, S_{13}, S_{16}, S_{17}, S_{23}, S_{24}, S_{25}, S_{28}, S_{29}, S_{30}), **C1**$^{\mathbf{prot}}$ (S_3, S_8, S_9, S_{13}, S_{14}, S_{15}, S_{16}, S_{18}, S_{20}, S_{22}) and **C2**$^{\mathbf{prot}}$ (S_3, S_8, S_{11}, S_{12}, S_{16}, S_{18}, S_{20}), respectively.

2.7 Resonance and non-resonant Raman Spectroscopy

Protonation dependent Resonance Raman (rR) measurements of the heteroleptic iron(II) chromophores **C1** and **C2** were performed through excitation by a 473 nm and a 532 nm diode pumped solid state laser lasers (HB-Laser, Germany). The laser power was attenuated at the sample to approximately 5 mW to reduce photodegradation of heteroleptic iron(II) chromophores. RR signals were detected by an IsoPlane 160 spectrometer (Princeton Instruments, USA) with an entrance slit width of 50 µm and a grating with 1200 grooves/mm equipped with a thermoelectrically cooled CCD camera of 1340 x 100 pixels (PIXIS eXcelon, Princeton Instruments, USA). The spectral band of DMF at 1400 cm^{-1} was used as a reference for normalizing intensities and

wavenumbers. For spectral processing, rR spectra were background corrected, and the solvent spectrum was subtracted.

Nonresonant FT-Raman spectra (λ_{exc} = 1064 nm) of **P1** were recorded using a MultiRAM (Bruker) with a fiber-coupled diode pumped solid-state laser (DENICAFC LC-3/ 40, KLASTECH-Karpushko Laser Technologies). The laser power was set to 10 mW. For each spectrum 500 scans were averaged. A single IR 352 objective with a working distance of 16 mm focused the laser on the sample and collected the scattered light. The signal was detected by a nitrogen-cooled Ge-Diode (Bruker D418-T). The spectral resolution was 4 cm^{-1}.

2.8 Transient Absorption Spectroscopy

The ultrafast transient absorption (TA) experiments were conducted utilizing a custom-designed setup similar to the one described extensively elsewhere.[24,25] A Ti-Sapphire (Astrella, Coherant, USA) regenerative amplifier was employed, which generated ultrafast pulses centered at 800 nm, with a pulse width of ~85 fs and repetition rate of 1 kHz. The laser output (power- 5 mJ) was then divided using a beam splitter. The first part was focused into a rotating CaF_2 crystal to generate a broadband white light supercontinuum beam spanning 300 to 700 nm. Subsequently, this broadband pulse was split into two parts, one serving as the reference pulse and the other as the probe pulse. The second fraction of the amplifier output was directed towards an optical parametric amplifier (TOPAS prime, Light conversion, Lithuania) to generate pump pulses, which could be tuned across the UV-Vis-NIR spectral range. In this specific study, the pump pulses were centered at 470 nm. A mechanical chopper periodically blocked every alternate pump pulse, reducing the repetition rate to 500 Hz. A Berek compensator and a polarizer were utilized to set the relative polarization between the pump and probe pulses to the magic angle of 54.7°. The probe pulse was focused into a quartz cuvette with a path length of 1 mm by a concave mirror with a focal length of 500 mm. The spectra of the probe and reference pulses were acquired by a Czerny-Turner spectrograph (SP2150, Princeton Instruments) with a focal length of 150 mm, equipped with two CCD arrays (Pascher Instruments AB, Sweden). Due to the observation of significant coherent artifact signals,[26] a temporal pulse overlap range of ±150 fs was excluded from the data analysis procedure. The power of the pump pulse used ranged from 0.5 to 0.7 mW, and the optical density (OD) of the sample at the excitation wavelength ranged from 0.3 to 0.5. The fs-TA data was analyzed using the KIMOPACK tool.[27] Before conducting global lifetime analysis, the data was

numerically corrected for the chirp of the white-light probe. Global analysis was performed by using a model with 3 consecutive processes with errors obtained in a confidence level of 95%. Decay-associated spectra and kinetic fit traces are shown in Figures S 27 and S 28.

Using parameters obtained by global fitting, target analysis was performed for C1 and C2 using the following model:

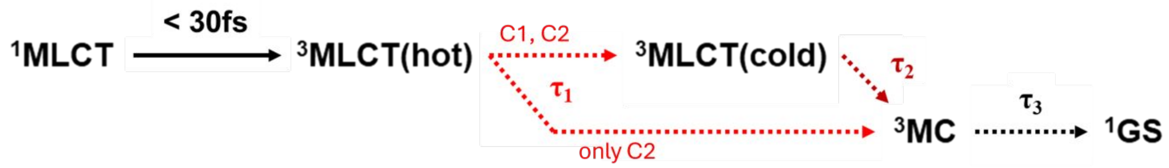

Figure S 1. "Hot branching" model used for target analysis

This model reflects the "hot-branching" model, where the initial populated ^{1}MLCT decays rapidly (below the temporal resolution of the experimental setup) via inter-system crossing to a vibronically excited ^{3}MLCT.[28] Following this, does this ^{3}MLCT(hot) state branch into a vibronically relaxed ^{3}MLCT(cold) via cooling and into a ^{3}MC state via internal conversion with a lifetime τ_1 and a branching ratio Φ, which denotes the fraction of molecules undergoing vibrational cooling into the ^{3}MLCT(cold). Finally does the ^{3}MLCT(cold) decay to a ^{3}MC with a lifetime τ_2, which further relaxes into the ^{1}GS via τ_3. The model is explained in greater detail in the main text.

A central parameter of this model is the branching ratio Φ. Therefore, the target analysis fit using the software KiMoPack was performed with different initialization values for Φ ranging from 0.2 to 0.8 and lifetime parameters were input as fixed values from the global analysis. The model input was as followed:

1. ^{3}MLCT(hot) $\xrightarrow{\tau_1}$ Φ ^{3}MLCT(cold) + (1-Φ) ^{3}MC
2. ^{3}MLCT(cold) $\xrightarrow{\tau_2}$ ^{3}MC
3. ^{3}MC $\xrightarrow{\tau_3}$ GS

Irrespective of the initialization value, for **C1** Φ converges to 1. This indicates the scenario in which photoexcited **C1** complexes mainly (within the signal-to-noise and the accuracy of the fit "exclusively") relax via initial cooling, i.e., hot ^{3}MLCT(hot) relaxes to ^{3}MLCT(cold), followed by internal conversion to metal-centered states ^{3}MC.

A different scenario emerges for **C2**, irrespective of the initialization value of Φ, when chosen between 0.2 and 0.8, the fit for Φ converges against a branching ratio very

close to the starting value. This indicates that within the signal-to-noise of the experiments a branching of roughly 50%.

Species-associated spectra are shown in Figure S 30. While spectra of the first and third component (τ_1 and τ_3) look rather similar when comparing **C1** and **C2** in various protonation steps, does the spectra associated with τ_2 look different for both molecules, indicating different processes happening.

We interpret this behavior as the ^{3}MLCT(hot)-^{3}MC deactivation channel being unfavored for **C1** compared to **C2**. We hypothesize that this roots in a stabilization of the ^{3}MC for **C1**, which can be seen by the longer ^{3}MC lifetimes (τ_3) of 60 to 70 ps for **C1** compared to 12 to 30 ps for **C2** based on energy-gap law. Stabilization of the ^{3}MC in turn lowers the overlap between the ^{3}MLCT and the ^{3}MC according to Marcus' theory, making the ^{3}MLCT(hot)-^{3}MC transition less likely for **C1** (see Figure S 2) for a graphical adaptation). Nonetheless, also other factors might contribute to this behavior, e.g., an altered coupling between the ^{3}MLCT and ^{3}MC in the two different isomers of the complex, caused by the C donor atoms of the NHC being situated in the equatorial position for **C2** as compared to one C atom being positioned in an equatorial position and the other one in an axial position for **C1**.

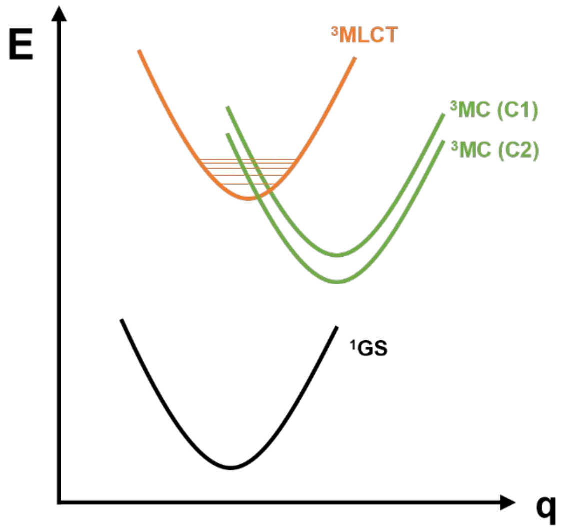

*Figure S 2. Simplified Jablonski diagram showing ^{3}MLCT, ^{1}GS and ^{3}MC of **C1** and **C2**. ^{3}MC energies are placed arbitrarily to demonstrate a higher overlap between ^{3}MC and ^{3}MLCT for **C2** compared to a lower-lying ^{3}MC of **C1**, thus making the ^{3}MLCT-^{3}MC more likely for **C2**.*

2.9 Stability and reactivity measurements:

UV-vis photostability measurements were either performed on a Horiba Duetta with EzSpec device placed in a nitrogen atmosphere glovebox (inert conditions) or on a Jasco V-670 UV-VIS-NIR Spectrometer (aerobic conditions). Photostability experiments were conducted in water-free, de-aerated ACN (inert). For aerobic conditions the solvent and stock solutions were taken out of the glovebox and the sample solutions were prepared under air. All samples had a concentration of 1×10^{-5} mol/l. The samples were irradiated using a 470 nm LED, P ca. 40-50 mW/cm^2 integrated into a custom reactor cooled by four fans, to control room temperature during irradiation. The inert samples were irradiated and measured in the glovebox. Optical quartz glass cuvettes with a pathlength of 10 mm were used.

NMR samples were placed in a modular 3D printed photoreactor and irradiated by a LST1_01F06_RYL1_000 LED (λ = 460 nm) with a current of 0.75 A and voltage of 3.5 V. The experiments were performed at room temperature *via* a ventilation system in the back of the reactor.

3. Synthesis

3.1 Synthesis of Ligand L1

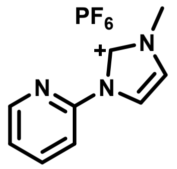

The ligand was synthesized by a slightly deviating literature procedure.[29] 2-bromopyridine 5.18 g (32.8 mmol) and 1-methylimidazole 2.78 g (33.9 mmol) were added to round bottom flask and were heated at 160°C for 24h. After cooling to room temperature 30 mL of an aqueous NH_4PF_6 (1 eq. NH_4PF_6) solution and 30 mL diethylether were added and the solution was placed overnight in the fridge. The resulting precipitate was filtered off, washed with water followed by diethylether. The crude product was recrystallized by acetonitrile/diethylether to get 2.7 g (8.84 mmol, 27%) of the product as brown crystals.

^{1}H NMR (400 MHz, CD_3CN) δ 9.24 (s, 1H), 8.59 (ddd, J = 4.8, 1.7, 0.7 Hz, 1H), 8.13 – 8.08 (m, 1H), 8.06 (t, J = 1.9 Hz, 1H), 7.72 (d, J = 8.2 Hz, 1H), 7.58 (ddd, J = 7.6, 4.8, 0.8 Hz, 1H), 7.55 (t, J = 1.9 Hz, 1H), 3.96 (s, 3H).

^{13}C NMR (400 MHz, CD_3CN) δ 150.51, 147.44, 141.50, 135.60, 126.39, 125.76, 120.25, 115.00, 37.49.

3.2 Synthesis of Complex P1

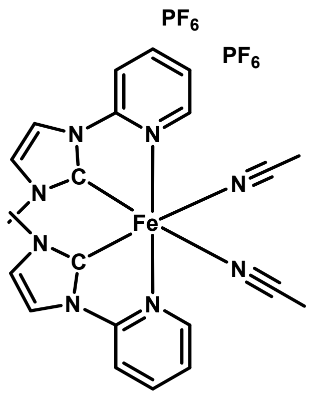

Under an argon filled glovebox atmosphere $Fe(HMDS)_2$ 209 mg (555 µmol) was dissolved in 5 mL of acetonitrile, then a solution of L1 338 mg (1.11 mmol) in 5 mL acetonitrile was added dropwise and the solution was stirred for 16 hours at room temperature. Purification is also performed under inert conditions; after evaporation of

the solvent with a high vacuum pump the crude product was recrystallized from acetonitrile/diethylether to get 300 mg (402 µmol, 72%) of the product as red crystals. Single crystal x-ray measurements show only one structure, where as in the ^{1}H-NMR two isomers can be observed. The ratio of the two isomers changes from a 6:1 to a final 3:2 ratio after approximately 5 hours.

P1 (C_2 symmetric isomer): ^{1}H NMR (400 MHz, CD_3CN) δ 8.76 (d, *J* = 5.5 Hz, 2H), 8.06 (t, *J* = 7.1 Hz, 2H, overlap with **P1**(C_1)), 8.00 (d, *J* = 2.0 Hz, 2H), 7.75 (d, *J* = 8.3 Hz, 2H, overlap with **P1**(C_1)), 7.36 (t, *J* = 6.4 Hz, 2H), 7.11 (d, *J* = 2.0 Hz, 2H), 2.67 (s, 6H), 1.96 (s, 6H, overlap with **P1**(C_1)).

P1 (C_1 symmetric isomer): ^{1}H NMR (400 MHz, CD_3CN) 9.30 (d, *J* = 4.8 Hz, 1H), 8.42 – 8.30 (m, 1H), 8.16 (d, *J* = 2.2 Hz, 1H), 8.10 (d, *J* = 2.2 Hz, 1H), 8.06 (t, *J* = 7.1 Hz, 1H, overlap with **P1**(C_2)), 7.82 – 7.77 (m, 1H), 7.75 (d, *J* = 8.3 Hz, 1H, overlap with **P1**(C_2)), 7.59 (d, *J* = 8.3 Hz, 1H), 7.53 (d, *J* = 2.2 Hz, 1H), 7.14 (d, *J* = 2.2 Hz, 1H), 6.85 (dd, *J* = 9.7, 3.5 Hz, 1H), 6.75 (d, *J* = 5.1 Hz, 1H), 4.03 (s, 3H), 3.06 (s, 3H), 1.96 (s, 6H, overlap with **P1**(C_2)).

P1 (C_1 symmetric isomer): ^{13}C NMR (400 MHz, CD_3CN) δ 199.24,156.48, 155.21, 140.71, 128.68, 122.88, 112.15, 35.68.

ESI-MS: *m/z* calculated for [M] = [$C_{22}H_{24}F_{12}FeN_8P_2$] = 746.0751

found: 601.1173 [M-PF_6^-]$^+$, 519.0604 [M-PF_6^--2ACN]$^+$, 560.0887 [M-PF_6^--ACN]$^+$

3.3 Synthesis of Complexes C1 and C2

Under argon atmosphere complex **P1** 125 mg (0.17 mmol) and bibenzimidazole 41 mg (0.17 mmol) were dissolved in 10 mL DMF and stirred for 16 hours. After completion of the reaction, the solvent was evaporated to get the crude product as a red powder (150 mg). The work up and crystallization is also performed in an argon filled glovebox.

First a recrystallization from a mixture of MeOH/ACN 2:1 and diethylether was performed, the resulting crystals were filtered off and washed with diethylether.

3.3.1 Isolation of C2

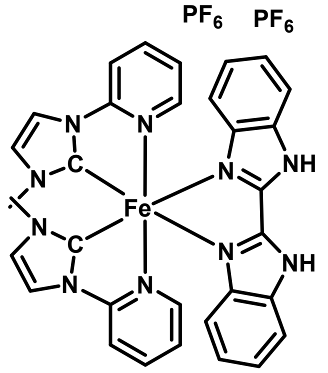

The crystals (mainly **C2** with some leftover **C1**) were recrystallized by diffusion crystallization from ACN/MeOH 1:1 and diethylether, the resulting crystals were filtered off and washed with diethylether. This procedure was repeated one more time to get 50 mg (0.56 mmol, 34%) of pure isomer **C2**.

3.3.2 Isolation of C1

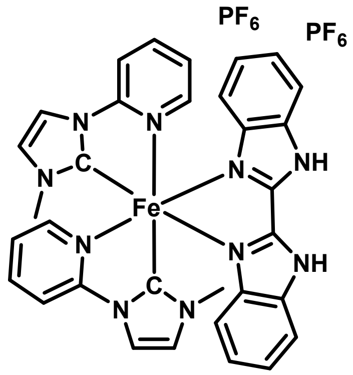

To the mother liquor of the first crystallization diethylether was added and the solution was left standing for 2 weeks, whereas 22 mg (0.026 mmol, 16%) of red crystals mostly containing **C1** could be obtained (**C1**/**C2** ratio 25:1).

In later batches C1 could also be obtained by a second diffusion crystallization of the mother liquor with diethylether, after 3-4 days crystals of C1 could be obtained in similar purity and yields.

C2: ^{1}H NMR (400 MHz, CD_3CN) δ 8.21 (d, *J* = 2.1 Hz, 2H), 7.86 (d, *J* = 8.3 Hz, 2H), 7.73 (t, *J* = 7.3 Hz, 2H), 7.62 (d, *J* = 8.2 Hz, 2H), 7.50 (t, *J* = 7.7 Hz, 2H), 7.43 (d, *J* = 1.9 Hz, 2H), 7.30 (t, *J* = 7.8 Hz, 2H), 7.04 (d, *J* = 5.5 Hz, 2H), 6.79 (t, *J* = 6.2 Hz, 2H), 6.24 (d, *J* = 8.4 Hz, 2H), 2.82 (s, 6H).

^{1}H NMR (400 MHz, DMSO) δ 14.17 (s, 2H), 8.75 (d, *J* = 2.2 Hz, 2H), 8.00 (d, *J* = 8.2 Hz, 2H), 7.95 (d, *J* = 8.4 Hz, 2H), 7.88 – 7.83 (m, 2H), 7.81 (d, *J* = 2.2 Hz, 2H), 7.46 (t,

J = 7.7 Hz, 2H), 7.28 (t, *J* = 7.7 Hz, 2H), 7.02 (d, *J* = 5.2 Hz, 2H), 6.97 – 6.90 (m, 2H), 6.11 (d, *J* = 8.3 Hz, 2H), 2.82 (s, 6H)

^{13}C NMR (400 MHz, CD_3CN) δ 204.52, 156.20, 154.11, 146.07, 143.65, 139.56, 137.20, 129.16, 126.93, 126.12, 122.83, 118.47, 116.53, 115.42, 111.45, 36.03.

C1: ^1H NMR (400 MHz, CD_3CN) δ 8.32 (d, *J* = 2.3 Hz, 1H), 8.06 (d, *J* = 2.2 Hz, 1H), 8.03 – 7.95 (m, 2H), 7.91 (d, *J* = 8.2 Hz, 1H), 7.77 (d, *J* = 8.2 Hz, 1H), 7.71 (d, *J* = 8.2 Hz, 1H), 7.51 (t, *J* = 7.9 Hz, 2H), 7.47 (d, *J* = 2.3 Hz, 1H), 7.31 (t, *J* = 7.6 Hz, 1H), 7.26 – 7.18 (m, 2H), 7.13 – 7.07 (m, 3H), 7.04 (ddd, *J* = 7.2, 5.8, 1.2 Hz, 1H), 6.98 – 6.93 (m, 1H), 5.81 (d, *J* = 8.2 Hz, 1H), 5.26 (d, *J* = 8.3 Hz, 1H), 3.23 (s, 3H), 2.67 (s, 3H).

C1(later batch) ^1H NMR (400 MHz, CD_3CN): δ 11.86 (s, 1H), 11.67 (s, 1H) 8.32 (d, *J* = 2.2 Hz, 1H), 8.08 (d, *J* = 2.2 Hz, 1H), 8.05 – 7.99 (m, 2H), 7.90 (t, *J* = 7.72 Hz, 2H), 7.80 (d, *J* = 8.3 Hz, 1H), 7.68 (d, *J* = 8.3 Hz, 1H), 7.51 – 7.43 (m, 3H), 7.38 (t, *J* = 7.5 Hz, 1H), 7.22 – 7.13 (m, 4H), 7.09 – 7.02 (m, 2H), 5.84 (d, *J* = 8.4 Hz, 1H), 5.27 (d, *J* = 8.4 Hz, 1H), 3.22 (s, 3H), 2.70 (s, 3H).

^1H NMR (400 MHz, DMSO) δ 14.03 (s, 2H), 8.88 (d, *J* = 1.9 Hz, 1H), 8.61 (d, *J* = 1.8 Hz, 1H), 8.30 (d, *J* = 8.3 Hz, 1H), 8.19 – 8.10 (m, 3H), 7.97 (d, *J* = 8.0 Hz, 1H), 7.85 (d, *J* = 1.9 Hz, 1H), 7.75 (d, *J* = 7.6 Hz, 1H), 7.51 (d, *J* = 1.9 Hz, 1H), 7.42 (d, *J* = 4.8 Hz, 2H), 7.36 – 7.29 (m, 2H), 7.22 – 7.12 (m, 3H), 7.03 (t, *J* = 7.8 Hz, 1H), 5.71 (d, *J* = 8.4 Hz, 1H), 5.16 (d, *J* = 8.4 Hz, 1H), 3.21 (s, 3H), 2.70 (s, 3H)

^{13}C NMR (400 MHz, CD_3CN) δ 212.06, 208.67, 157.41, 155.73, 154.38, 154.04, 152.62, 148.64, 145.53, 144.49, 140.52, 140.23, 139.57, 129.22, 128.78, 125.21, 124.90, 124.62, 124.59, 123.93, 122.54, 119.33, 117.92, 116.43, 115.92, 115.85, 115.36, 111.86, 111.75, 45.57, 36.88, 35.74.

HR-MALDI-MS: *m/z* calculated for [M] = [$C_{32}H_{28}F_{12}FeN_{10}P_2$] = 898.1125 found: *m/z* = 607.1765 ([M-2PF_6^--H^+]$^+$); m/z = 608.1803 ([M-2PF_6^-]$^{2+}$)

3.3.3 Preparation of deprotonated complexes for cyclic voltammetry

To a solution of protonated complex **C1^{prot}** or **C2^{prot}** (~30-50 mg) in dry acetonitrile (5 ml) prepared under a protective N_2 atmosphere were added five drops of 1,8-Diazabicyclo[5.4.0]undec-7-ene. A darkening of the red solution towards dark reddish-purple was observed. The solution was briefly shaken and subsequently left

standing at room temperature. Within an hour, a deep-purple solid had precipitated which was collected on a glass frit (Por. 4) and subsequently washed with dry ACN, THF, and Et_2O. The respective product was dried *in vacuo* and used for cyclic voltammetry without further purification. Purity and composition were confirmed by ^{1}H-NMR spectroscopy in deuterated DMSO.

^{1}H NMR (**C1**$^{\mathbf{deprot}}$, 400 MHz, DMSO-d$_6$): δ 8.74 (d, *J* = 2.2 Hz, 1H), 8.44 (d, *J* = 2.2 Hz, 1H), 8.14 (d, *J* = 8.2 Hz, 1H), 8.04 – 7.90 (m, 3H), 7.72 (d, *J* = 2.2 Hz, 1H), 7.48 (d, *J* = 7.9 Hz, 1H), 7.29 (d, *J* = 2.1 Hz, 1H), 7.23 (t, *J* = 6.1 Hz, 2H), 7.12 (dd, *J* = 9.4, 5.9 Hz, 2H), 7.07 (td, *J* = 5.8, 2.7 Hz, 1H), 6.75 (t, *J* = 7.5 Hz, 1H), 6.66 (t, *J* = 7.5 Hz, 1H), 6.54 (t, *J* = 7.1 Hz, 1H), 6.38 (t, *J* = 7.1 Hz, 1H), 5.48 (d, *J* = 8.0 Hz, 1H), 4.96 (d, *J* = 8.0 Hz, 1H), 3.16 (s, 3H), 2.50 (s, 3H overlap with DMSO peak)

^{1}H NMR (**C2**$^{\mathbf{deprot}}$, 400 MHz, DMSO-d$_6$): δ 8.63 (d, *J* = 2.2 Hz, 2H), 7.83 (d, *J* = 8.2 Hz, 2H), 7.70 – 7.60 (m, 4H), 7.47 (d, *J* = 7.8 Hz, 2H), 6.84 – 6.74 (m, 6H), 6.66 (t, *J* = 7.1 Hz, 2H), 5.91 (d, *J* = 7.9 Hz, 2H), 2.76 (s, 6H)

3.3.4 Preparation of single crystals of C2$^{\mathbf{prot}}$-TFA

A small sample of **C2**$^{\mathbf{deprot}}$ (~5 mg) prepared as described in 3.3.3 was suspended in acetone (0.5 ml) and pure TFA was added *via* a capillary. Upon mixing, the dark purple suspension quickly turned into bright-red solution. The solution was subjected to vapor diffusion of ether for crystallization which was unsuccessful. After removal of all solvents, the residue was again dissolved in 0.1 ml of acetonitrile and layered with ether *via* vapor diffusion. Single-crystals of **C2**$^{\mathbf{prot}}$ TFA salt were harvested after two days. To confirm the integrity of **C2**$^{\mathbf{prot}}$ after treatment with TFA, protonation was also carried out in DMF solution (10^{-5} M) of **C2**$^{\mathbf{deprot}}$. The respective spectrum shows the recovery of the absorption features characteristic for **C2**$^{\mathbf{prot}}$. In addition, an ^{1}H-NMR spectrum of residual crystals was also recorded in acetonitrile, showing the expected resonances. The corresponding ^{19}F-NMR spectrum confirms the presence of trifluoroacetate as indicated by a resonance at -75.55 ppm, as well as the absence of PF_6.

^{1}H NMR (400 MHz, CD_3CN) δ 16.27 (s, 2H), 8.21 (d, *J* = 2.2 Hz, 2H), 7.78 – 7.68 (m, 4H), 7.61 (d, *J* = 8.3 Hz, 2H), 7.43 (dd, *J* = 9.9, 5.0 Hz, 4H), 7.25 (t, *J* = 7.8 Hz, 2H),

7.08 (d, J = 5.2 Hz, 2H), 6.79 (dd, J = 9.6, 3.6 Hz, 2H), 6.24 (d, J = 8.4 Hz, 2H), 2.83 (s, 6H).

^{19}F NMR (400 MHz, CD_3CN) δ -75.55 (s)

3.4 Preparation of $[(18\text{-crown-}6)K]_2S_2O_8$

540 mg of potassium peroxodisulfate (2 mmol) were suspended in acetonitrile (10 ml). A solution of 18-crown-6 (600 mg, 2.27 mmol) in acetonitrile (10 ml) was added under stirring. After stirring for 5 minutes, the remaining suspension was filtered through glass wool. 200 ml of ethyl acetate were poured into the filtrate and the mixture slowly turned cloudy. The mixture was left standing for one day at room temperature. Colorless needle-like crystals were collected by filtration, and the crystals thoroughly washed with ethyl acetate and diethylether. The solid white crystalline product was finally dried *in vacuo*. Yield: 667 mg (0.83 mmol, 73% in relation to 18-crown-6). The composition of the product was confirmed by scXRD analysis (see Figure S 72, Table S 41).

^{1}H NMR (600 MHz, DMSO-d_6) δ 3.55 (s, 24H)

^{13}C NMR (600 MHz, DMSO-d_6) δ 69.4

4. NMR Spectroscopy

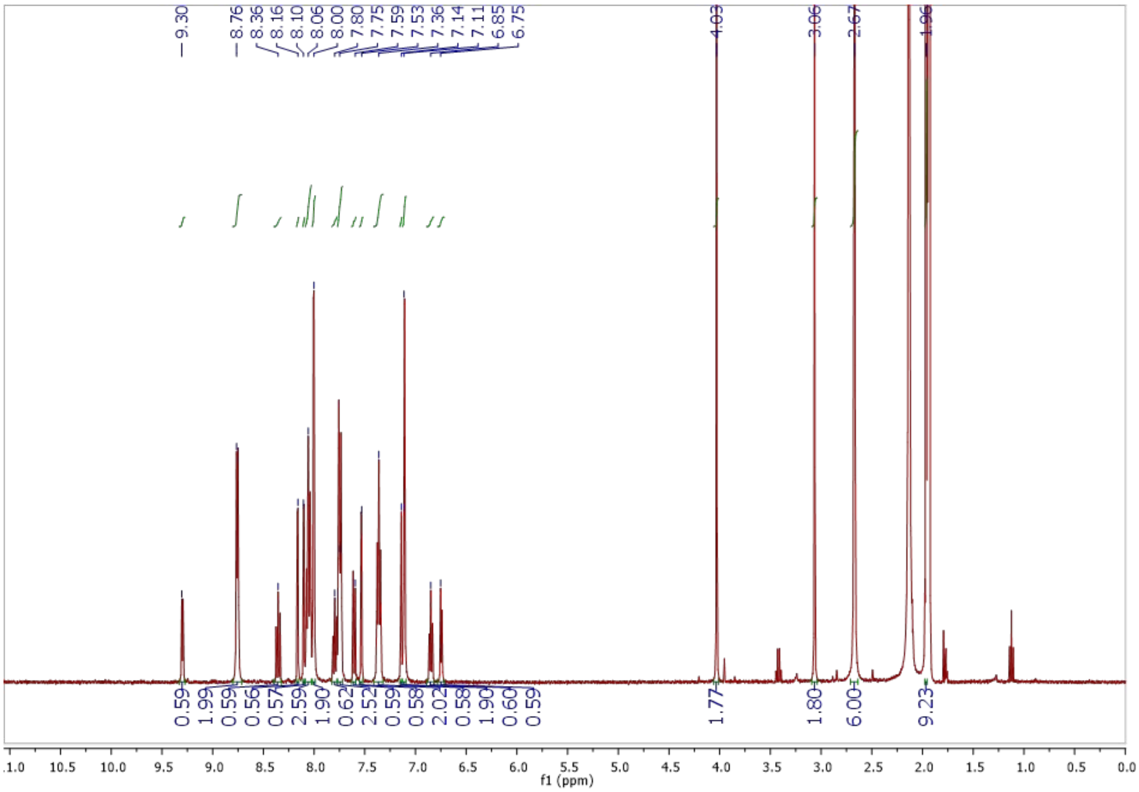

*Figure S 3: ^{1}H-NMR of **P1** in ACN-d_3.*

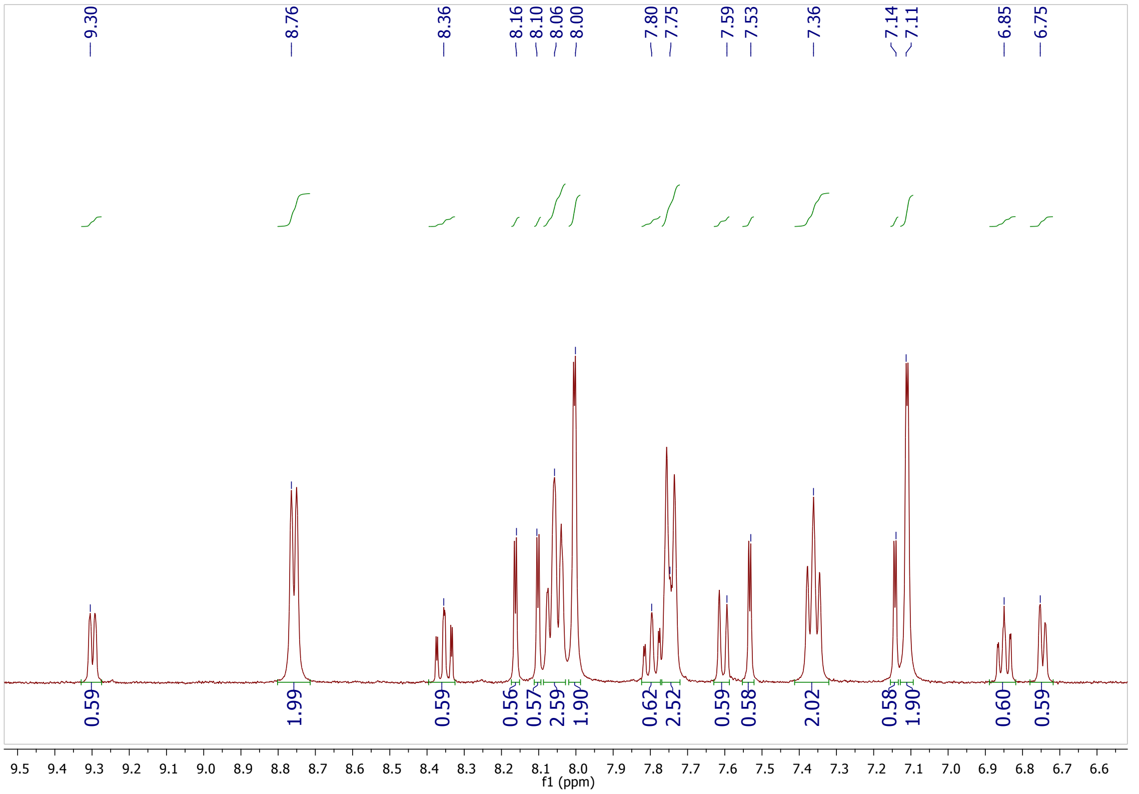

*Figure S 4: ^{1}H-NMR of **P1** in ACN-d_3 (aromatic region)*

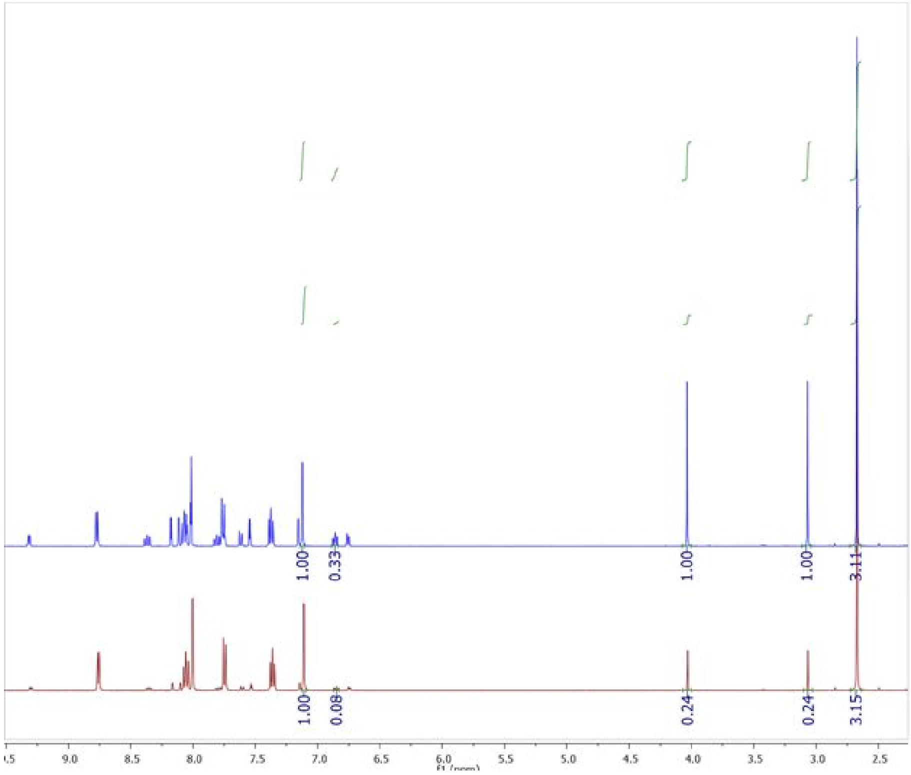

*Figure S 5: Comparison ^{1}H-NMR of **P1** in ACN-d_3*

red: direct measurement after dissolving of the crystals; blue: after 24 hours.

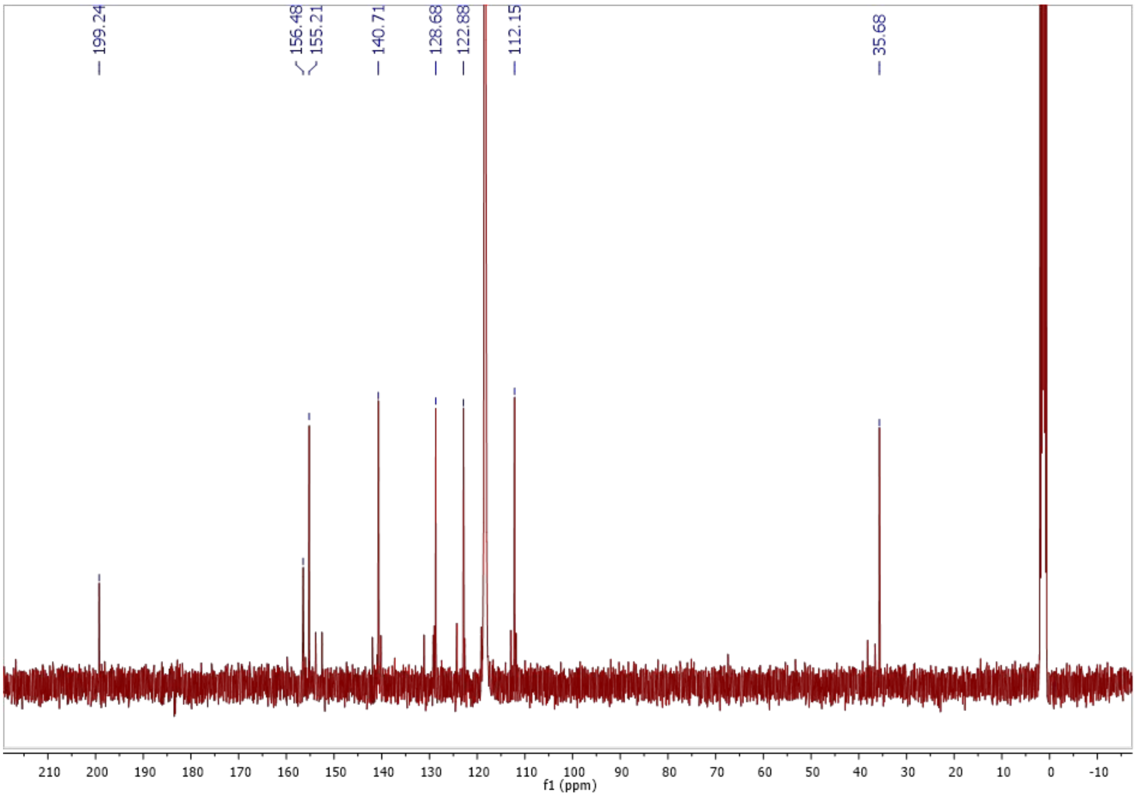

*Figure S 6: ^{13}C-NMR of **P1** in ACN-d_3.*

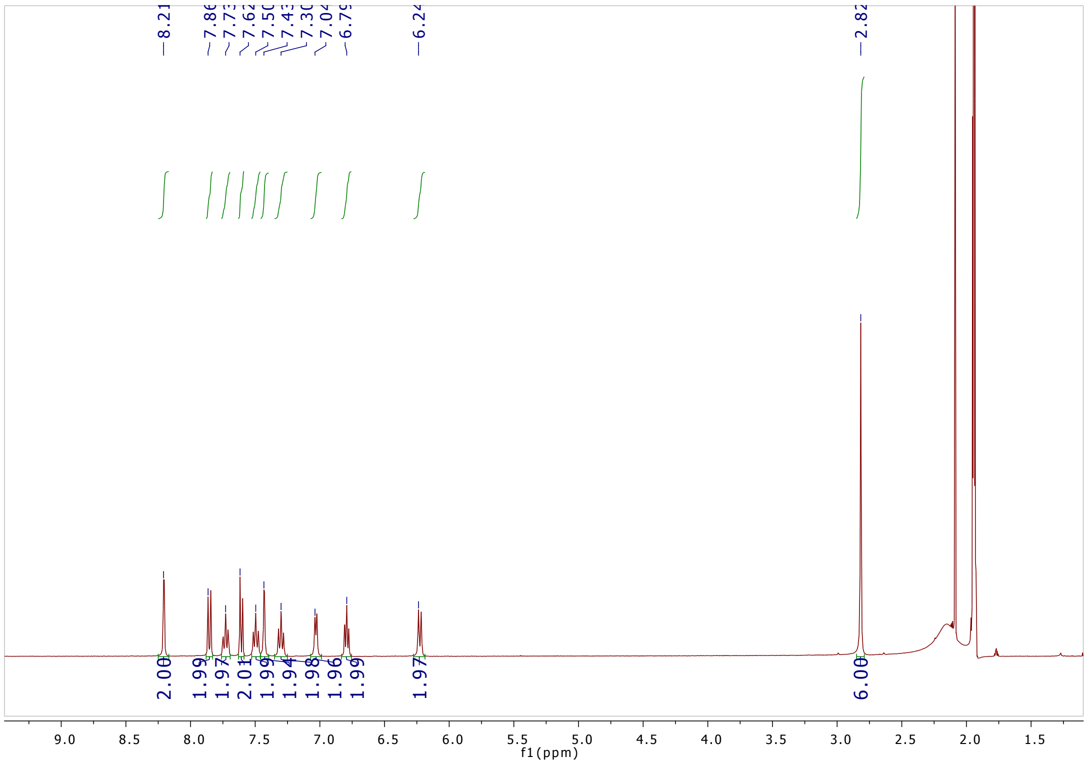

*Figure S 7: ^{1}H-NMR of **C2** in ACN-d_3.*

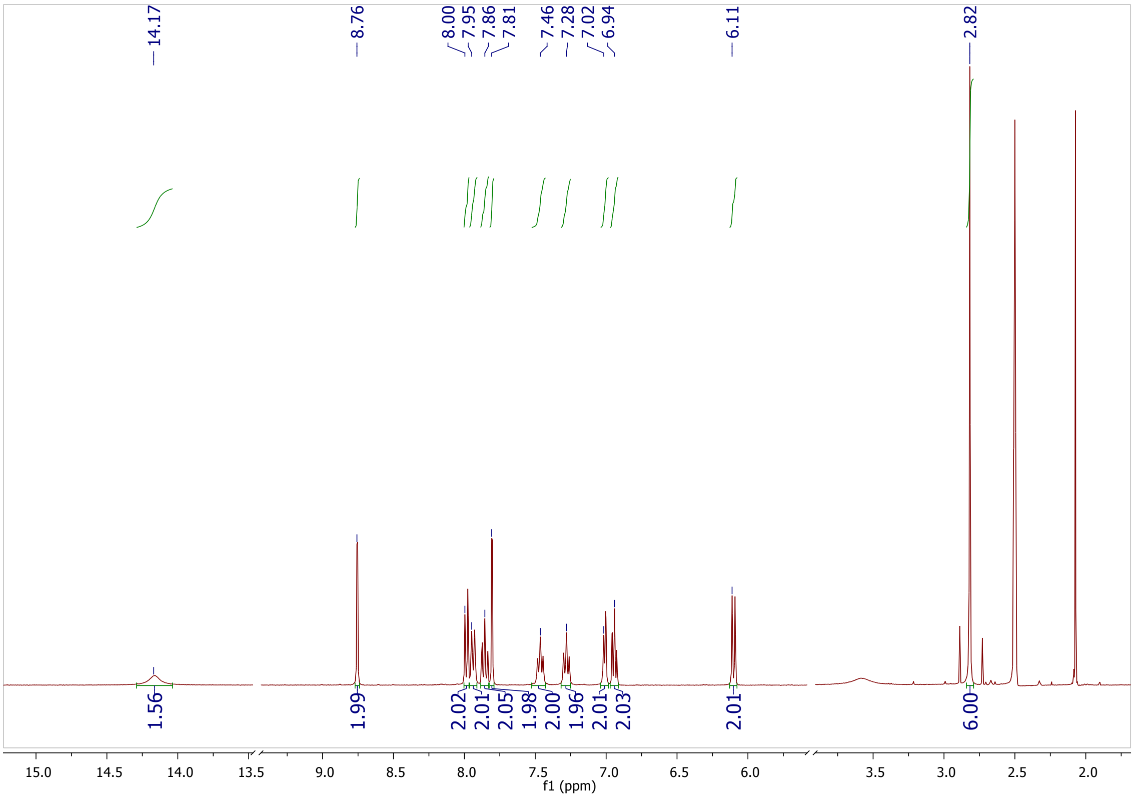

*Figure S 8: ^{1}H-NMR of **C2** in DMSO-d_6.*

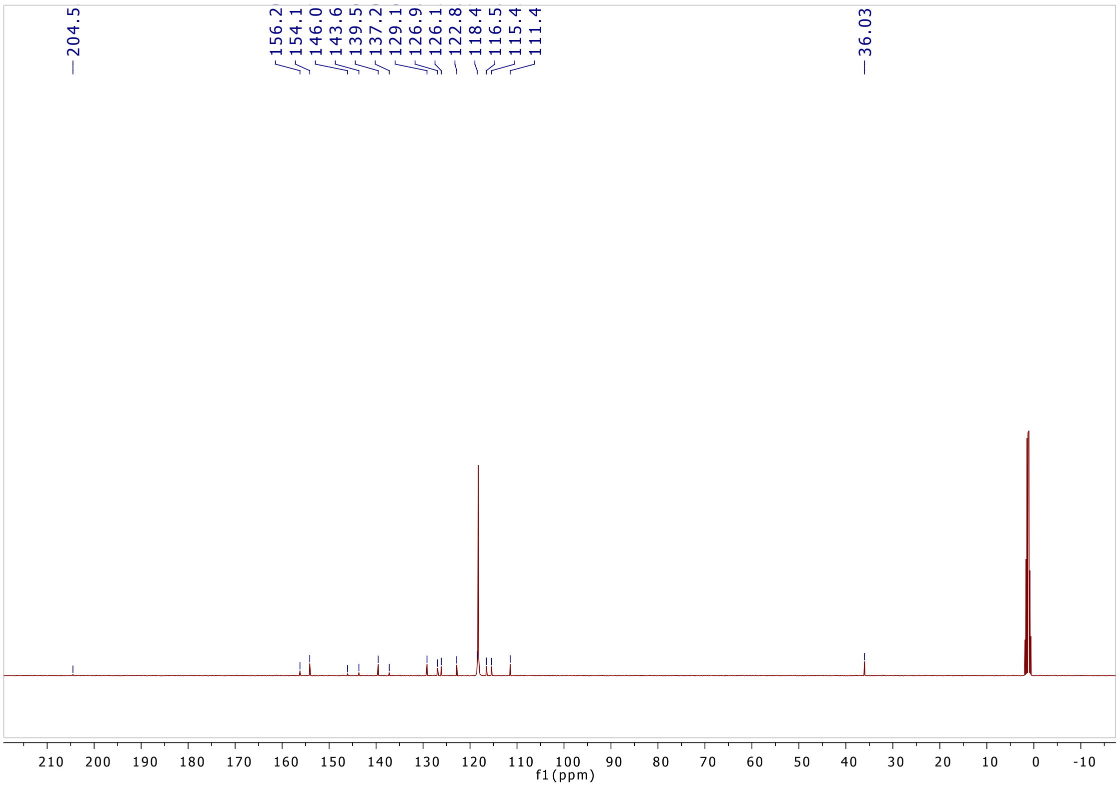

*Figure S 9: ^{13}C-NMR of **C2** in ACN-d_3.*

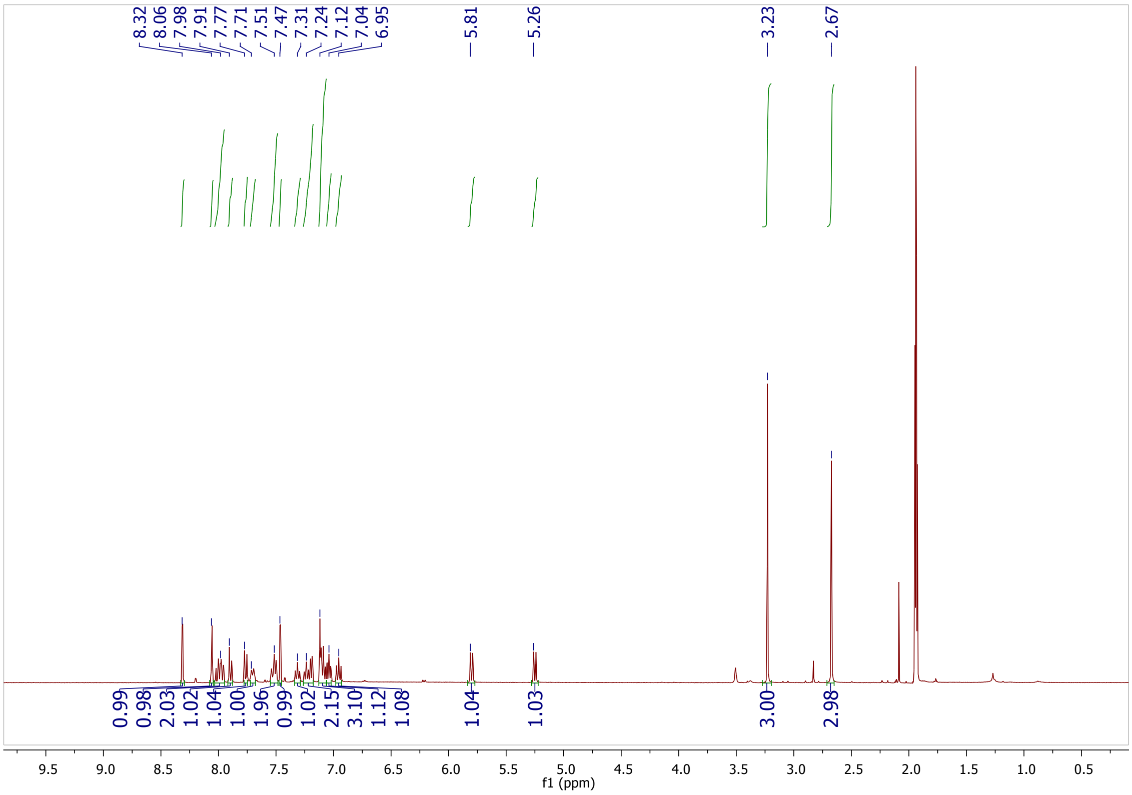

*Figure S 10: ^{1}H-NMR of **C1** in ACN-d_3 obtained through letting the mother liquor stand for 2 weeks.*

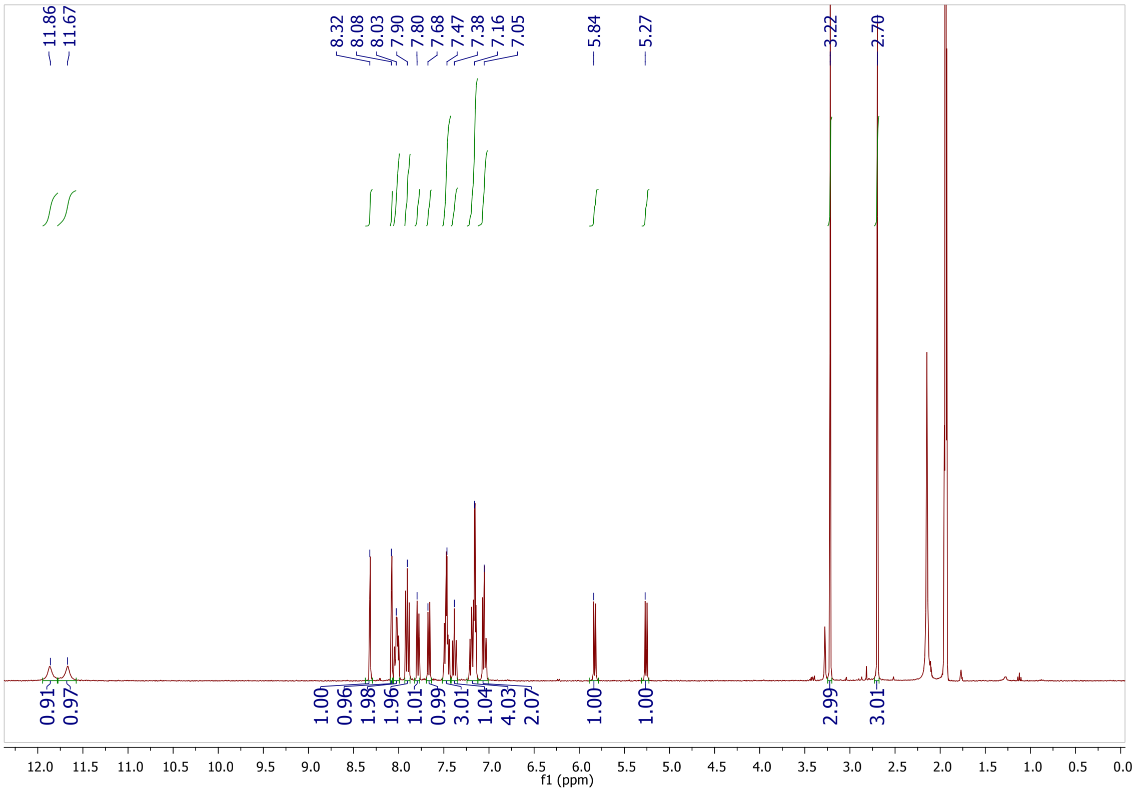

Figure S 11: ^{1}H-NMR of **C1** in ACN-d_3 obtained by a second diffusion crystallization of the mother liquor.

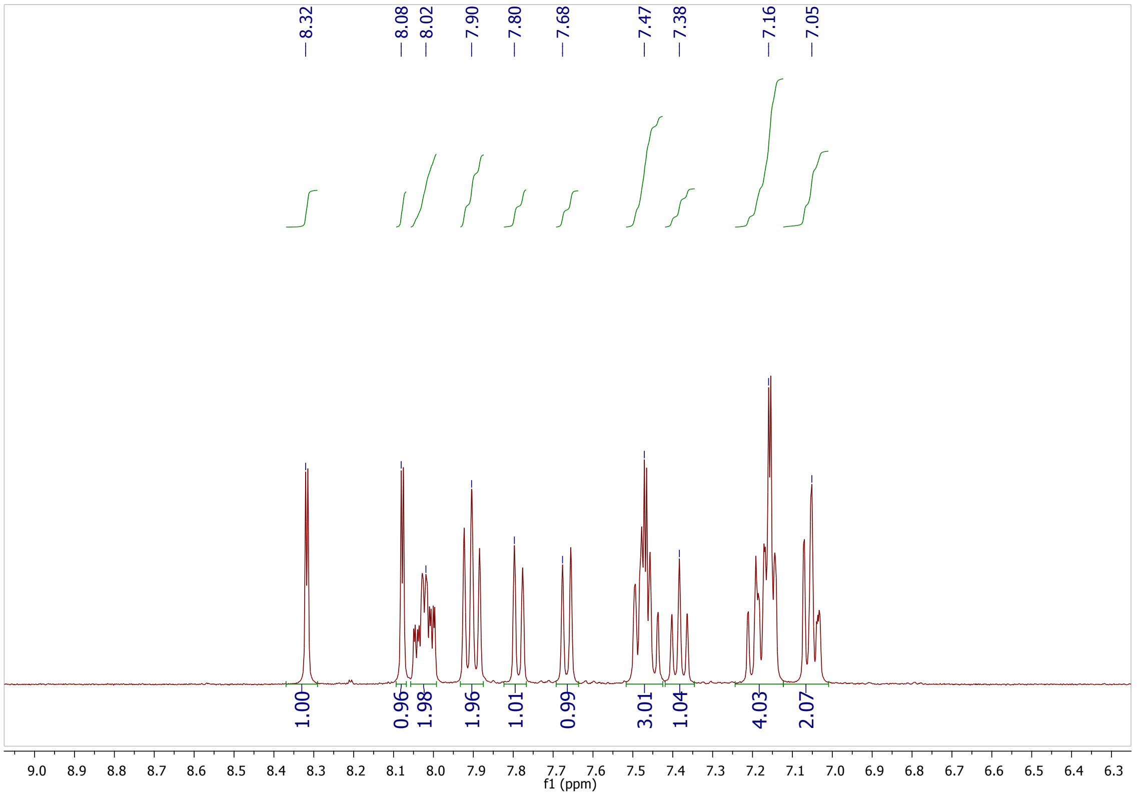

Figure S 12: ^{1}H-NMR of **C1** in ACN-d_3 (aromatic region)

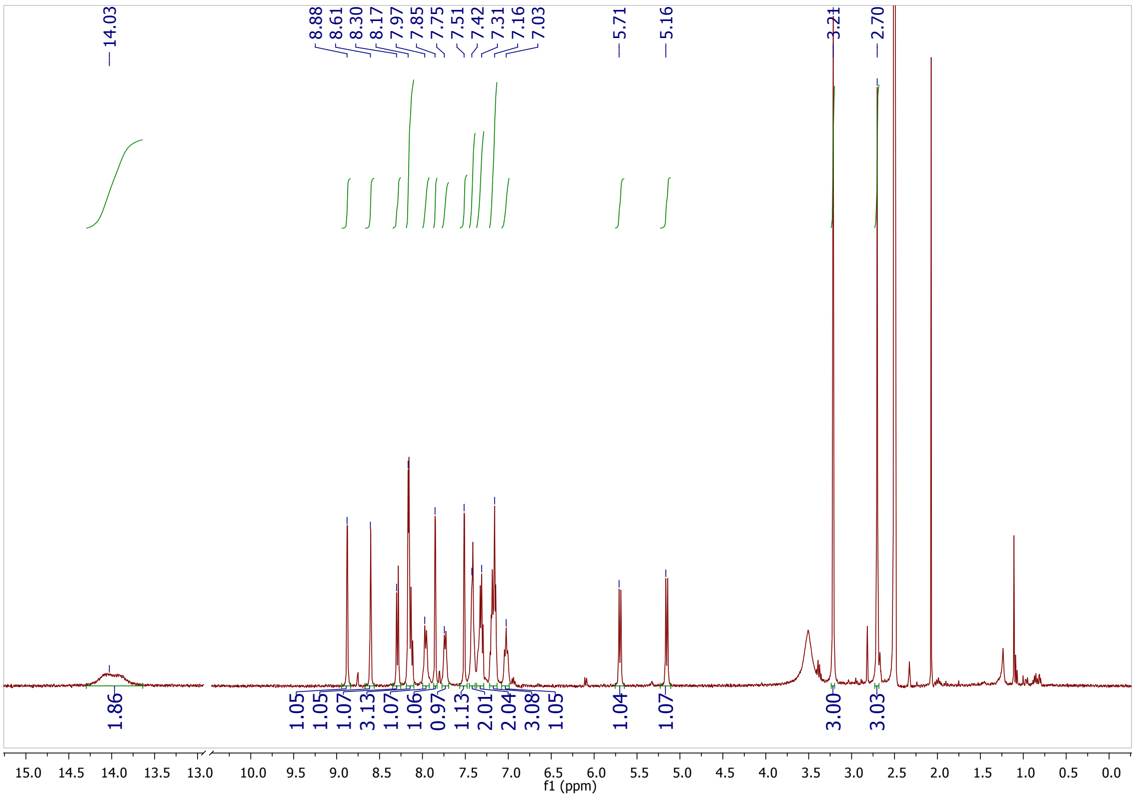

*Figure S 13: ^{1}H-NMR of **C1** in DMSO-d_6.*

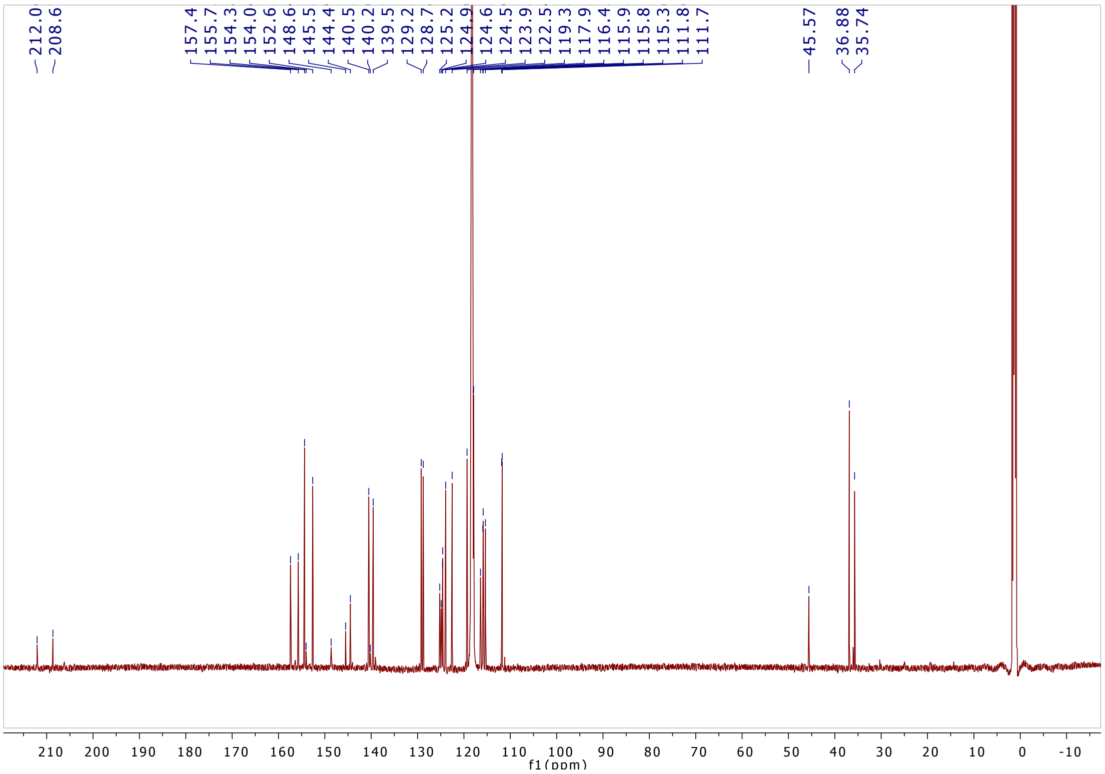

*Figure S 14: ^{13}C-NMR of **C1** in ACN-d_3.*

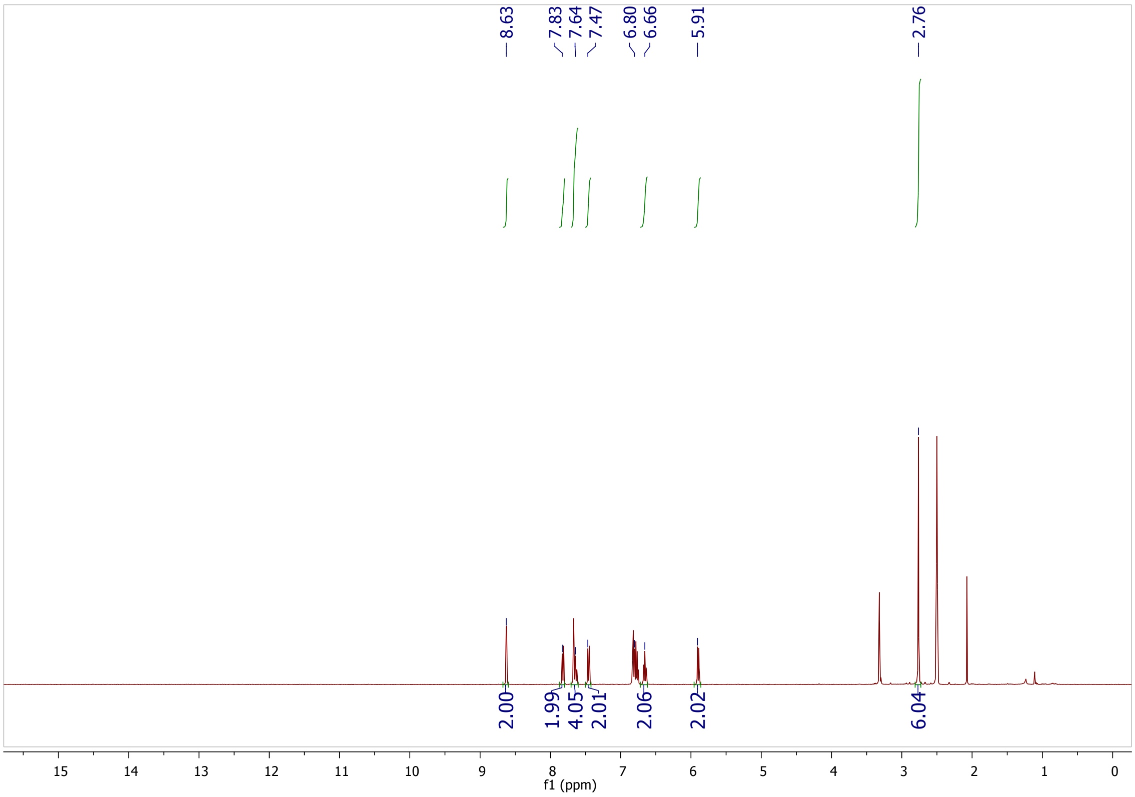

*Figure S 15: ^{1}H NMR of deprot. **C2** in DMSO-d_6*

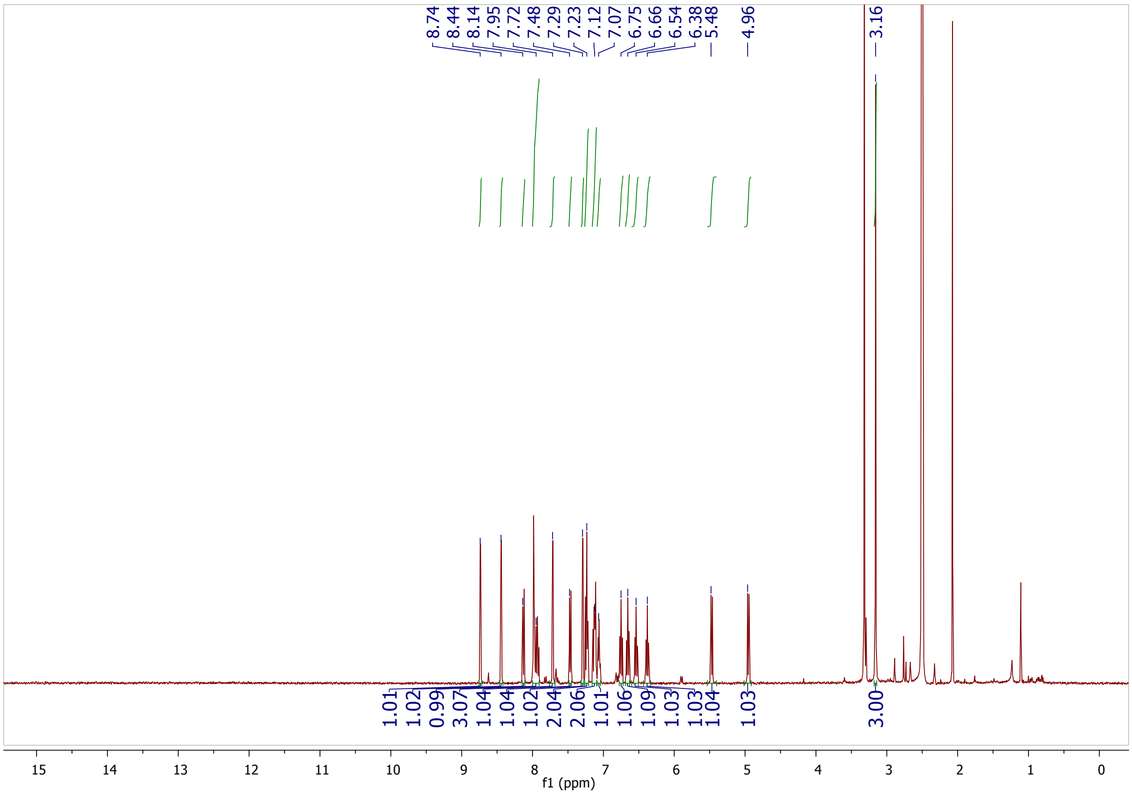

Figure S 16: ^{1}H-NMR of deprot. ***C1*** *in DMSO-d$_6$.*

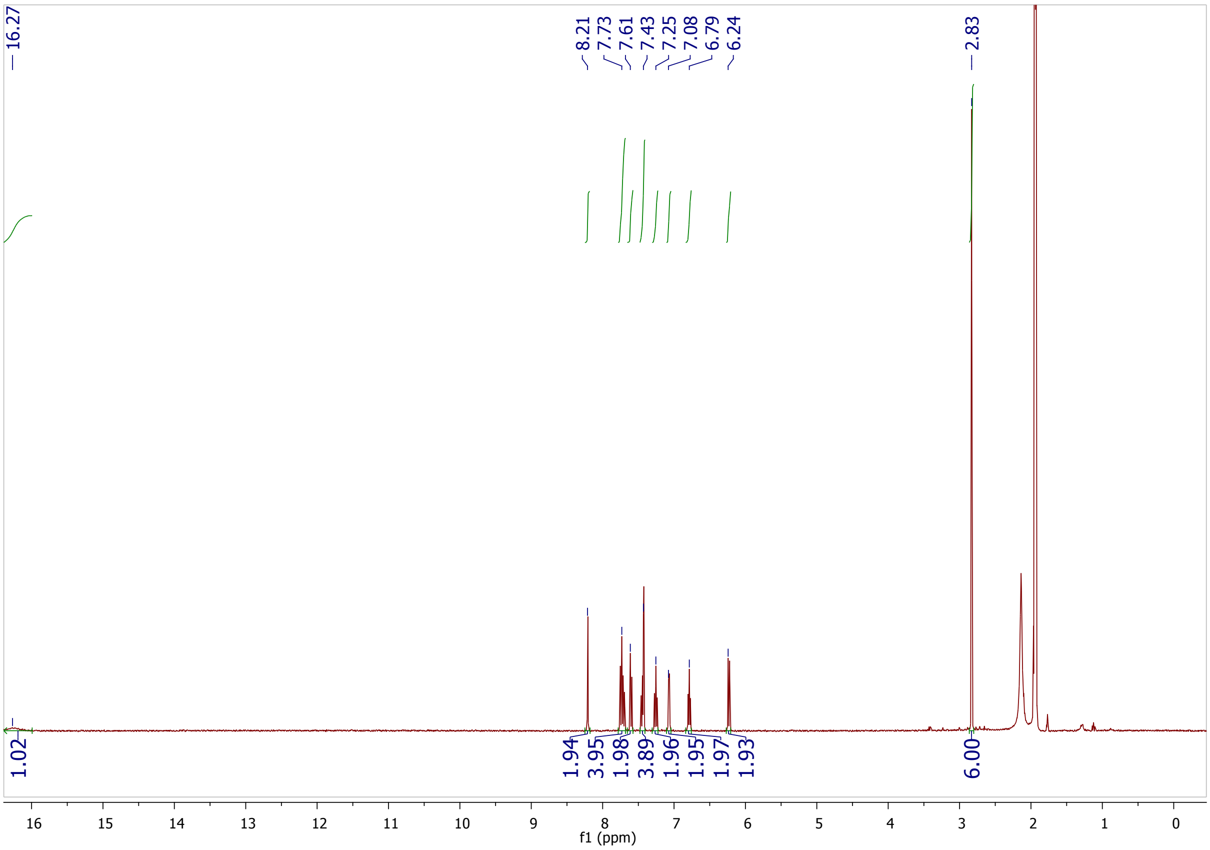

*Figure S 17: ^{1}H-NMR of deprot.****C2 + TFA*** *in ACN-d$_3$.*

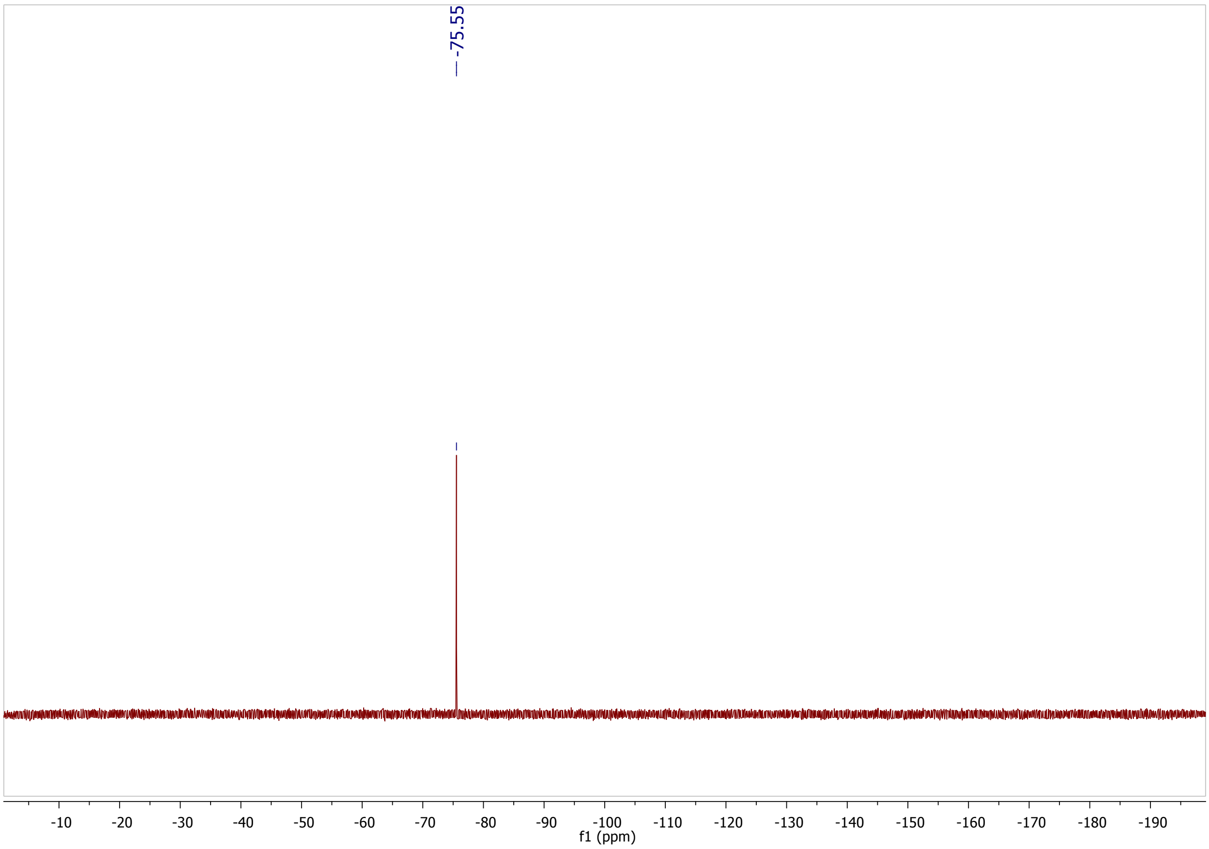

*Figure S 18: ^{19}F NMR of deprot.**C2 + TFA** in ACN-d_3.*

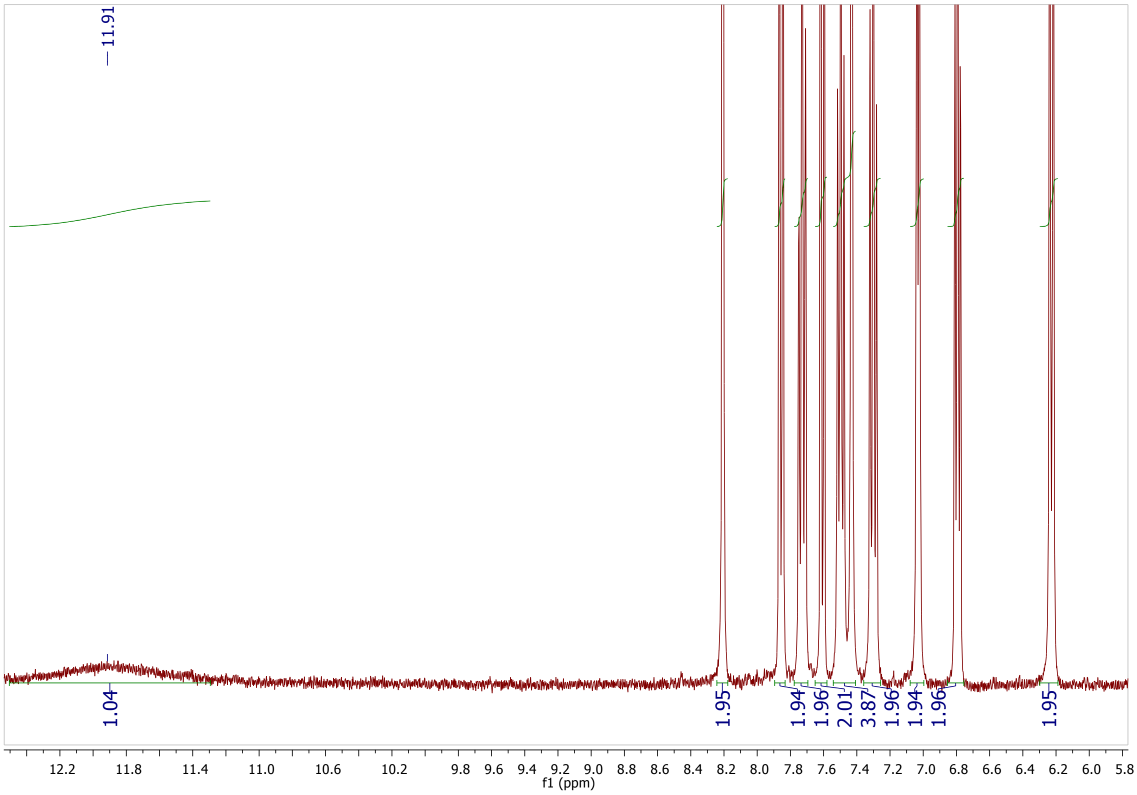

*Figure S 19: ^{1}H-NMR of **C2** in ACN-d_3; aromatic region to show the bibenzimidazole N-H peak.*

5. Mass Spectrometry

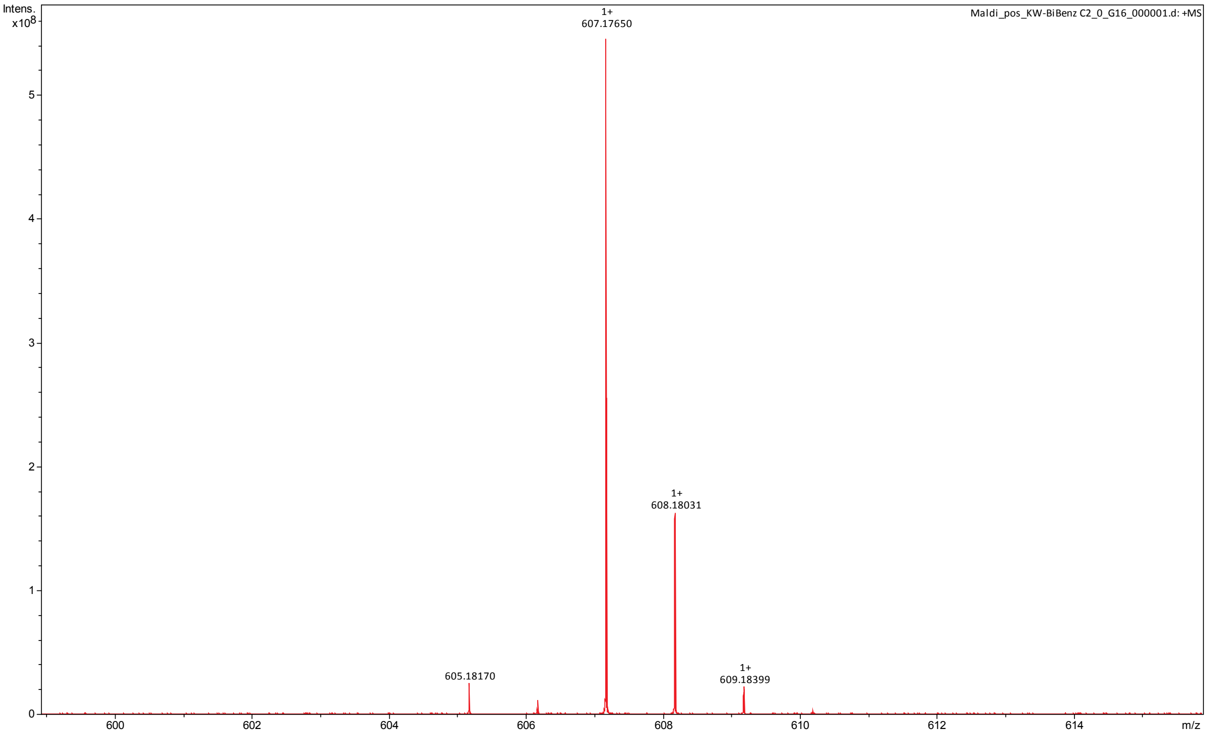

*Figure S 20: MALDI MS of **C2**.*

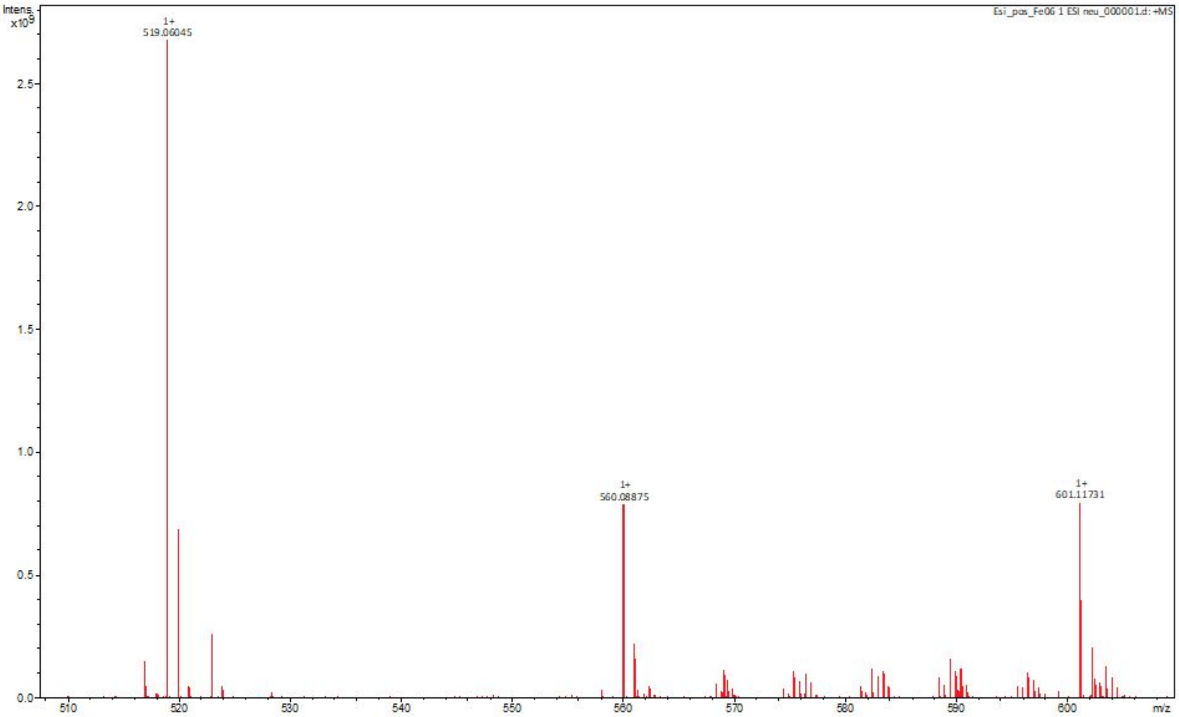

*Figure S 21: HR-ESI-MS of **P1**.*

6. Steady-state UV/Vis Spectroscopy

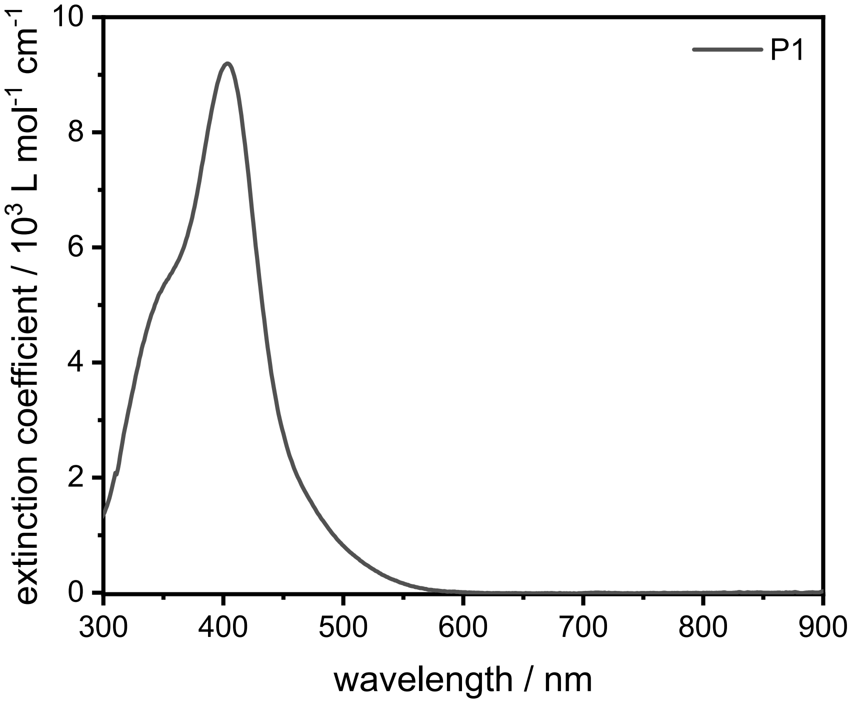

*Figure S 22: Extinction coefficient of complex **P1** in ACN.*

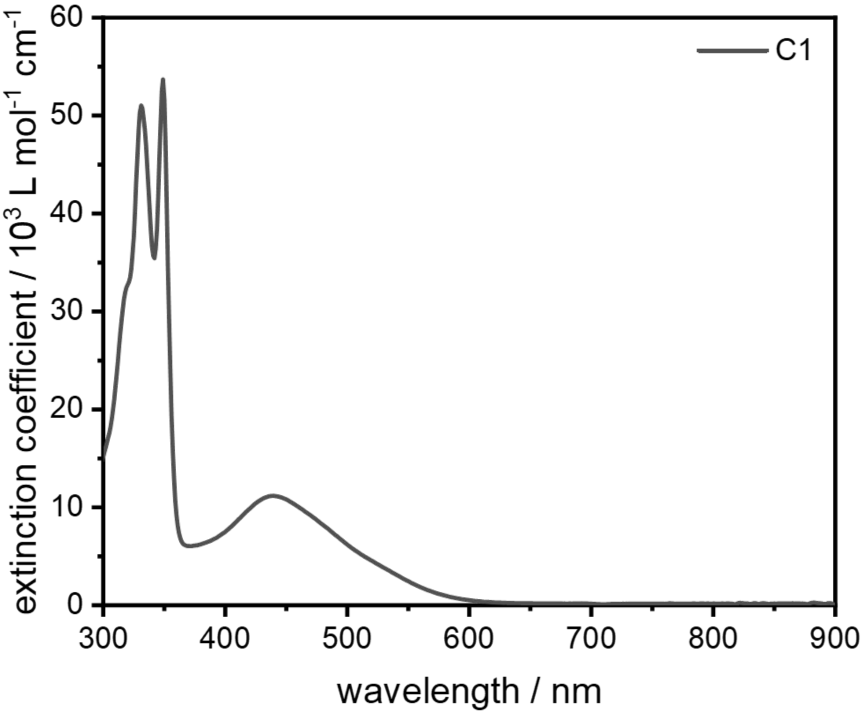

*Figure S 23: Extinction coefficient of complex **C1** in ACN.*

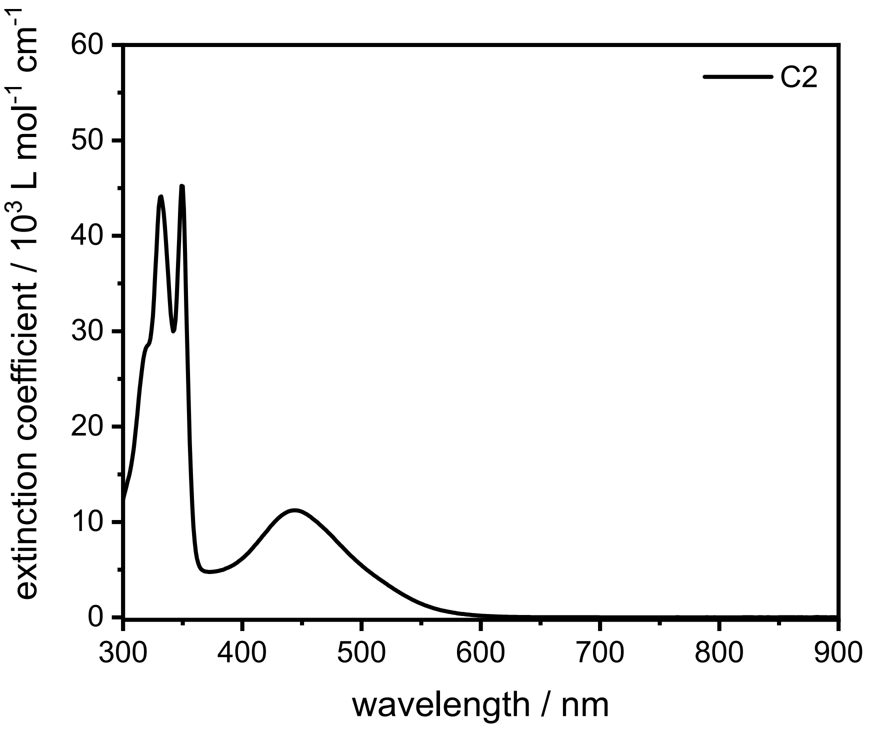

*Figure S 24: Extinction coefficient of complex **C2** in ACN.*

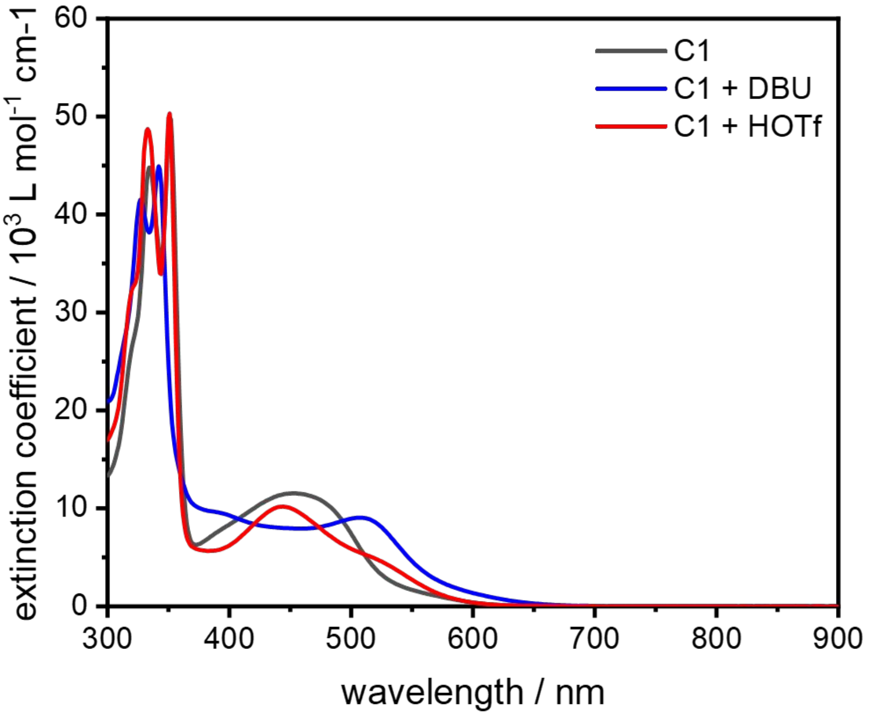

*Figure S 25: Extinction coefficient of complex **C1** in DMF.*

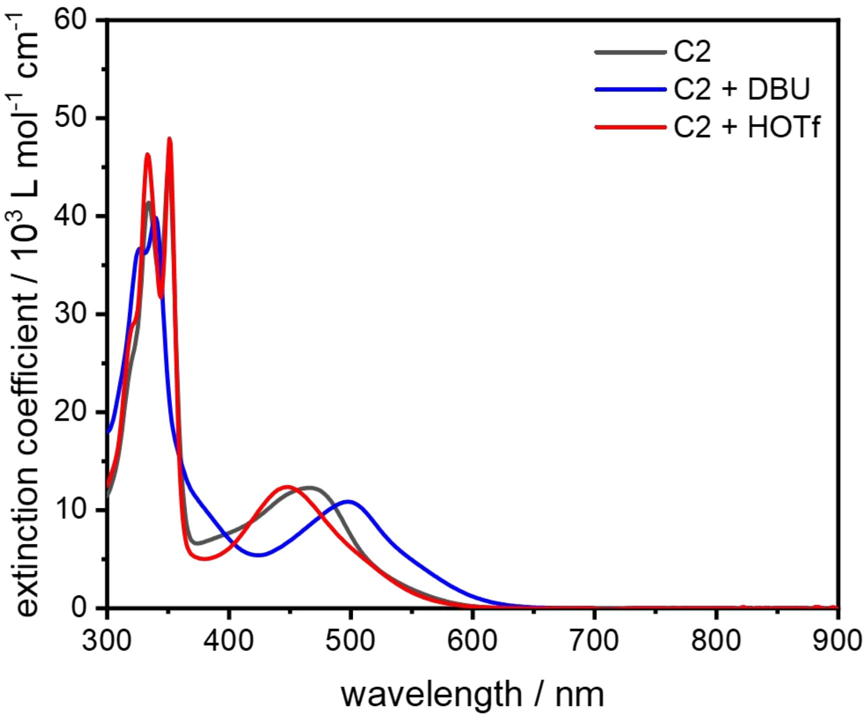

*Figure S 26: Extinction coefficient of complex **C2** in DMF.*

*Table S 1: Absorption maxima of **C1** and **C2** in ACN.*

Complex in ACN	$\lambda_{max,abs}$ / nm (ε / 10^3 L mol^{-1} cm^{-1})
**P1**	403(9.2)
**C1**	331(51.0), 349(53.7), 439(11.2)
**C2**	332(44.1), 349(45.2), 444(11.2)

Table S 2: Absorption maxima of the complexes in dependency of their protonation state in DMF.

Complex in DMF	$\lambda_{max,abs}$ / nm (ε / 10^3 L mol^{-1} cm^{-1})
**C1**	335 (44.8) 352 (49.7) 453 (11.5)
**C1**+DBU	327 (41.5) 342 (44.9) 506 (9.1)
**C1**+HOTf	333 (48.7) 351 (50.3) 444 (10.2)
**C2**	334 (41.4) 351 (45.3) 465 (12.3)
**C2**+DBU	327 (36.7) 339 (39.8) 497 (10.9)
**C2**+HOTf	333 (46.3) 351 (47.9) 447 (12.4)

7. Transient Absorption Spectroscopy

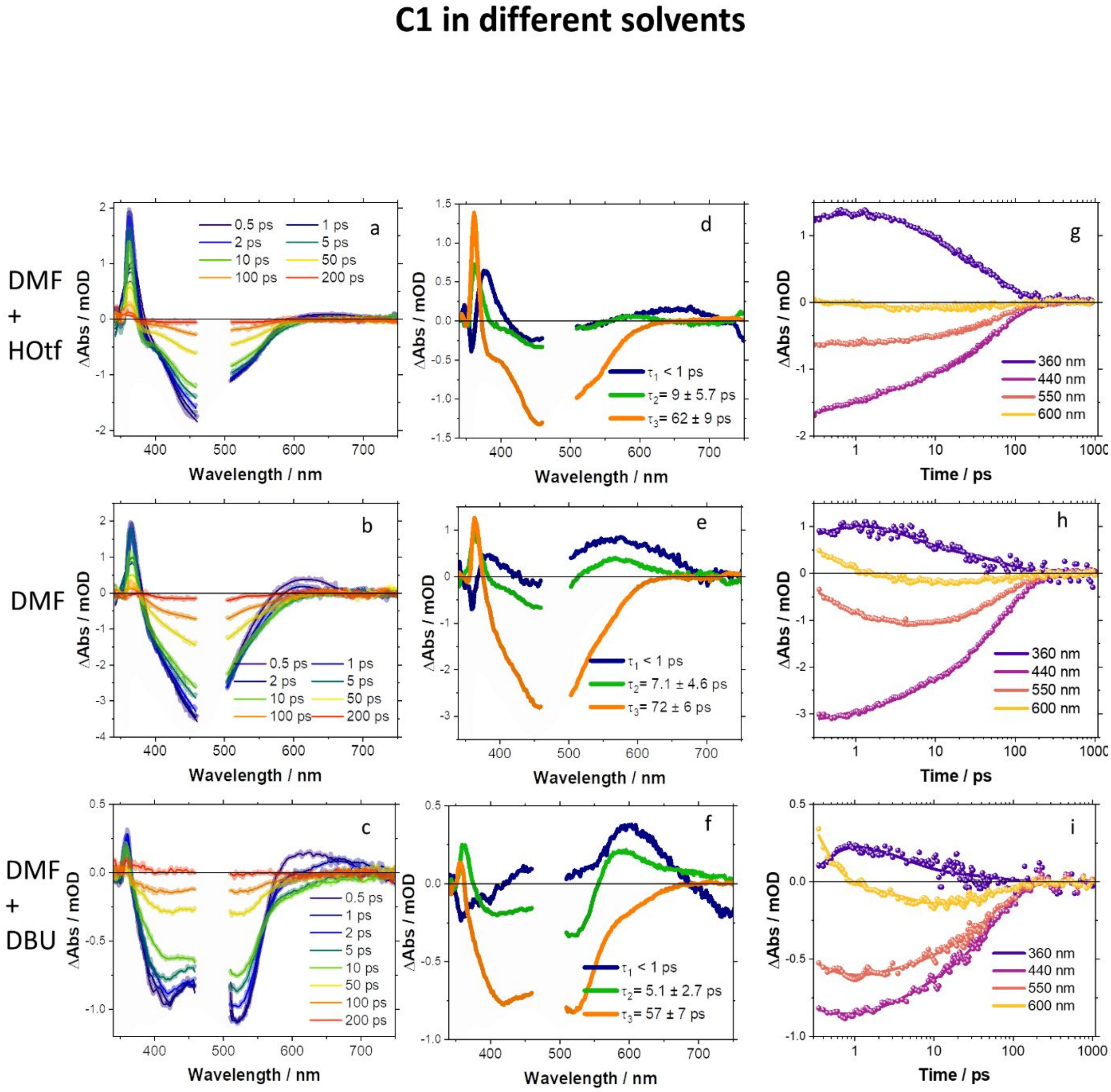

*Figure S 27 Transient absorption spectra at selected delay times (a-c), decay associated spectra (d-f) and kinetics at selected wavelengths (g-i) of complex **C1** in different solvents. Row 1: DMF+ HOtf i.e., fully protonated, row 2: DMF i.e partially protonated and row 3: DMF+DBU i.e fully deprotonated.*

C2 in different solvents

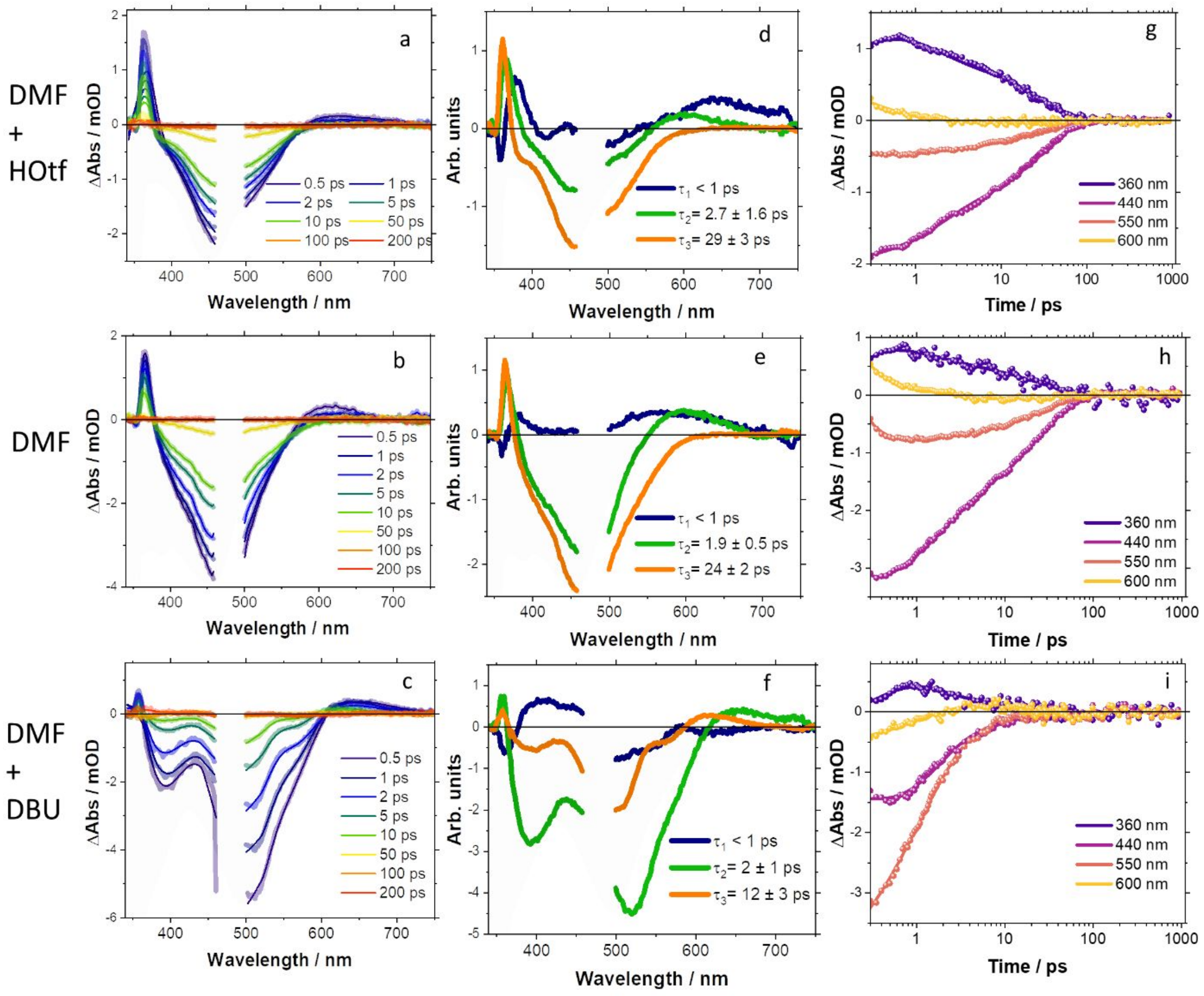

*Figure S 28: Transient absorption spectra at selected delay times (a-c), decay associated spectra (d-f) and kinetics at selected wavelengths (g-i) of complex **C2** in different solvent mixtures. Row 1: DMF+ HOtf i.e., fully protonated, row 2: DMF i.e partially protonated and row 3: DMF+DBU i.e fully deprotonated. The grey region shows the inverted steady state absorption.*

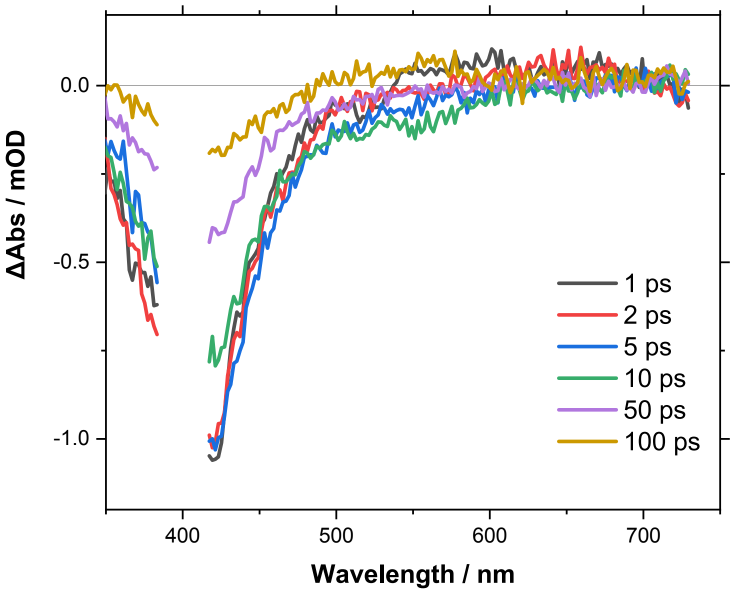

*Figure S 29: TAS of parent complex **P1** in ACN.*

Table S 3: Lifetimes from global analysis

Complex	Solvent	τ_1 [ps]	τ_2 [ps]	τ_3 [ps]
**C2**	DMF	0.2 ± 0.2	1.9 ± 0.5	24.5 ± 2.3
	DMF/HOTf[a]	0.3 ± 0.3	2.7 ± 1.6	29.0 ± 2.7
	DMF/DBU[a]	0.5 ± 0.4	2.0 ± 1.0	12.2 ± 3.4
**C1**	DMF	0.6 ± 0.4	7.1 ± 4.6	72.2 ± 6.4
	DMF/HOTf[a]	0.4 ± 0.2	9.0 ± 5.7	62.5 ± 8.7
	DMF/DBU[a]	0.3 ± 0.1	5.1 ± 2.7	56.7 ± 7.0

Target Analysis

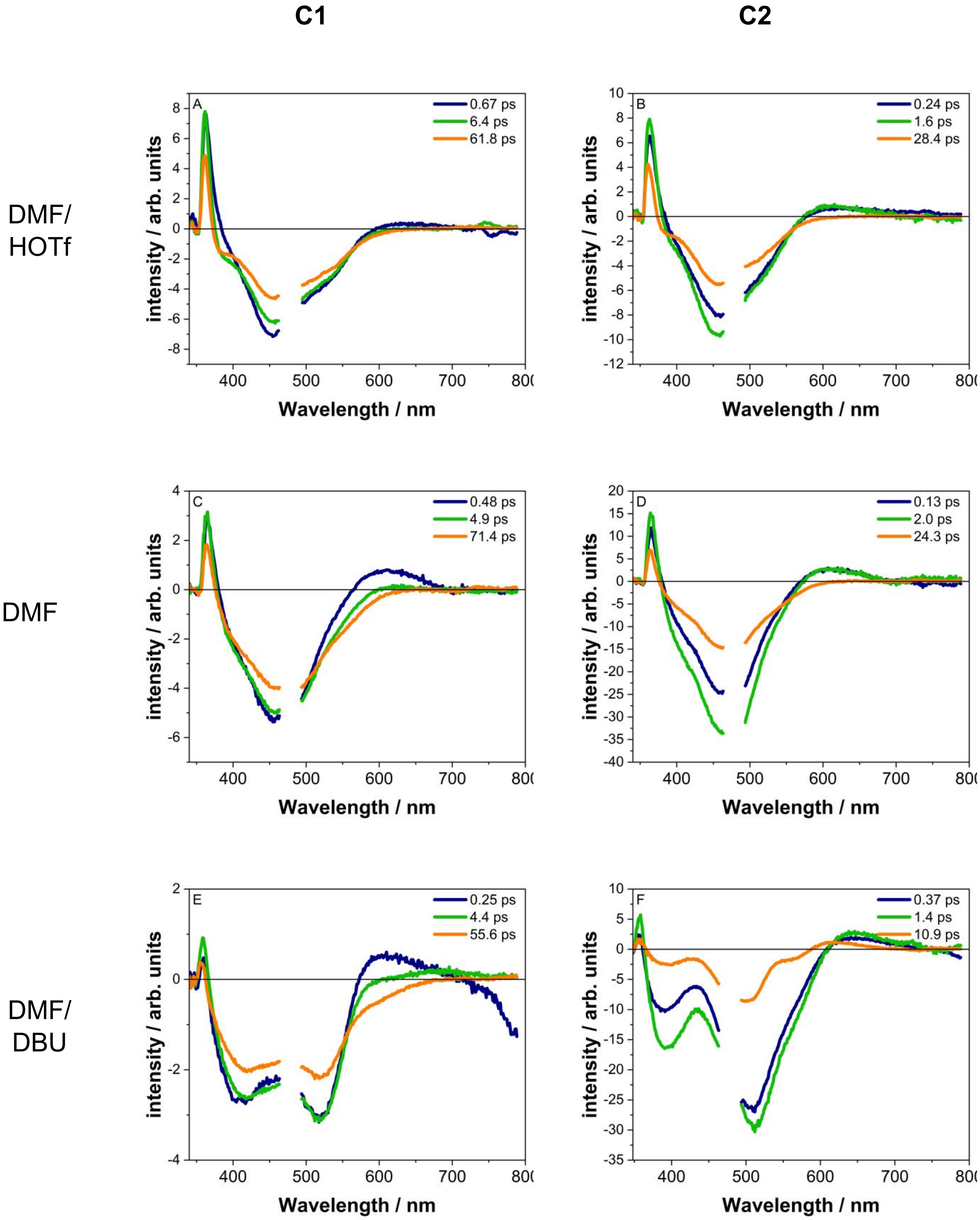

*Figure S 30: Species associated spectra of **C1** (left: A, C, D) and **C2** (right: B, D, E) in various solvent mixtures (DMF/HOTf: A, B; DMF: C, D; DMF/DBU: E, F) with Φ value of 1 **C1**. Φ did converge for **C2** close to the initialization value, therefore we have used Φ = 0.5 for SAS as examples. Φ values were obtained from target analysis using the model described in Figure 5 with fixed values for lifetimes τ_1 to τ_3.*

8. Electrochemical Investigations

*Table S 4: Electrochemical properties of the complex **P1** in ACN; values relative to Fc/Fc⁺ couple.*

Process	Potential / V
Fe^{2+}/Fe^{3+} (rev.)	0.494
Red.1 (irrev.)	-2.037
Red.2 (irrev.)	-2.606
Red.3 (irrev.)	-2.901

*Table S 5: Electrochemical properties of the complex **C1** in ACN; values relative to Fc/Fc⁺ couple.*

Process	Potential / V
Fe^{2+}/Fe^{3+} (rev.)	0.272
Red.1 (irrev.)	-1.975
Red.2 (irrev.)	-2.305
Red.3 (irrev.)	-2.699

*Table S 6: Electrochemical properties of the complex **C2** in ACN; values relative to Fc/Fc⁺ couple.*

Process	Potential / V
Fe^{2+}/Fe^{3+} (rev.)	0.280
Red.1 (irrev.)	-1.975
Red.2 (irrev.)	-2.354
Red.3 (irrev.)	-2.704

*Table S 7: Electrochemical properties of the complex **C1^{prot}** in DMF; values relative to Fc/Fc^{+} couple*

Process	Potential / V
Fe^{2+}/Fe^{3+} (rev.)	0.098
Red.1 (irrev.)	-2.372
Red.2 (irrev.)	-2.617
Red.3 (irrev.)	-2.707

*Table S 8: Electrochemical properties of the complex **C1^{deprot}** in DMF; values relative to Fc/Fc^{+} couple*

Process	Potential / V
Fe^{2+}/Fe^{3+} (rev.)	-0.375
Red.1 (irrev.)	---
Red.2 (irrev.)	---
Red.3 (irrev.)	-2.707

*Table S 9: Electrochemical properties of the complex **C2^{prot}** in DMF; values relative to Fc/Fc^{+} couple.*

Process	Potential / V
Fe^{2+}/Fe^{3+} (rev.)	0.087
Red.1 (irrev.)	-2.452
Red.2 (irrev.)	-2.732

*Table S 10: Electrochemical properties of the complex **C2^{deprot}** in DMF; values relative to Fc/Fc^{+} couple.*

Process	Potential / V
Fe^{2+}/Fe^{3+} (rev.)	-0.447
Red.1 (irrev.)	---
Red.2 (irrev.)	-2.682

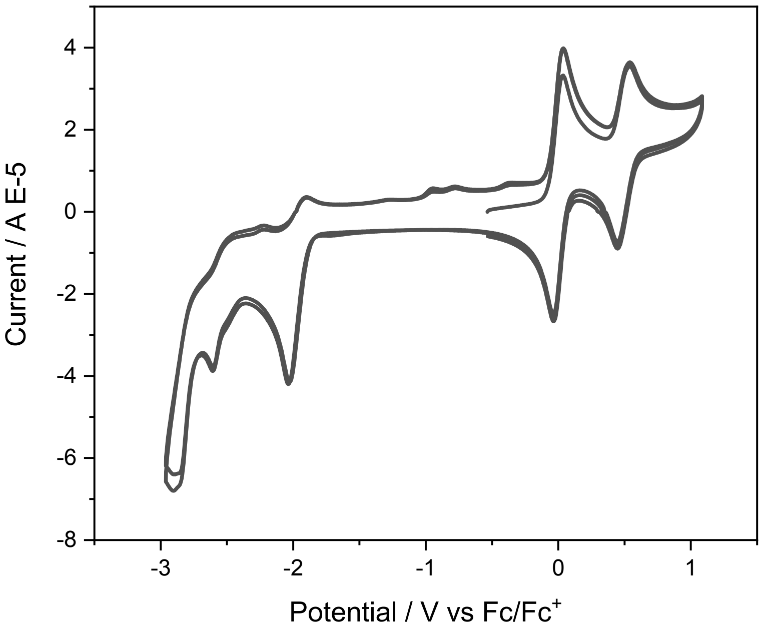

*Figure S 31: Cyclic voltammogram of **P1**, 1 mM solution in ACN, 0.1 M TBAPF$_6$, referenced against Fc/Fc$^+$ as internal reference. .*

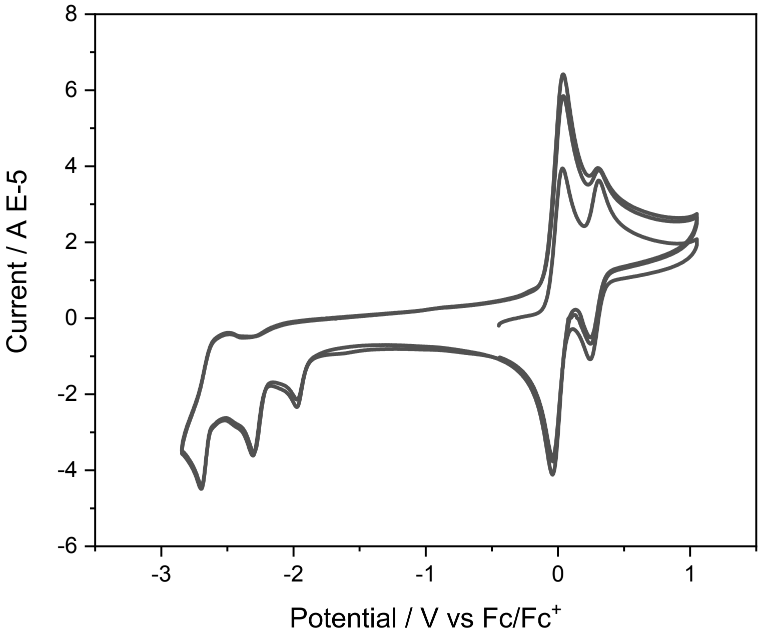

*Figure S 32: Cyclic voltammogram of **C1^{prot}**, 1 mM solution in ACN, 0.1 M TBAPF$_6$, referenced against Fc/Fc$^+$ as internal reference.*

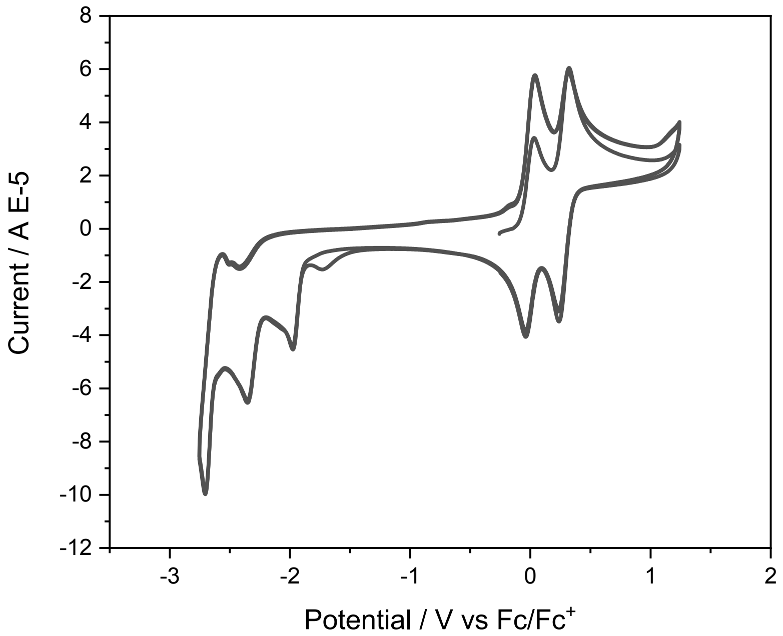

*Figure S 33: Cyclic voltammogram of **C2^{prot}**, 1 mM solution in ACN, 0.1 M TBAPF$_6$, referenced against Fc/Fc$^+$ as internal reference.*

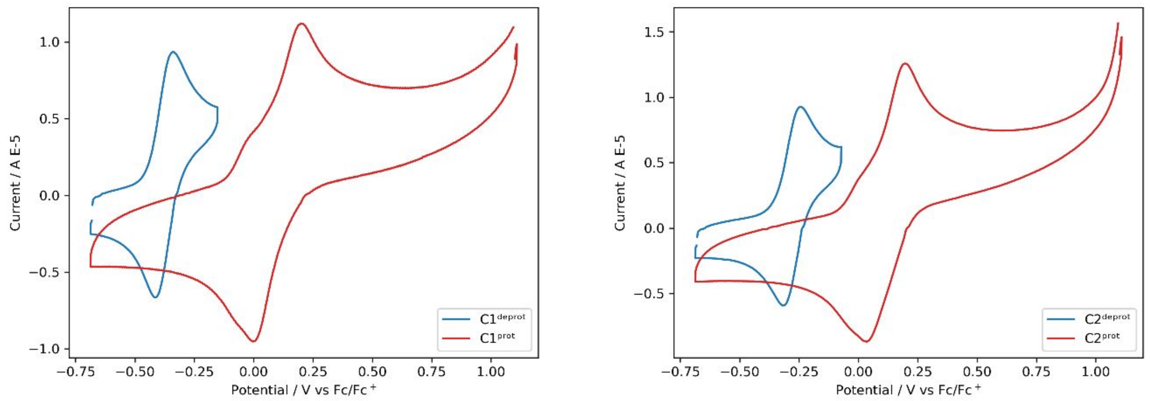

*Figure S 34: Oxidation process of complex protonated (red) and deprotonated (blue) **C1** (left) and **C2** (right), 1 mM solution in DMF, 0.1 M TBAPF$_6$, referenced against Fc/Fc$^+$ as external reference .*

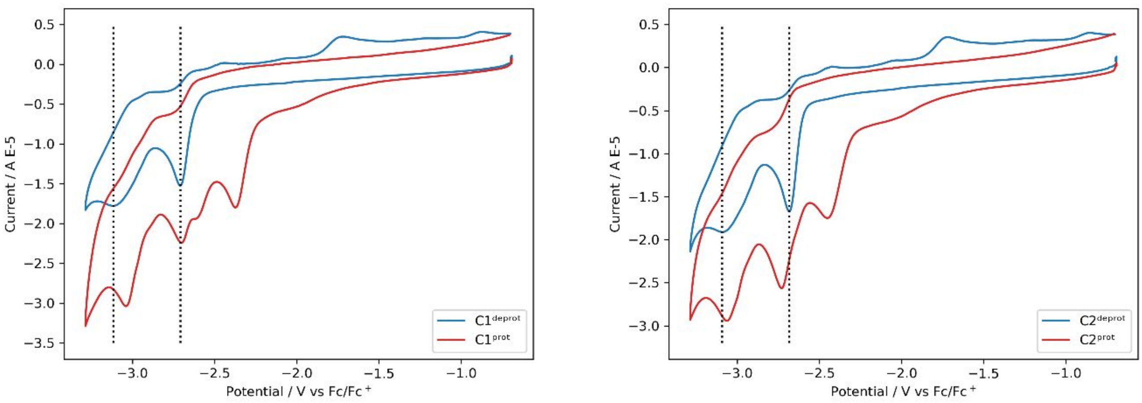

Figure S 35: Reduction process of complex protonated (red) and deprotonated (blue) ***C1*** *(left) and* ***C2*** *(right), 1 mM solution in DMF, 0.1 M $TBAPF_6$, referenced against Fc/Fc^+ as external reference.*

9. Raman Spectroscopy

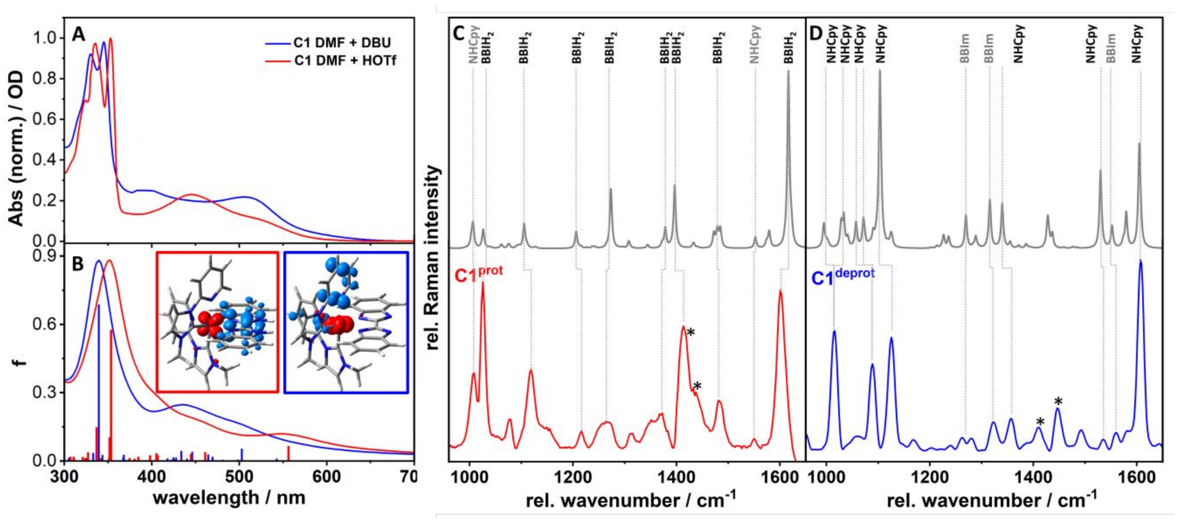

Figure S 36: *A: UV–vis absorption spectra of protonated (red) and deprotonated (blue)* ***C1*** *in DMF/HOTf and DMF/DBU, respectively (A) and TDDFT-calculated UV-vis absorption spectra including calculated charge density difference images illustrating the excited-state re-localization upon protonation for* ***C1*** *(B). Comparison of the experimental (red for* ***C1^{prot}*** *and blue for* ***C1^{deprot}****) and calculated (grey) rR spectra recorded at an excitation wavelength of 473 (****C1^{prot}****) and 532 nm (****C1^{deprot}****). Ligand-affiliated Raman modes are labeled with* ***BBI*** *and* ***^{Me}NHCpy****. Bands marked with an asterisk are residues of solvent features due to the subtraction of the solvent spectrum.*

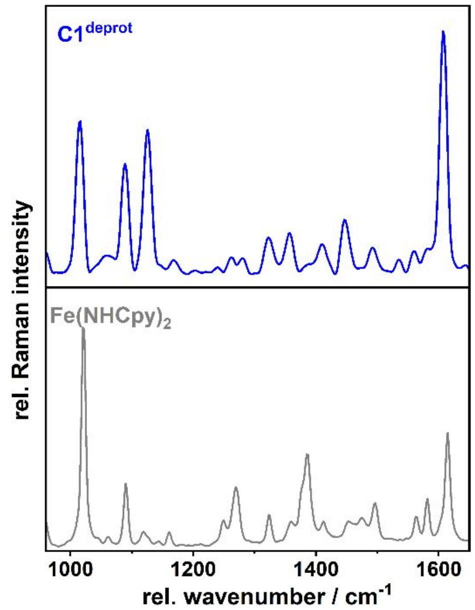

Figure S 37: Experimental rR spectrum of deprotonated ***C1*** *(in DMF/DBU) excited at 532 nm (blue, top) in comparison to the non-resonant Raman spectrum of the reference complex* ***P1*** *(grey, below), excited at 1064 nm.*

10. Theoretical Calculations

*Table S 11: Simulated Fe-N_{py}, Fe-C_{py}, and Fe-N_{BBI} bond lengths and dihedral angles (**δ (°)**) of bi-benzimidazole for deprotonated and protonated **C1** and **C2** in solvents (DMF and ACN). The values are given withing optimized singlet ground state as well as fully relaxed 3MLCT, 3MC and 5MC geometries.*

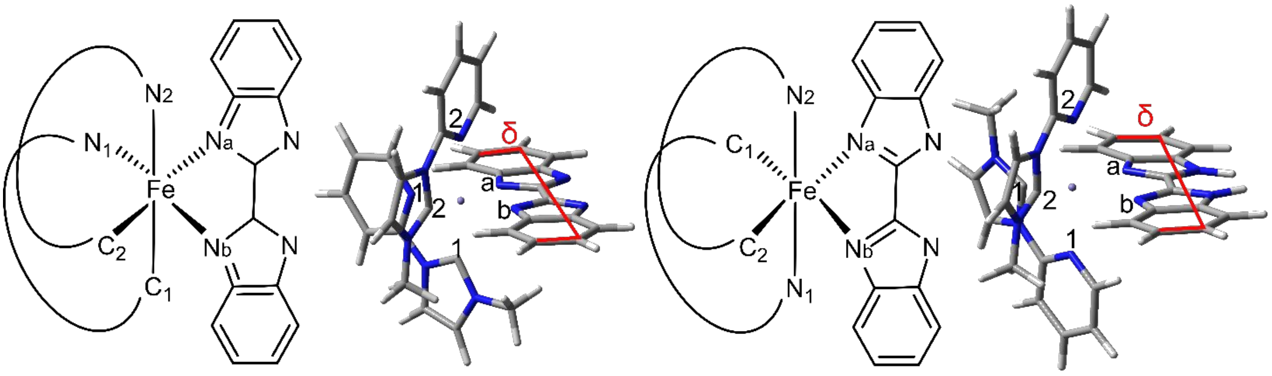

			Distance (Å)						δ (°)
			Fe-N_1	Fe-N_2	Fe-**C1**	Fe-**C2**	Fe-N_a	Fe-N_a	BBI
**DMF**	**C1**^{deprot}	GS	1.980	2.040	1.881	1.890	2.034	1.987	0.5
		^{3}MC	2.735	2.100	1.964	1.886	2.000	2.073	2.6
		^{5}MC	2.284	2.361	2.109	2.131	2.182	2.113	4.1
	**C2**^{deprot}	GS	1.981	1.981	1.893	1.893	2.028	2.028	1.9
		^{3}MLCT	1.978	1.978	1.931	1.931	2.019	2.019	5.4
		^{3}MC	2.326	2.316	1.932	1.929	2.008	2.010	0.5
		^{5}MC	2.251	2.251	2.136	2.136	2.185	2.185	6.1
	**C1**^{prot}	GS	1.983	2.040	1.894	1.897	2.046	1.991	1.0
	**C2**^{prot}	GS	1.985	1.985	1.895	1.895	2.042	2.042	2.6
**ACN**	**C1**^{deprot}	GS	1.981	2.040	1.881	1.890	2.034	1.986	0.6
	**C2**^{deprot}	GS	1.981	1.981	1.893	1.893	2.028	2.028	1.9
	**C1**^{prot}	GS	1.983	2.040	1.895	1.897	2.046	1.990	1.0
		^{3}MLCT	1.999	2.070	1.920	1.937	1.947	1.999	2.0
		^{3}MC	2.340	2.054	1.957	1.917	2.058	2.255	3.6
		^{5}MC	2.244	2.311	2.108	2.108	2.271	2.192	6.1
	**C2**^{prot}	GS	1.986	1.986	1.895	1.895	2.042	2.042	2.6
		^{3}MLCT	1.987	1.987	1.935	1.935	1.993	1.993	6.6
		^{3}MC	2.306	2.306	1.934	1.934	2.035	2.035	-0.1
		^{5}MC	2.228	2.228	2.114	2.114	2.274	2.274	6.6

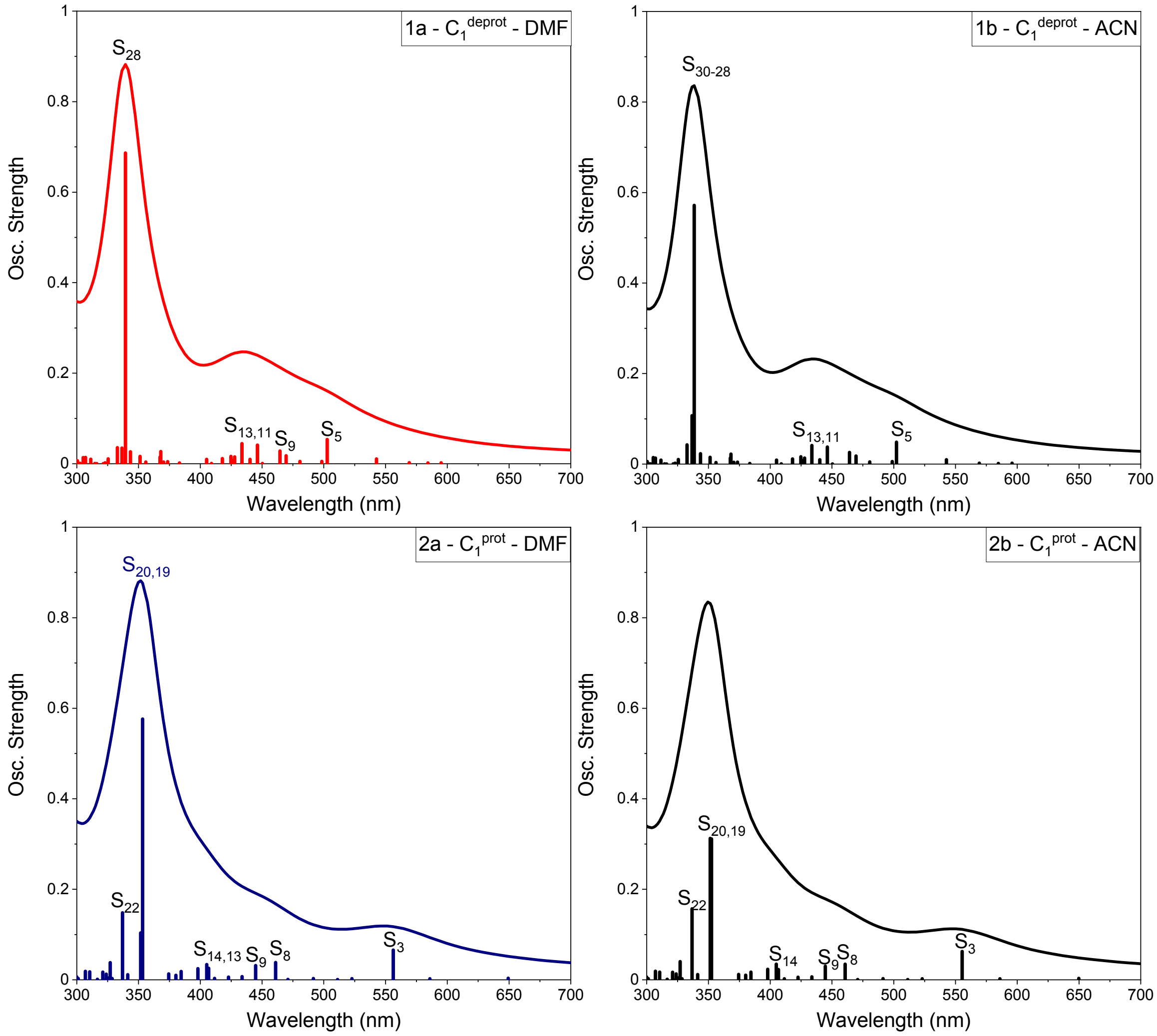

Figure S 38: Simulated absorption spectra of deprotonated (A1) and protonated (B1) ***C1*** *in DMF and ACN (A2 and B2); key electronic excitations contributing to the absorption in the visible region are indicated. The transitions were broadened by Lorentzian functions with a full width at half maximum of 0.2 eV.*

Table S 12: Calculated vertical excitation energies (E^e), wavelengths (λ), oscillator strengths (f), and singly-excited configurations of the main excited singlet-singlet transitions involved in the initial absorption of ***deprotonated C1 in DMF****.*

State	Transition Type	Weight / %	E^e / eV	λ / nm	f
S_5	$MLCT_{NHCp}$	72	2.47	503	0.053
S_9	$MLCT_{NHCp}$	50	2.67	464	0.028
	ILCT, $MLCT_{NHCp}$	19			
S_{11}	$MCLT_{NHCp,BBI}$	67	2.78	445	0.041
S_{13}	$MCLT_{NHCp}$	25	2.86	434	0.044
	ILCT, $MLCT_{NHCp}$	17			
S_{28}	$ILCT_{BBI}$	71	3.65	339	0.687

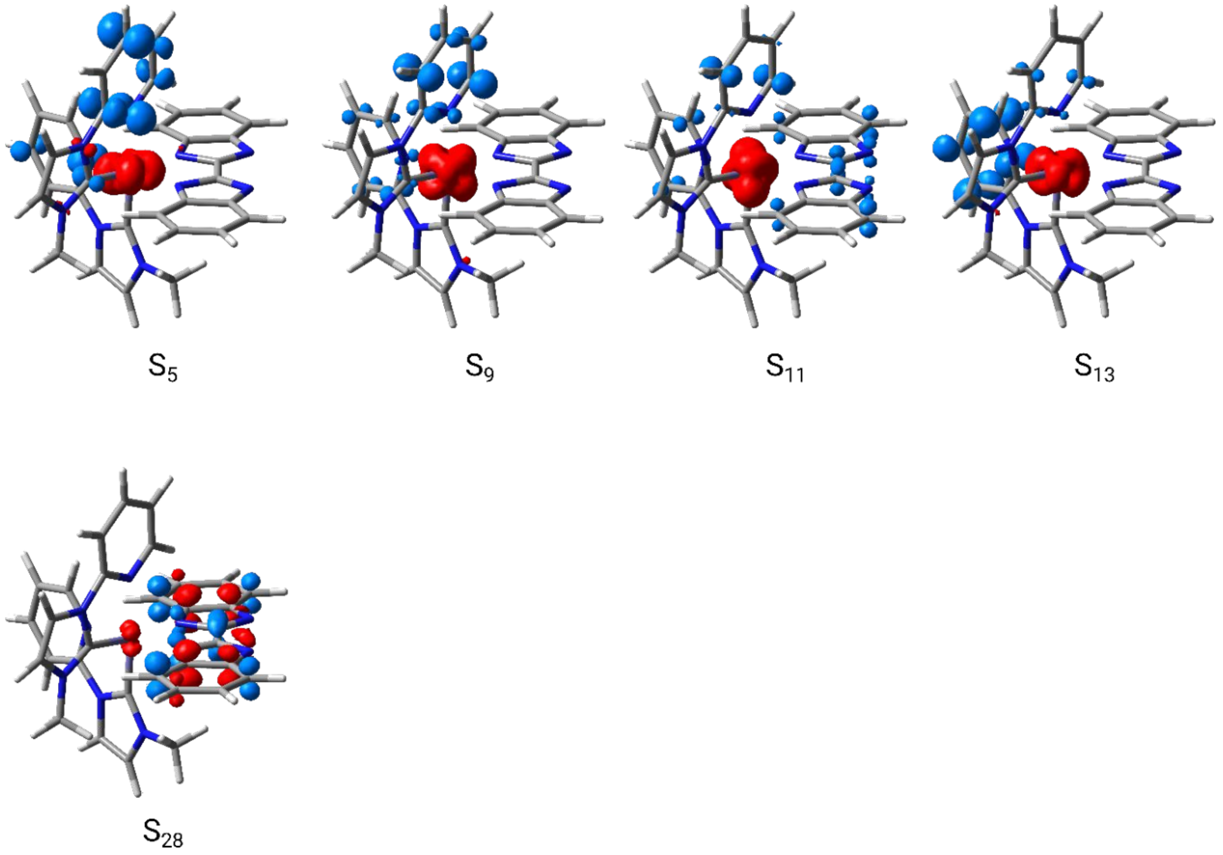

Figure S 39: Charge density differences (CDDs) of prominent singlet-singlet excitations involved in the UV/vis absorption of ***deprotonated C1 in DMF*** *within the Franck-Condon region. Charge transfer takes place from red to blue.*

*Table S 13: Calculated vertical excitation energies (E^e), wavelengths (λ), oscillator strengths (f), and singly-excited configurations of the main excited singlet-singlet transitions involved in the initial absorption of **deprotonated C1 in ACN**.*

State	Transition Type	Weight / %	E^e / eV	λ / nm	f
S_5	$MLCT_{NHCp}$	71	2.47	502	0.048
S_{11}	$MLCT_{BBI}$	65	2.78	446	0.038
	$MLCT_{NHCp}$	15			
S_{13}	$MLCT_{BBI}$	22	2.86	434	0.041
	$MLCT_{NHCp}$	18			
	$MLCT_{NHCp}$	15			
S_{28}	$ILCT_{BBI}$	61	3.66	338	0.572
	LLCT	17			
S_{29}	LLCT	79	3.68	337	0.107
	$ILCT_{BBI}$	14			
S_{30}	$MLCT_{NHCp}$	23	3.73	333	0.043
	$MLCT_{NHCp}$	14			

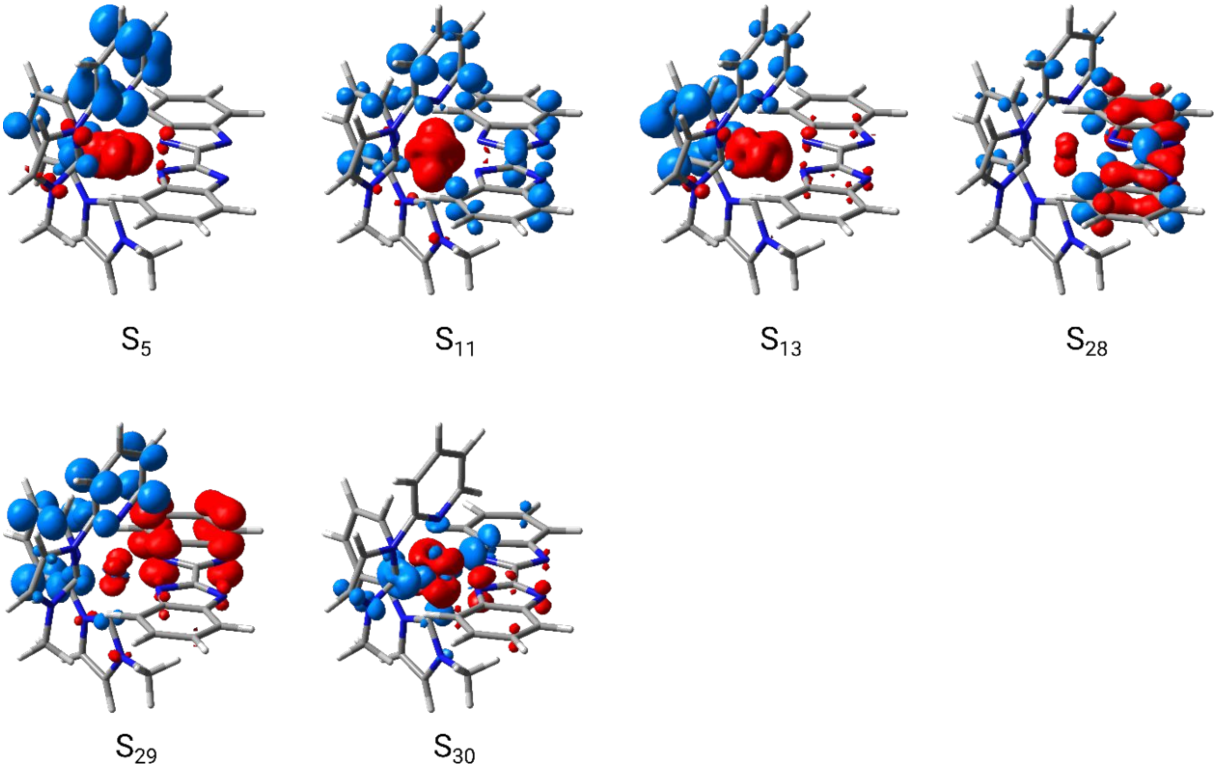

*Figure S 40: Charge density differences (CDDs) of prominent singlet-singlet excitations involved in the UV/vis absorption of **deprotonated C1 in ACN** within the Franck-Condon region. Charge transfer takes place from red to blue.*

*Table S 14: Calculated vertical excitation energies (E^e), wavelengths (λ), oscillator strengths (f), and singly-excited configurations of the main excited singlet-singlet transitions involved in the initial absorption of **protonated C1 in DMF**.*

State	Transition Type	Weight / %	E^e / eV	λ / nm	f
S_3	$MLCT_{BBI}$	94	2.23	556	0.066
S_8	ILCT, $MLCT_{NHCp}$	40	2.69	461	0.038
	MC, $LMCT_{NHCp}$	21			
S_9	$MLCT_{NHCp}$	33	2.79	445	0.031
	$MLCT_{NHCp}$	23			
	$MLCT_{NHCp}$, ILCT, MC	13			
S_{13}	$MLCT_{NHCp}$	70	3.05	407	0.026
S_{14}	$MLCT_{NHCp}$	34	3.06	405	0.034
S_{19}	$ILCT_{BBI}$	82	3.51	353	0.586
S_{20}	MC, $LMCT_{NHCp}$	27	3.52	352	0.104
	MC, $LMCT_{NHCp}$	25			
	MC, $LMCT_{NHCp}$	19			
S_{22}	LLCT, $ILCT_{BBI}$	82	3.68	337	0.148

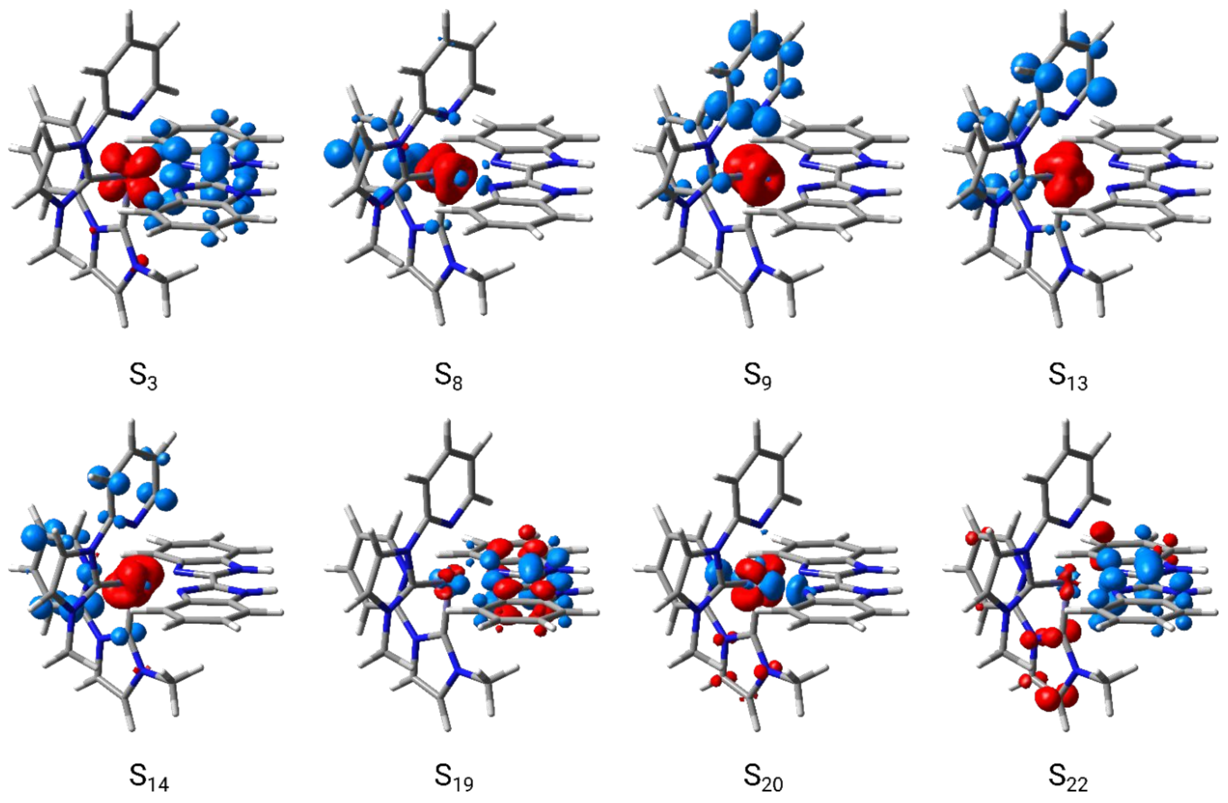

*Figure S 41: Charge density differences (CDDs) of prominent singlet-singlet excitations involved in the UV/vis absorption of **protonated C1 in DMF** within the Franck-Condon region. Charge transfer takes place from red to blue.*

Table S 15: Calculated vertical excitation energies (E^e), wavelengths (λ), oscillator strengths (f), and singly-excited configurations of the main excited singlet-singlet transitions involved in the initial absorption of ***protonated C1 in ACN****.*

State	Transition Type	Weight / %	E^e / eV	λ / nm	f
S_3	$MLCT_{BBI}$	94	2.23	555	0.063
S_8	$MLCT_{NHCp}$	38	2.69	460	0.034
	MC	21			
S_9	$MLCT_{NHCp}$	33	2.79	444	0.030
	$MLCT_{NHCp}$	24			
	$MLCT_{NHCp}$	13			
S_{14}	$MLCT_{NHCp}$	32	3.06	405	0.035
S_{19}	$ILCT_{BBI}$	49	3.52	352	0.311
	MC	15			
	MC	14			
S_{20}	$ILCT_{BBI}$	42	3.53	351	0.313
	MC	16			
	MC	16			
S_{22}	LLCT	95	3.68	337	0.157

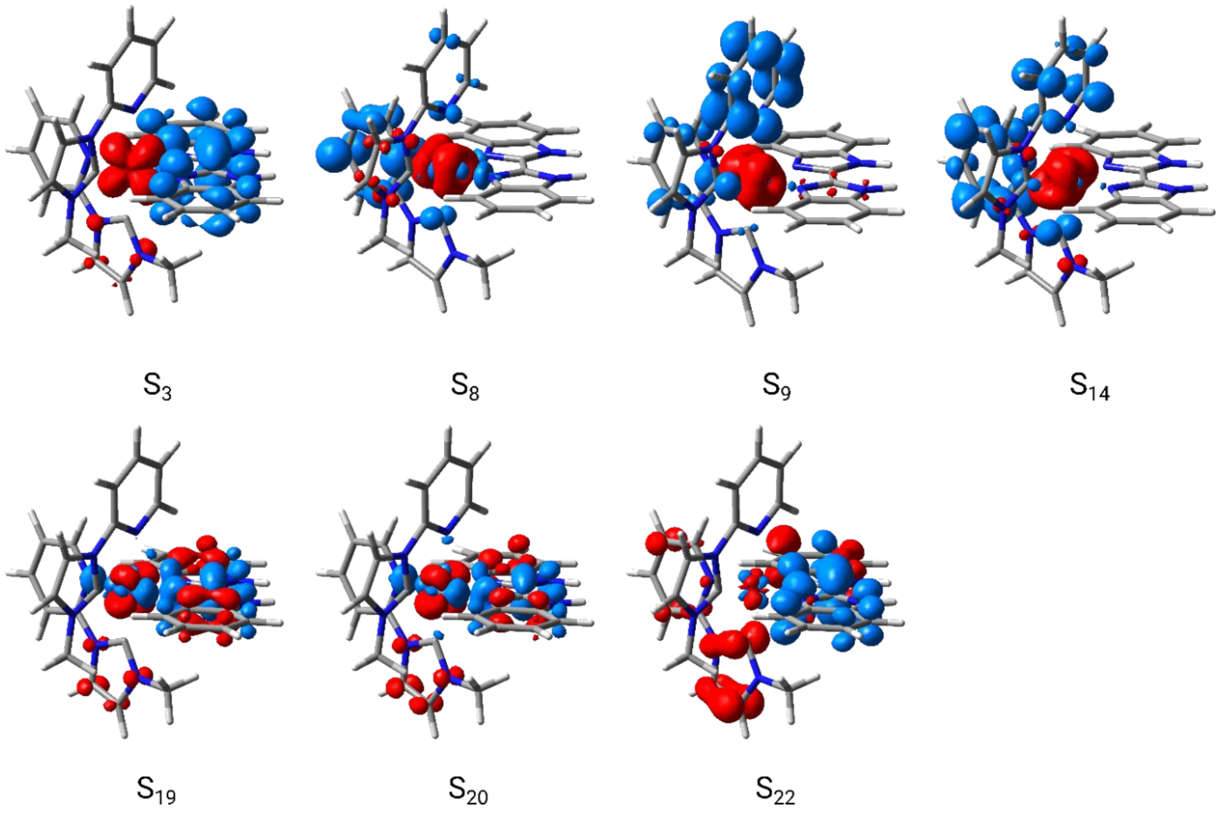

Figure S 42: Charge density differences (CDDs) of prominent singlet-singlet excitations involved in the UV/vis absorption of ***protonated C1 in ACN*** *within the Franck-Condon region. Charge transfer takes place from red to blue.*

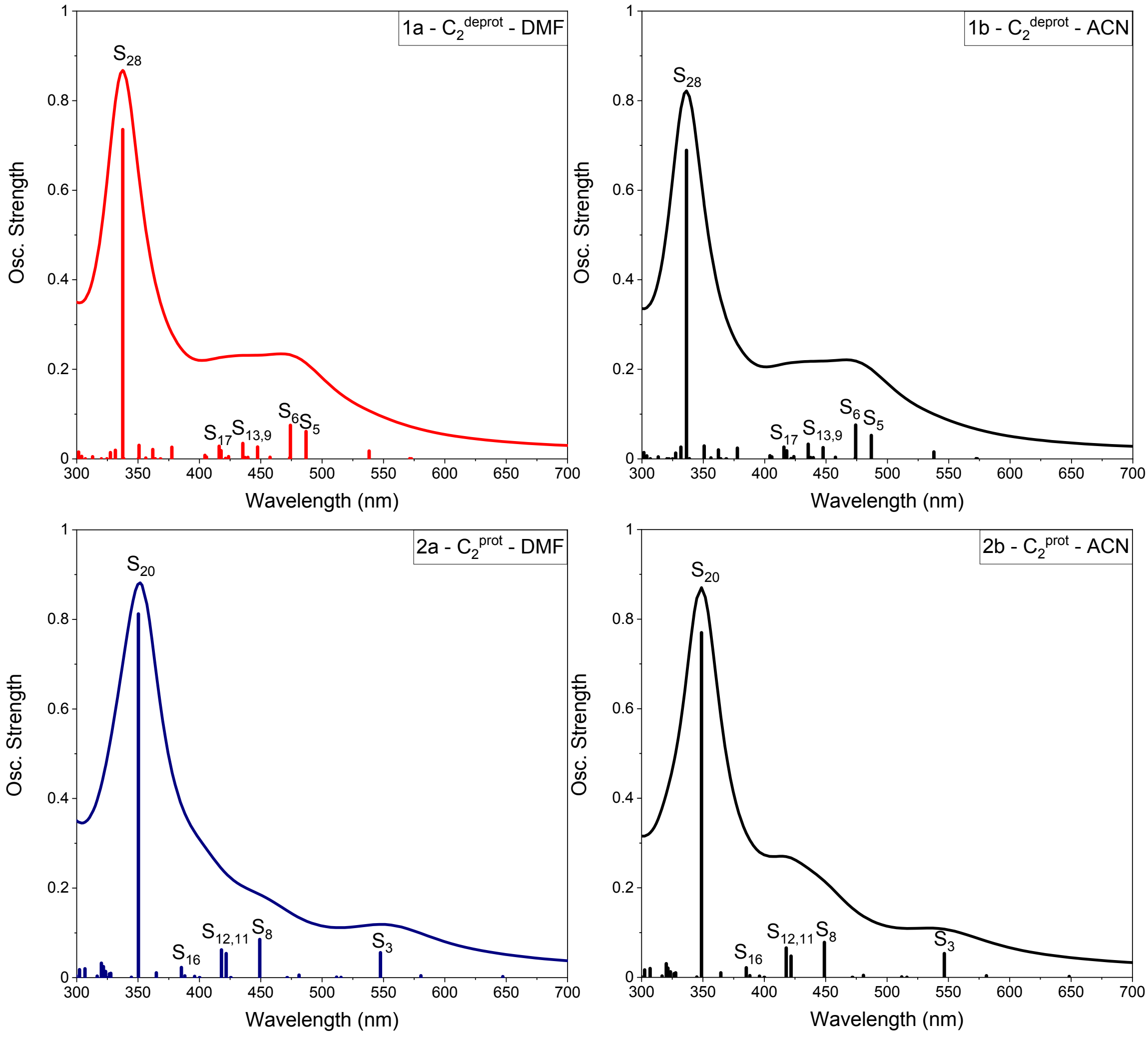

Figure S 43: Simulated absorption spectra of deprotonated (A1) and protonated (B1) ***C2*** *in DMF (a) and ACN (A2 and B2); key electronic excitations contributing to the absorption in the visible region are indicated. The transitions were broadened by Lorentzian functions with a full width at half maximum of 0.2 eV.*

*Table S 16: Calculated vertical excitation energies (E^e), wavelengths (λ), oscillator strengths (f), and singly-excited configurations of the main excited singlet-singlet transitions involved in the initial absorption of **deprotonated C2 in DMF**.*

State	Transition Type	Weight %	E^e/eV	λ/nm	f
S_5	$MLCT_{NHCp}$	57	2.55	487	0.061
	$MLCT_{NHCp}$	26			
S_6	$MLCT_{NHCp}$	72	2.62	474	0.075
	$MLCT_{NHCp}$	17			
S_9	MC, $MLCT_{NHCp}$	59	2.77	447	0.027
	MC, $MLCT_{NHCp}$	26			
S_{13}	$MLCT_{NHCp}$, $ILCT_{NHCp}$	74	2.85	435	0.035
	$MLCT_{NHCp}$, $ILCT_{NHCp}$	22			
S_{17}	$MLCT_{BBI}$	73	2.98	416	0.028
S_{28}	$ILCT_{BBI}$	87	3.67	337	0.735

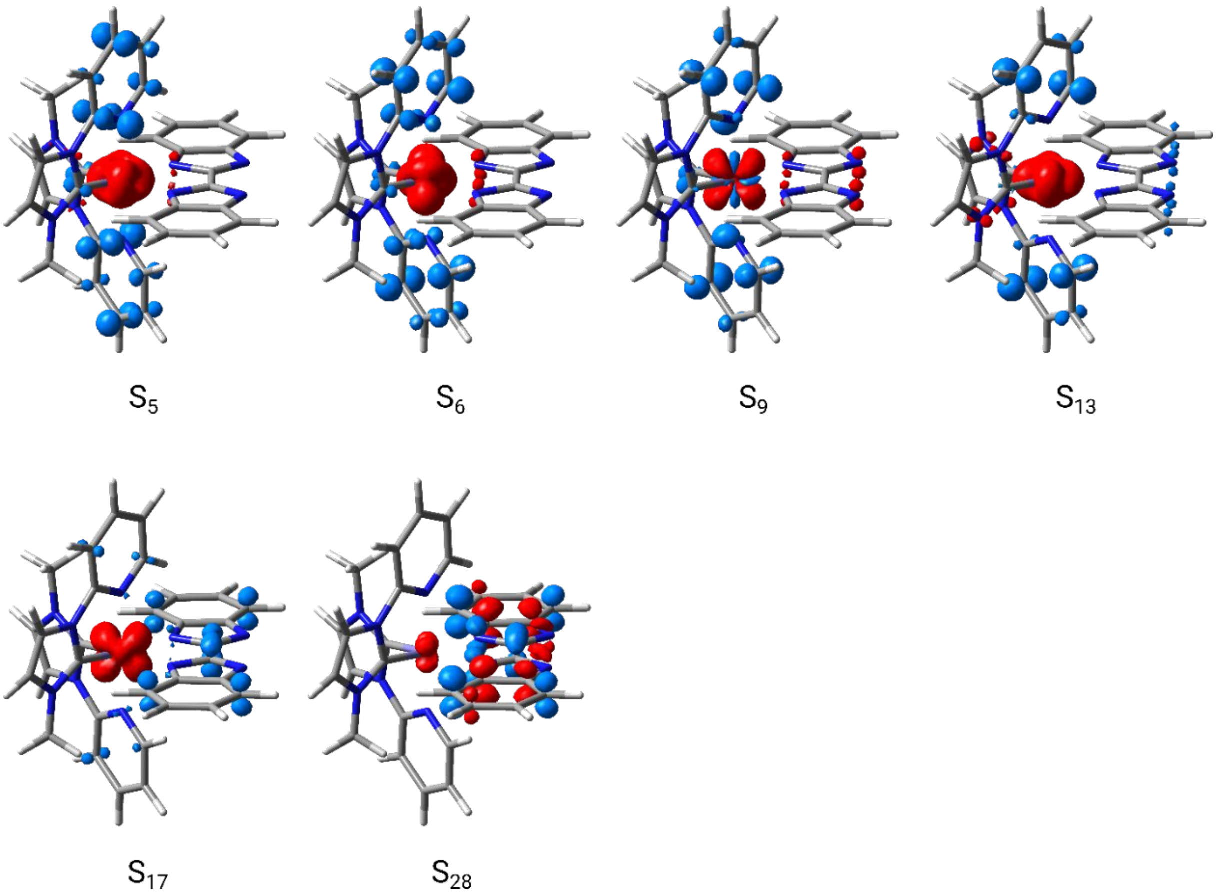

*Figure S 44: Charge density differences (CDDs) of prominent singlet-singlet excitations involved in the UV/vis absorption of **deprotonated C2 in DMF** within the Franck-Condon region. Charge transfer takes place from red to blue.*

*Table S 17: Calculated vertical excitation energies (E^e), wavelengths (λ), oscillator strengths (f), and singly-excited configurations of the main excited singlet-singlet transitions involved in the initial absorption of **deprotonated C2 in ACN**.*

State	Transition Type	Weight / %	E^e / eV	λ / nm	f
S_5	$MLCT_{NHCp}$	53	2.54	486	0.052
	$MLCT_{NHCp}$	29			
S_6	$MLCT_{NHCp}$	69	2.62	474	0.076
	$MLCT_{NHCp}$	19			
S_9	$MLCT_{NHCp}$	58	2.77	448	0.025
	MC	26			
S_{13}	$MLCT_{NHCp}$, $ILCT_{NHCp}$	72	2.85	435	0.033
	$MLCT_{BBI}$	24			
S_{17}	$MLCT_{BBI}$	71	2.98	415	0.026
	$MLCT_{NHCp}$	14			
S_{28}	$ILCT_{BBI}$	85	3.69	336	0.689

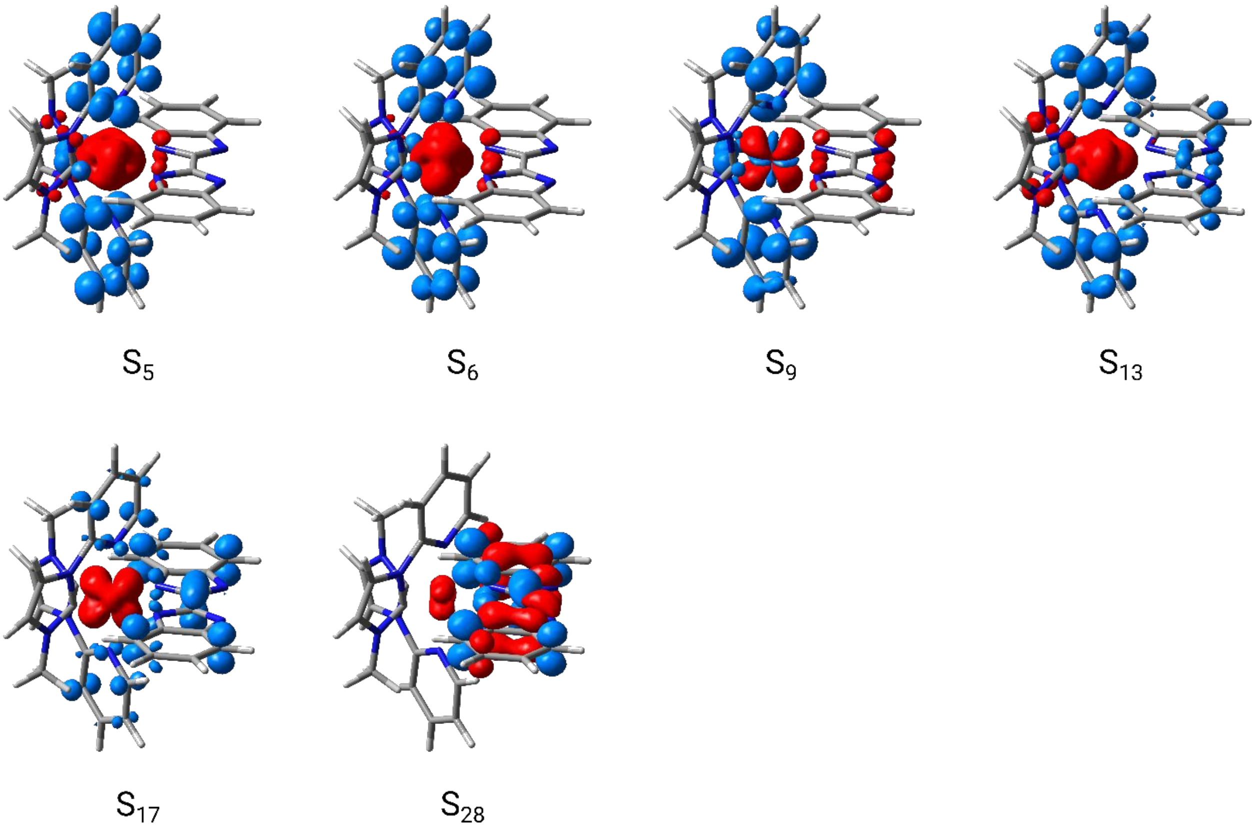

*Figure S 45: Charge density differences (CDDs) of prominent singlet-singlet excitations involved in the UV/vis absorption of **deprotonated C2 in ACN** within the Franck-Condon region. Charge transfer takes place from red to blue.*

*Table S 18: Calculated vertical excitation energies (E^e), wavelengths (λ), oscillator strengths (f), and singly-excited configurations of the main excited singlet-singlet transitions involved in the initial absorption of **protonated C2 in DMF**.*

State	Transition Type	Weight / %	E^e / eV	λ / nm	f
S_3	$MLCT_{BBI}$	94	2.26	547	0.056
S_8	$MLCT_{NHCp}$	56	2.76	449	0.085
	MC	24			
S_{11}	$MLCT_{NHCp}$	93	2.94	422	0.054
S_{12}	MC	47	2.97	417	0.062
	$MLCT_{NHCp}$, MC	17			
S_{16}	$MLCT_{NHCp}$	83	3.22	385	0.023
S_{20}	$ILCT_{BBI}$	97	3.54	350	0.812

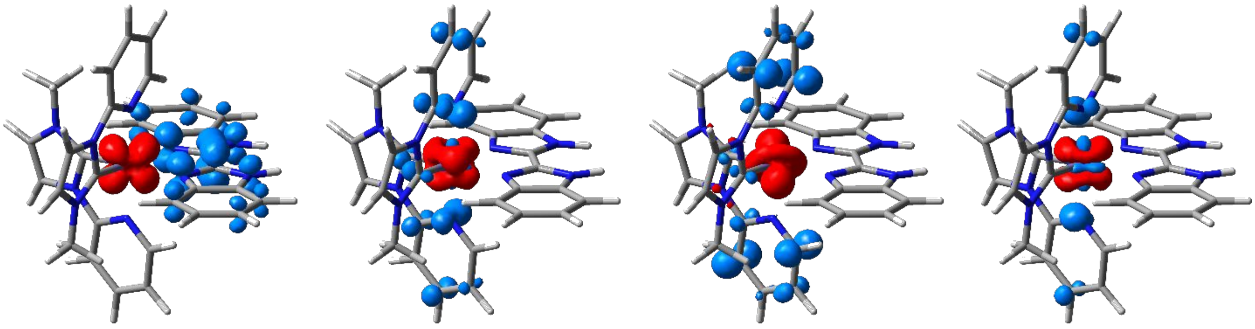

S_3 S_8 S_{11} S_{12}

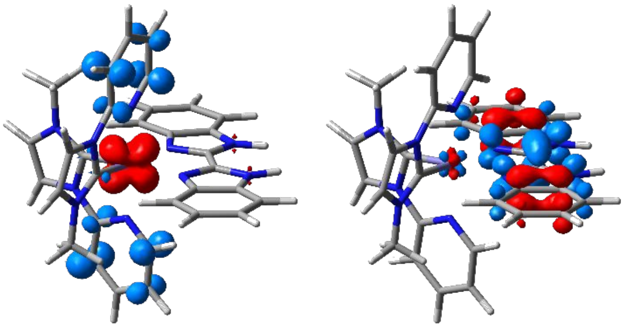

S_{16} S_{20}

*Figure S 46: Charge density differences (CDDs) of prominent singlet-singlet excitations involved in the UV/vis absorption of **protonated C2 in DMF** within the Franck-Condon region. Charge transfer takes place from red to blue.*

*Table S 19: Calculated vertical excitation energies (E^e), wavelengths (λ), oscillator strengths (f), and singly-excited configurations of the main excited singlet-singlet transitions involved in the initial absorption of **protonated C2 in ACN**.*

State	Transition Type	Weight / %	E^e / eV	λ / nm	f
S_3	$MLCT_{BBI}$	94	2.27	547	0.053
S_8	$MLCT_{NHCp}$	54	2.76	449	0.078
	MC	25			
S_{11}	$MLCT_{NHCp}$	93	2.93	422	0.048
S_{12}	MC	45	2.97	418	0.066
	$MLCT_{NHCp}$	18			
	$MLCT_{NHCp}$	14			
S_{16}	$MLCT_{NHCp}$	82	3.22	385	0.021
S_{20}	$ILCT_{BBI}$	96	3.52	352	0.770

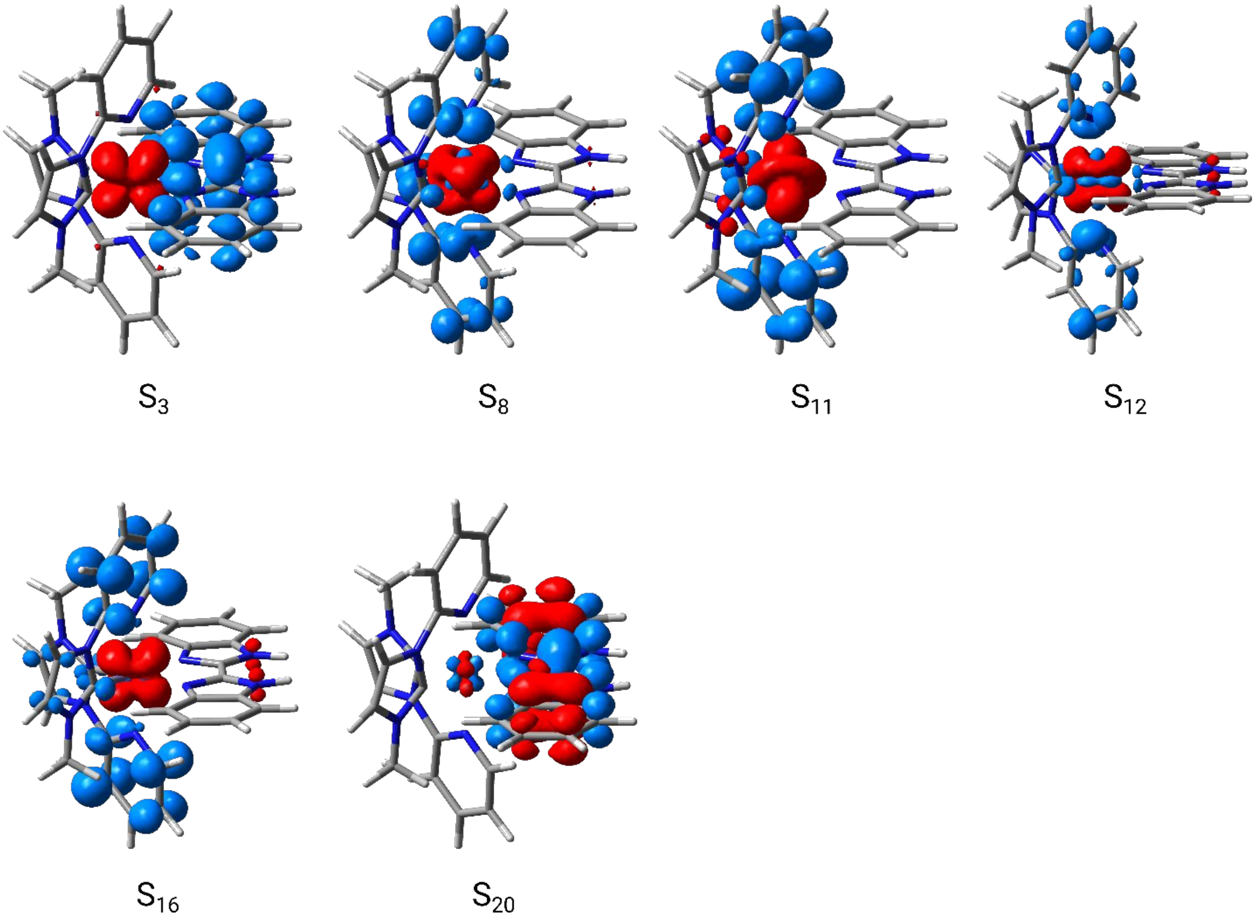

*Figure S 47: Charge density differences (CDDs) of prominent singlet-singlet excitations involved in the UV/vis absorption of **protonated C2 in ACN** within the Franck-Condon region. Charge transfer takes place from red to blue.*

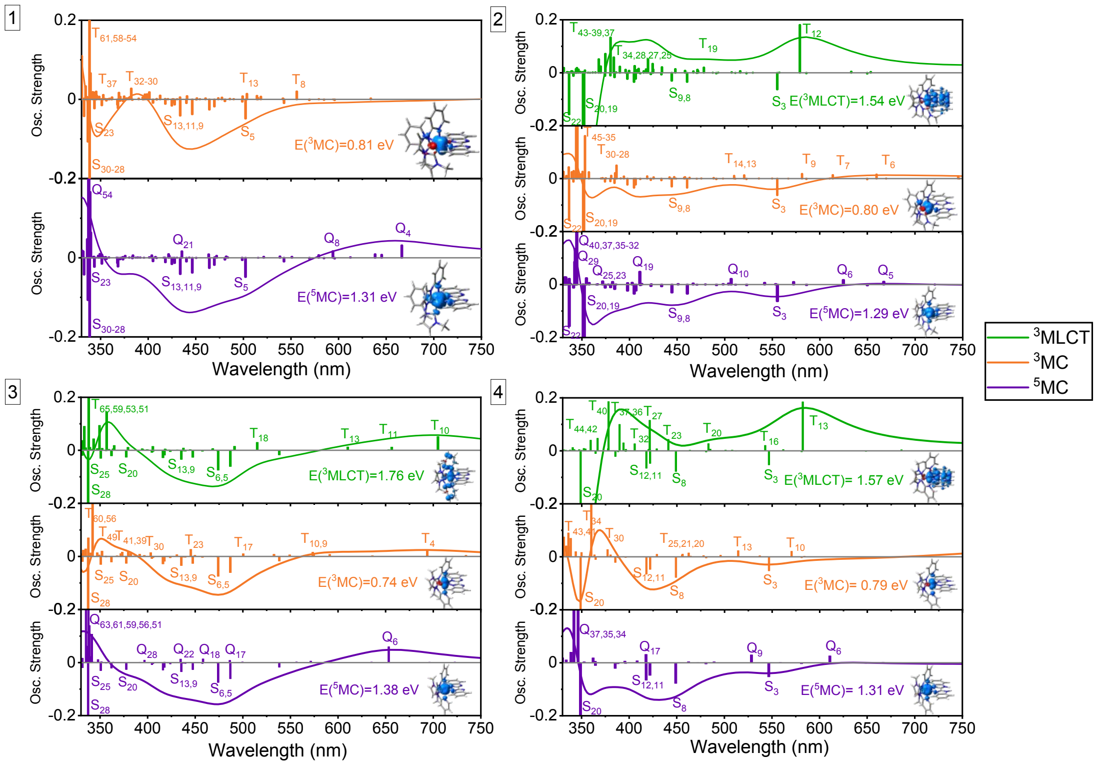

Figure S 48: Simulated transient absorption spectra of deprotonated (in DMF) (1, 3) and protonated (in ACN) (2, 4) ***C1*** *(1, 2) and* ***C2*** *(3, 4) within the fully relaxed ^{3}MLCT (green), ^{3}MC (orange), ^{5}MC (purple) equilibria; prominent singlet-singlet (ground-state bleach) as well as spin and dipole-allowed triplet-triplet excitations are indicated. The transitions were broadened by Lorentzian functions with a full width at half maximum of 0.2 eV. Spin densities of triplet species are given as inserts. The SCF energy of equilibrated triplet and quintet ground states are given relative to the respective ground state in FC region*

*Table S 20: Calculated vertical excitation energies (E^e), wavelengths (λ), oscillator strengths (f), spin contamination ($\langle s^2 \rangle$), and singly-excited configurations of the main excited triplet-triplet transitions involved in the initial absorption of **deprotonated C1 in DMF within ^{3}MC equilibrated geometry**.*

State	Transition Type	Weight / %	E^e / eV	λ / nm	f	s^2
T_8	$MLCT_{NHCp}$	89	2.23	556	0.020	3.16
T_{13}	$MLCT_{NHCp}$<br>$MLCT_{BBI}$, MC	36<br>15	2.46	504	0.014	2.89
T_{30}	LLCT	29	3.09	401	0.018	2.84
T_{31}	LLCT	58	3.11	399	0.013	2.74
T_{32}	$MLCT_{NHCp}$	62	3.13	397	0.013	2.73
T_{37}	$MLCT_{NHCp}$, LLCT<br>$MLCT_{NHCp}$	39<br>15	3.24	382	0.027	2.66
T_{54}	LLCT, $MLCT_{NHCp}$<br>LLCT, $MLCT_{NHCp}$	41<br>18	3.59	345	0.020	2.84
T_{55}	$MLCT_{NHCp}$	30	3.61	344	0.018	3.25
T_{56}	LLCT	55	3.63	341	0.019	2.83
T_{57}	$MLCT_{NHCp}$, LLCT	20	3.65	339	0.066	3.11
T_{58}	$ILCT_{BBI}$	22	3.66	338	0.333	2.68
T_{64}	LLCT	24	3.75	330	0.110	2.87

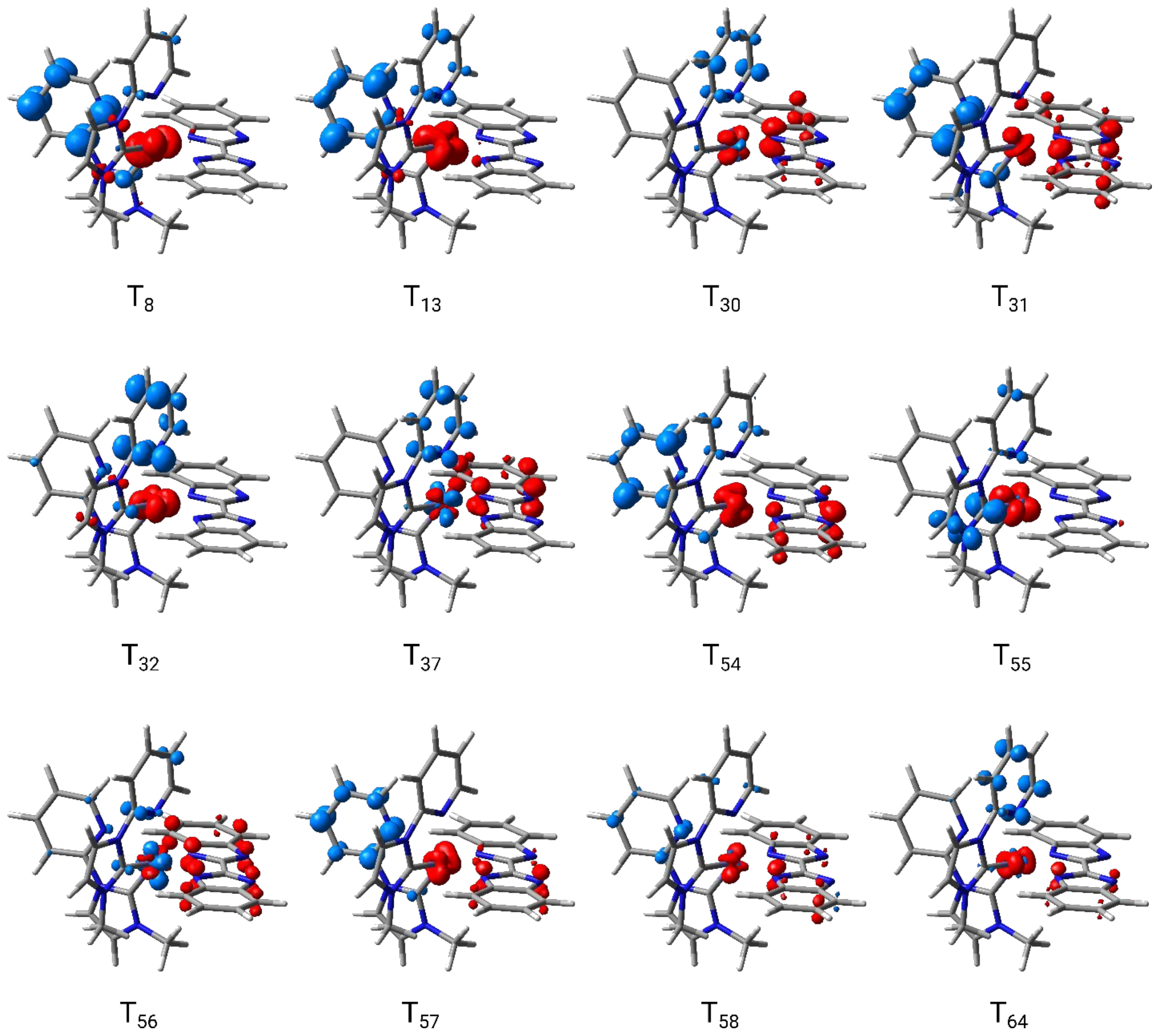

*Figure S 49: Charge density differences (CDDs) of prominent triplet-triplet excitations involved in the UV/vis absorption of **deprotonated C1 in DMF** within ^{3}MC equilibrated structure. Charge transfer takes place from red to blue.*

*Table S 21: Calculated vertical excitation energies (E^e), wavelengths (λ), oscillator strengths (f), spin contamination ($\langle s^2 \rangle$), and singly-excited configurations of the main excited quintet-quintet transitions involved in the initial absorption of **deprotonated C1 in DMF within ^{5}MC equilibrated geometry**.*

State	Transition Type	Weight / %	E^e / eV	λ / nm	f	s^2
Q_4	$MLCT_{NHCp}$	73	1.86	666	0.031	6.92
Q_8	$MLCT_{NHCp}$	48	2.09	594	0.017	6.74
	$MLCT_{NHCP}$	32				
Q_{21}	$LMCT_{BBI}$, LLCT	88	2.85	435	0.017	6.43
Q_{54}	LLCT	60	3.65	340	0.064	6.90
	LLCT	19				

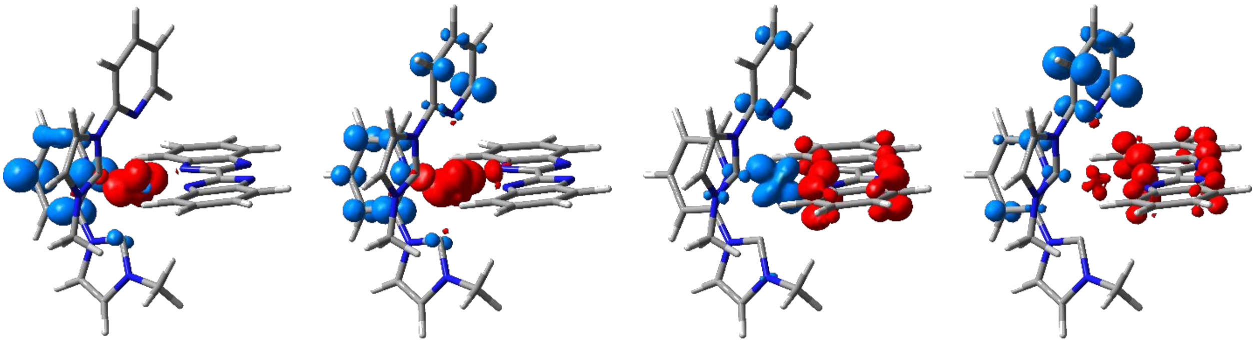

Q_4 Q_8 Q_{21} Q_{54}

Figure S 50: Charge density differences (CDDs) of prominent quintet-quintet excitations involved in the UV/vis absorption of ***deprotonated C1 in DMF*** *within ^{5}MC equilibrated structure. Charge transfer takes place from red to blue.*

Table S 22: Calculated vertical excitation energies (E^e), wavelengths (λ), oscillator strengths (f), spin contamination ($\langle s^2 \rangle$), and singly-excited configurations of the main excited triplet-triplet transitions involved in the initial absorption of ***protonated C1 in ACN within ^{3}MLCT equilibrated geometry****.*

State	Transition Type	Weight / %	E^e / eV	λ / nm	f	s^2
T_{12}	$ILCT_{BBI}$	85	2.14	579	0.181	2.04
T_{19}	$LMCT_{NHCp}$	76	2.59	478	0.020	2.12
T_{25}	LLCT, MC<br>LLCT, MC	25<br>17	2.86	434	0.022	2.47
T_{27}	LLCT<br>LLCT<br>$ILCT_{BBI}$	33<br>24<br>17	2.92	425	0.034	2.79
T_{28}	LLCT<br>$ILCT_{BBI}$<br>LLCT	22<br>19<br>13	2.95	420	0.051	2.68
T_{34}	LLCT, $MLCT_{NHCp}$	76	3.06	406	0.026	3.21
T_{37}	MC	13	3.18	390	0.023	2.31
T_{39}	LLCT	59	3.32	384	0.058	2.51
T_{40}	LLCT<br>$LMCT_{BBI}$	41<br>19	3.26	380	0.133	2.40
T_{41}	$LMCT_{BBI}$<br>LLCT	41<br>30	3.31	375	0.072	2.62
T_{42}	LLCT, MC	34	3.35	370	0.025	2.81
T_{43}	MLCT, LLCT, $ILCT_{BBI}$<br>MLCT, LLCT, $ILCT_{BBI}$	35<br>26	3.37	368	0.051	2.80

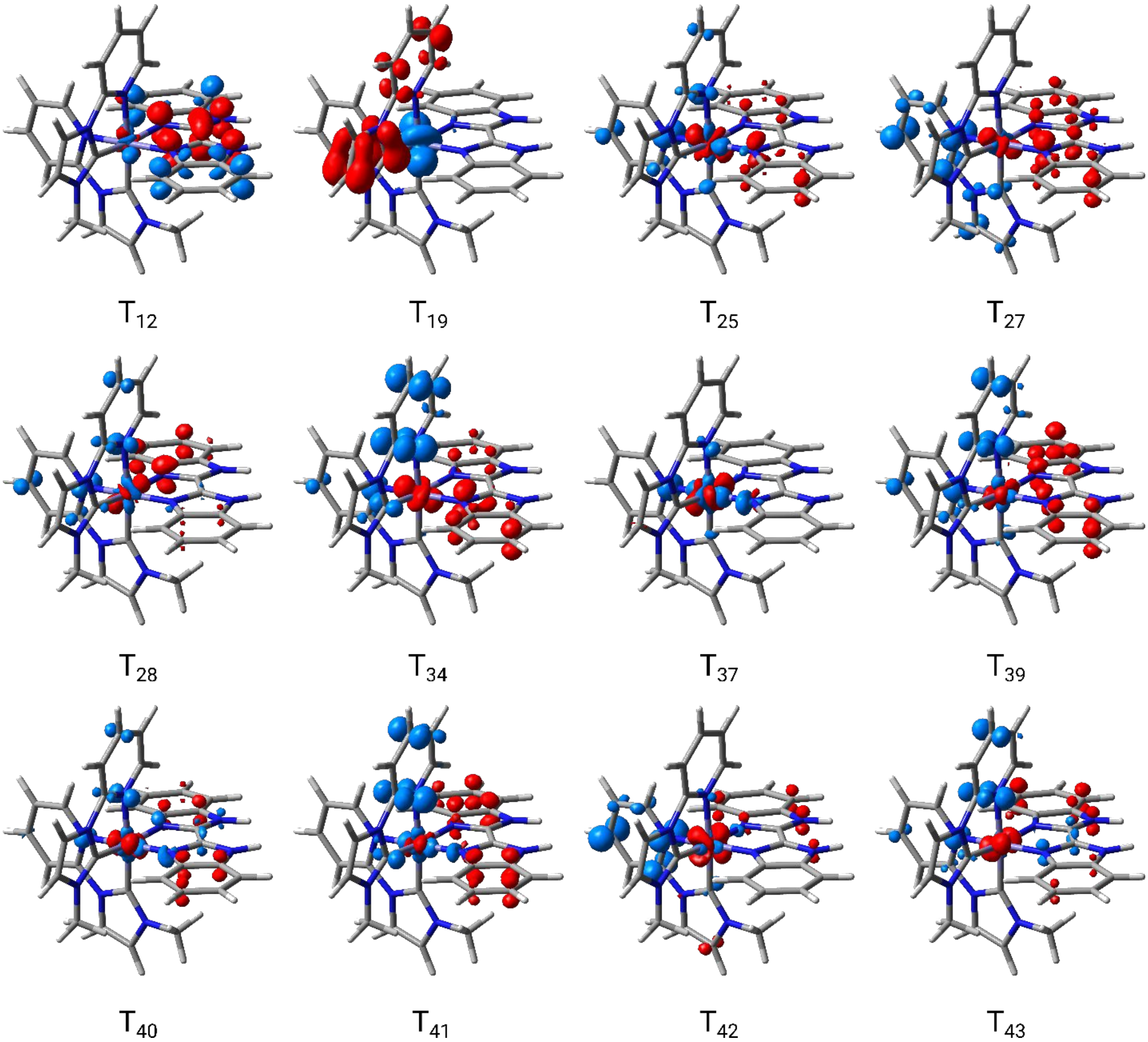

Figure S 51: Charge density differences (CDDs) of prominent triplet-triplet excitations involved in the UV/vis absorption of ***protonated C1 in ACN*** *within ^{3}MLCT equilibrated structure. Charge transfer takes place from red to blue.*

*Table S 23: Calculated vertical excitation energies (E^e), wavelengths (λ), oscillator strengths (f), spin contamination ($\langle s^2 \rangle$), and singly-excited configurations of the main excited triplet-triplet transitions involved in the initial absorption of **protonated C1 in ACN within ^{3}MC equilibrated geometry**.*

State	Transition Type	Weight / %	E^e / eV	λ / nm	f	s^2
T_6	$MLCT_{BBI}$, MC<br>$MLCT_{BBI}$	38<br>34	1.88	660	0.016	3.03
T_7	$ILCT_{NHCp}$, $MLCT_{NHCp}$<br>$MLCT_{NHCp}$	42<br>38	2.02	614	0.013	2.89
T_{13}	$ILCT_{NHCp}$, MC	55	2.38	521	0.013	3.21
T_{14}	$MLCT_{NHCp}$<br>$LMCT_{NHCp}$, MC	29<br>18	2.43	510	0.011	3.22
T_{28}	$ILCT_{NHCp}$	48	3.21	387	0.050	2.66
T_{29}	$ILCT_{NHCp}$, $MLCT_{NHCp}$	24	3.22	385	0.021	3.02
T_{35}	MC, $LMCT_{NHCp}$<br>$MLCT_{NHCp}$, LLCT	33<br>27	3.47	358	0.025	2.50
T_{36}	$ILCT_{BBI}$, $LMCT_{BBI}$<br>LLCT, $MLCT_{NHCp}$	18<br>16	3.51	363	0.161	2.42
T_{37}	LLCT, $ILCT_{NHCp}$	35	3.53	351	0.026	2.94
T_{40}	$ILCT_{BBI}$, LLCT, $LMCT_{BBI}$<br>$ILCT_{BBI}$, $LMCT_{BBI}$	18<br>16	3.59	346	0.224	2.34
T_{41}	$ILCT_{BBI}$, $LMCT_{BBI}$	14	3.60	344	0.200	2.56
T_{44}	LLCT<br>$LMCT_{NHCp}$, LLCT	39<br>21	3.69	336	0.043	2.83

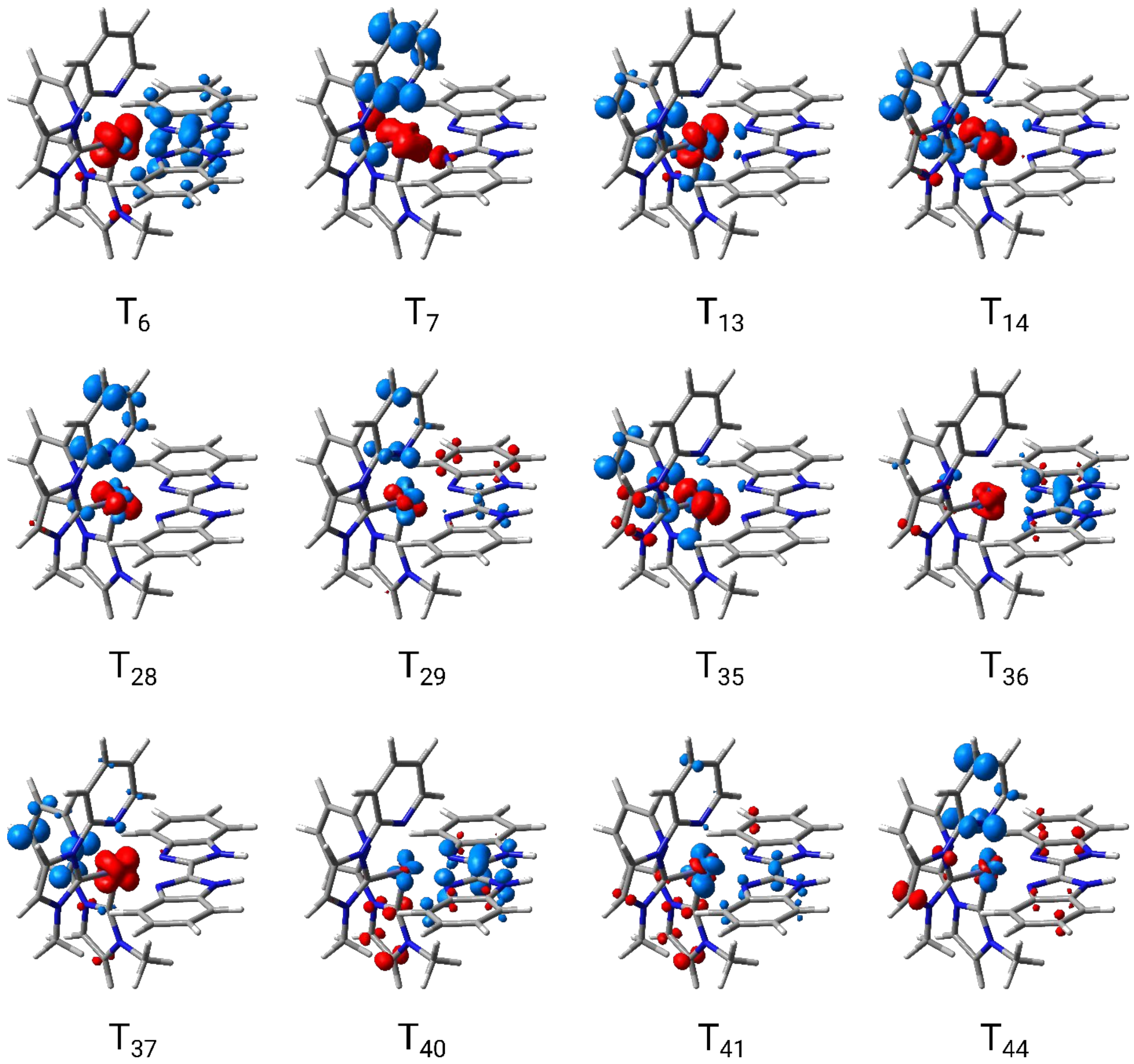

*Figure S 52: Charge density differences (CDDs) of prominent triplet-triplet excitations involved in the UV/vis absorption of **protonated C1 in ACN** within ^{3}MC equilibrated structure. Charge transfer takes place from red to blue.*

*Table S 24: Calculated vertical excitation energies (E^e), wavelengths (λ), oscillator strengths (f), spin contamination ($\langle s^2 \rangle$), and singly-excited configurations of the main excited quintet-quintet transitions involved in the initial absorption of **protonated C1 in ACN within ^{5}MC equilibrated geometry**.*

State	Transition Type	Weight / %	E^e / eV	λ / nm	f	s^2
Q_5	$MLCT_{BBI}$	97	1.86	667	0.011	6.22
Q_6	$MLCT_{NHCp}$	34	1.98	625	0.017	6.43
	$MLCT_{BBI}$, MC	29				
Q_{10}	$MLCT_{NHCp}$	63	2.45	507	0.021	6.42
Q_{19}	$LMCT_{BBI}$, $ILCT_{BBI}$	32	3.02	411	0.048	6.89
	$ILCT_{BBI}$, $LMCT_{BBI}$	19				
	$ILCT_{BBI}$	16				
Q_{29}	LLCT, $LMCT_{BBI}$	26	3.49	355	0.024	6.05
	$LMCT_{BBI}$, $ILCT_{BBI}$	21				
Q_{32}	$ILCT_{BBI}$, $LMCT_{BBI}$	27	3.60	345	0.398	6.32
	$LMCT_{BBI}$	23				
	$ILCT_{BBI}$	19				
Q_{33}	$LMCT_{BBI}$	25	3.60	344	0.069	6.90
Q_{34}	$MLCT_{BBI}$	63	3.61	343	0.135	6.85
Q_{40}	$LMCT_{BBI}$, $ILCT_{BBI}$	27	3.72	333	0.027	7.08
Q_{41}	$MLCT_{BBI}$	32	3.73	332	0.028	6.97
	$LMCT_{BBI}$, $ILCT_{BBI}$	16				

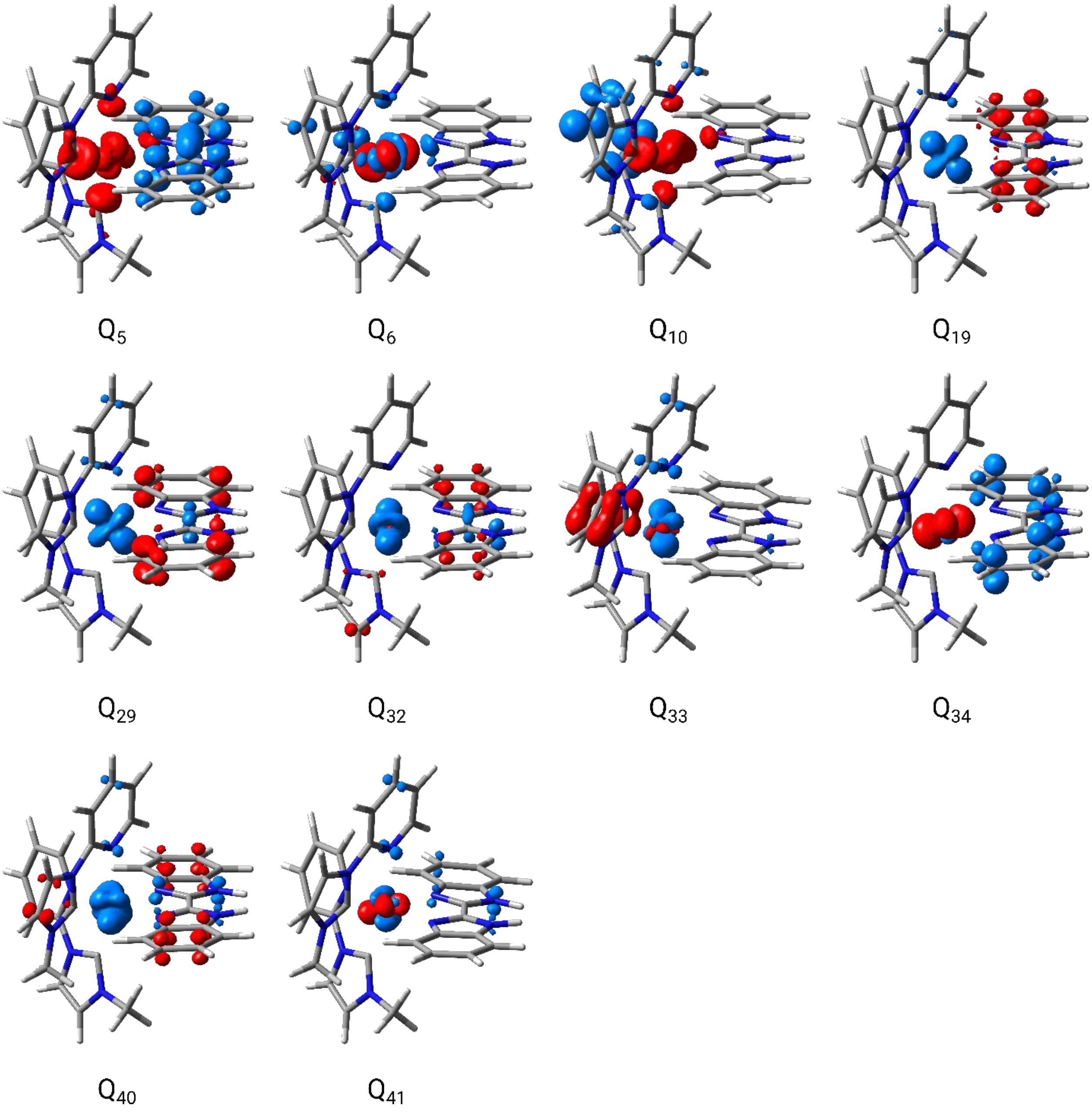

Figure S 53: Charge density differences (CDDs) of prominent quintet-quintet excitations involved in the UV/vis absorption of ***protonated C1 in ACN*** *within* ^{5}*MC equilibrated structure. Charge transfer takes place from red to blue.*

*Table S 25: Calculated vertical excitation energies (E^e), wavelengths (λ), oscillator strengths (f), spin contamination ($\langle s^2 \rangle$), and singly-excited configurations of the main excited triplet-triplet transitions involved in the initial absorption of **deprotonated C2 in DMF within ³MLCT equilibrated geometry**.*

State	Transition Type	Weight / %	E^e / eV	λ / nm	f	s^2
T_{10}	$ILCT_{NHCp}$	95	1.76	705	0.052	2.06
T_{11}	$LMCT_{BBI}$, MC	64	1.89	656	0.012	2.06
	$LMCT_{BBI}$, MC	34				
T_{13}	$LMCT_{BBI}$, MC	72	2.03	610	0.013	2.08
	$LMCT_{BBI}$, MC	25				
T_{18}	LLCT, $ILCT_{NHCp}$	70	2.41	515	0.030	2.03
	LLCT, $ILCT_{NHCp}$	29				
T_{51}	$ILCT_{BBI}$	30	3.47	357	0.145	2.68
	LLCT	20				
T_{53}	$LMCT_{BBI}$, LLCT	37	3.55	349	0.094	2.99
T_{59}	$LLCT_{BBI}$, ILCT	20	3.61	343	0.043	2.67
	LLCT	16				
	$LMCT_{BBI}$, LLCT	15				
T_{65}	$LMCT_{BBI}$, LLCT	14	3.66	338	0.250	2.72
	$MLCT_{BBI}$, LLCT, $ILCT_{BBI}$	13				

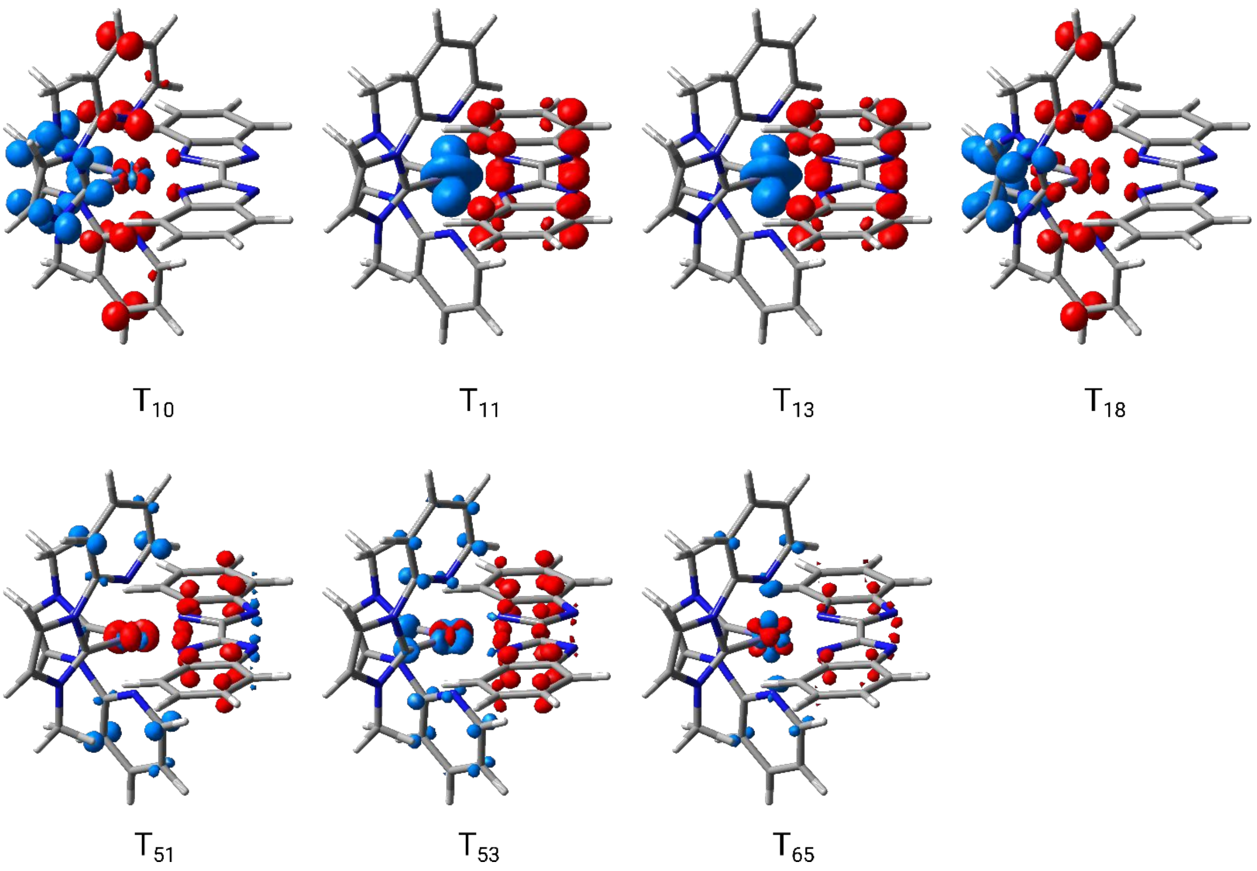

*Figure S 54: Charge density differences (CDDs) of prominent triplet-triplet excitations involved in the UV/vis absorption of **deprotonated C2 in DMF** within ³MLCT equilibrated structure. Charge transfer takes place from red to blue.*

*Table S 26: Calculated vertical excitation energies (E^e), wavelengths (λ), oscillator strengths (f), spin contamination ($\langle s^2 \rangle$), and singly-excited configurations of the main excited triplet-triplet transitions involved in the initial absorption of **deprotonated C2 in DMF within ^{3}MC equilibrated geometry**.*

State	Transition Type	Weight / %	E^e / eV	λ / nm	f	s^2
T_4	$MLCT_{NHCp}$, MC	54	1.79	694	0.023	2.89
T_9	$MLCT_{NHCp}$, ILCT<br>$MLCT_{NHCp}$	62<br>27	2.16	574	0.011	3.12
T_{10}	$MLCT_{NHCp}$<br>$MLCT_{NHCp}$, ILCT	52<br>30	2.16	573	0.016	3.04
T_{17}	$MLCT_{NHCP}$, ILCT	98	2.48	500	0.011	3.07
T_{23}	LLCT, $MLCT_{NHCp}$	91	2.78	445	0.027	2.89
T_{30}	$MLCT_{NHCp}$, LLCT, $ILCT_{BBI}$	75	3.07	403	0.014	3.13
T_{39}	$ILCT_{BBI}$, LLCT<br>$ILCT_{BBI}$, LLCT	53<br>25	3.27	380	0.017	2.58
T_{41}	LLCT, $ILCT_{BBI}$<br>LLCT, $ILCT_{BBI}$	59<br>23	3.32	373	0.017	3.40
T_{49}	MC, $LMCT_{NHCp}$	29	3.52	352	0.020	2.31
T_{56}	$ILCT_{BBI}$, LLCT<br>$ILCT_{BBI}$, LLCT	34<br>26	3.62	342	0.542	2.43
T_{60}	LLCT, $MLCT_{NHCp}$	71	3.67	338	0.070	3.38

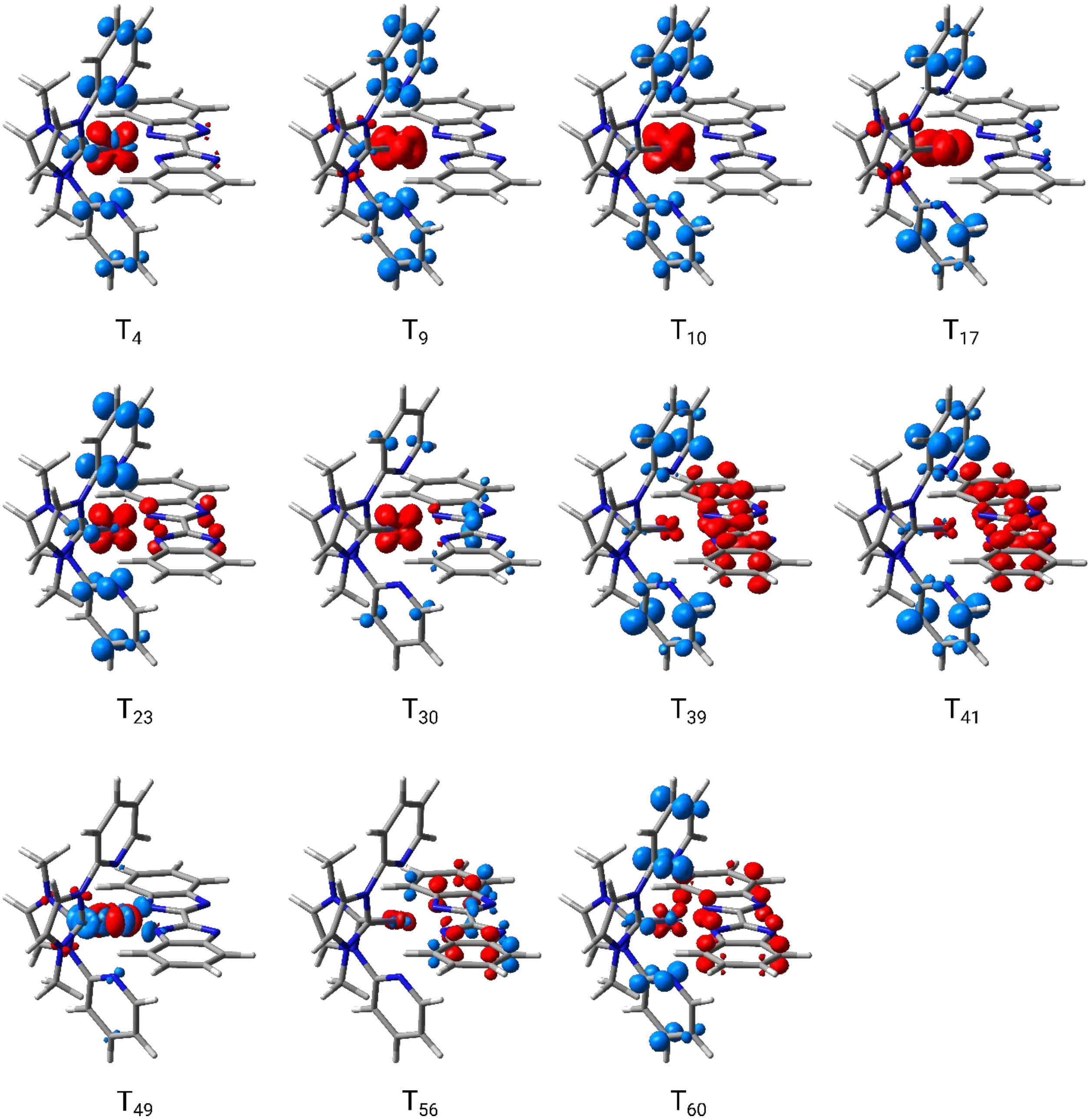

*Figure S 55: Charge density differences (CDDs) of prominent triplet-triplet excitations involved in the UV/vis absorption of **deprotonated C2 in DMF** within ^{3}MC equilibrated structure. Charge transfer takes place from red to blue.*

Table S 27: Calculated vertical excitation energies (E^e), wavelengths (λ), oscillator strengths (f), spin contamination ($\langle s^2 \rangle$), and singly-excited configurations of the main excited quintet-quintet transitions involved in the initial absorption of ***deprotonated C2 in DMF within ^{5}MC equilibrated geometry****.*

State	Transition Type	Weight / %	E^e / eV	λ / nm	f	s^2
Q_6	$MLCT_{NHCp}$, MC	54	1.90	653	0.060	6.98
	$MLCT_{NHCp}$, MC	23				
	$MLCT_{NHCp}$, MC	18				
Q_{17}	LLCT, $LMCT_{BBI}$	87	2.55	486	0.009	6.47
Q_{18}	LLCT, $LMCT_{BBI}$	93	2.71	458	0.014	6.48
Q_{22}	$LMCT_{BBI}$	88	2.85	435	0.013	6.19
Q_{51}	LLCT, $LMCT_{BBI}$	89	3.56	348	0.012	6.97
Q_{56}	$MLCT_{NHCp}$	20	3.63	341	0.107	6.50
	ILCT, $MLCT_{BBI}$	18				
	LLCT, $LMCT_{BBI}$, $ILCT_{NHCp}$	16				
Q_{59}	$LMCT_{BBI}$	69	3.66	339	0.140	6.23
Q_{61}	LLCT	28	3.67	338	0.228	6.43
	$LMCT_{BBI}$	27				
Q_{63}	$MLCT_{NHCp}$	29	3.70	335	0.297	6.76

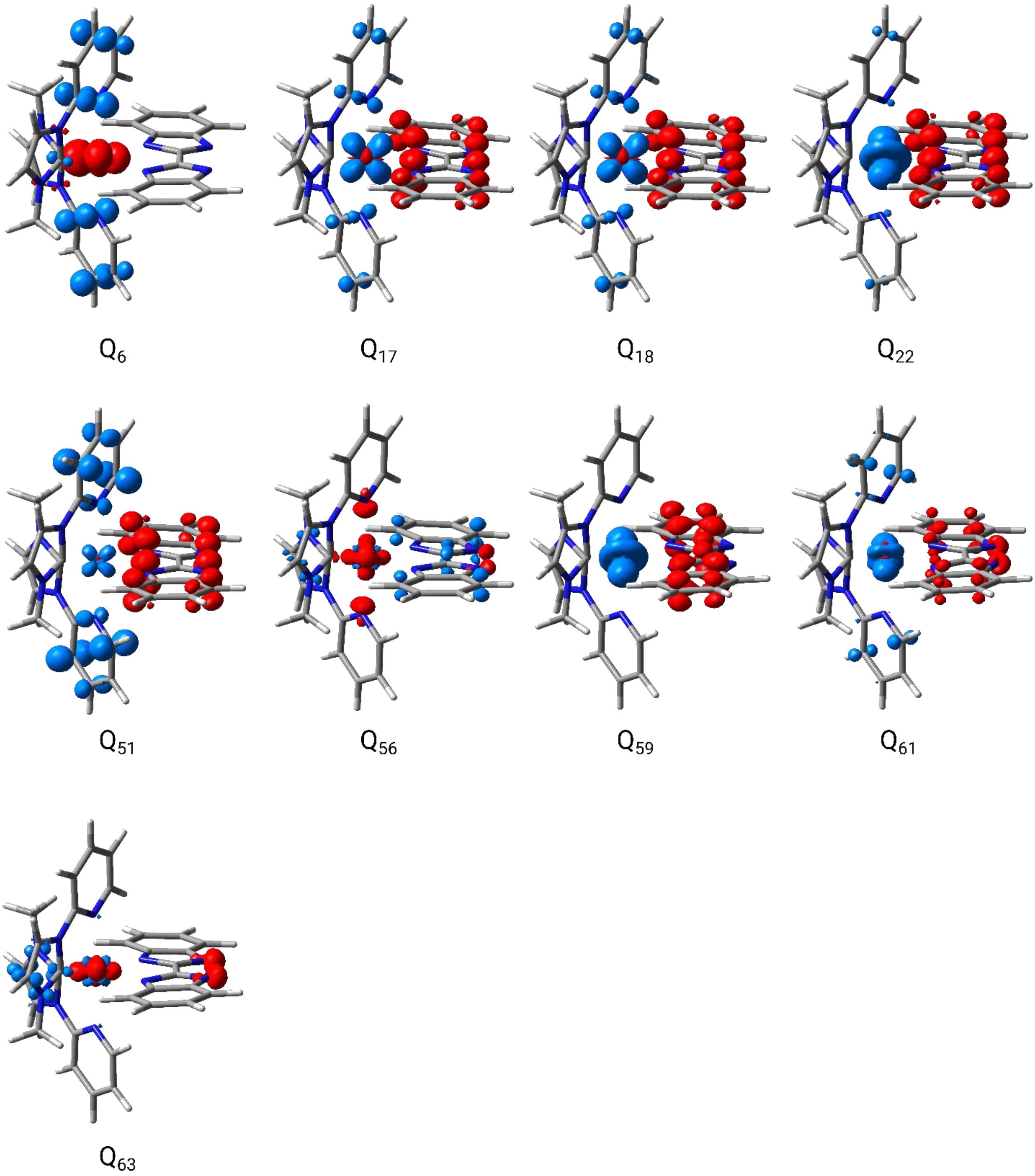

*Figure S 56: Charge density differences (CDDs) of prominent quintet-quintet excitations involved in the UV/vis absorption of **deprotonated C2 in DMF** within ^{5}MC equilibrated structure. Charge transfer takes place from red to blue.*

*Table S 28: Calculated vertical excitation energies (E^e), wavelengths (λ), oscillator strengths (f), spin contamination ($\langle s^2 \rangle$), and singly-excited configurations of the main excited triplet-triplet transitions involved in the initial absorption of **protonated C2 in ACN within ^{3}MLCT equilibrated geometry**.*

State	Transition Type	Weight / %	E^e / eV	λ / nm	f	s^2
T_{13}	$ILCT_{BBI}$	88	2.13	582	0.185	2.03
T_{16}	$LMCT_{NHCp}$	96	2.28	543	0.021	2.05
T_{20}	$ILCT_{BBI}$<br>$LMCT_{BBI}$	70<br>26	2.57	483	0.027	2.08
T_{23}	$ILCT_{BBI}$, LLCT	56	2.81	440	0.043	2.79
T_{27}	$ILCT_{BBI}$, LLCT<br>LLCT<br>$ILCT_{BBI}$, LLCT	23<br>19<br>14	2.94	421	0.116	2.99
T_{32}	$ILCT_{BBI}$, LLCT	62	3.06	406	0.027	3.44
T_{36}	$ILCT_{BBI}$, LLCT, MLCT<br>$ILCT_{BBI}$, LLCT, MLCT	40<br>17	3.15	394	0.030	2.56
T_{37}	$LLCT_{BBI}$<br>$ILCT_{BBI}$, LLCT	69<br>14	3.18	390	0.100	2.52
T_{40}	$ILCT_{BBI}$, LLCT, MC	76	3.28	378	0.186	2.37
T_{42}	ILCT, LLCT, MC<br>$ILCT_{BBI}$, LLCT, MC	48<br>28	3.38	367	0.048	2.60
T_{44}	$ILCT_{BBI}$, LLCT, MC<br>ILCT, LLCT, MC	40<br>17	3.45	359	0.040	2.97

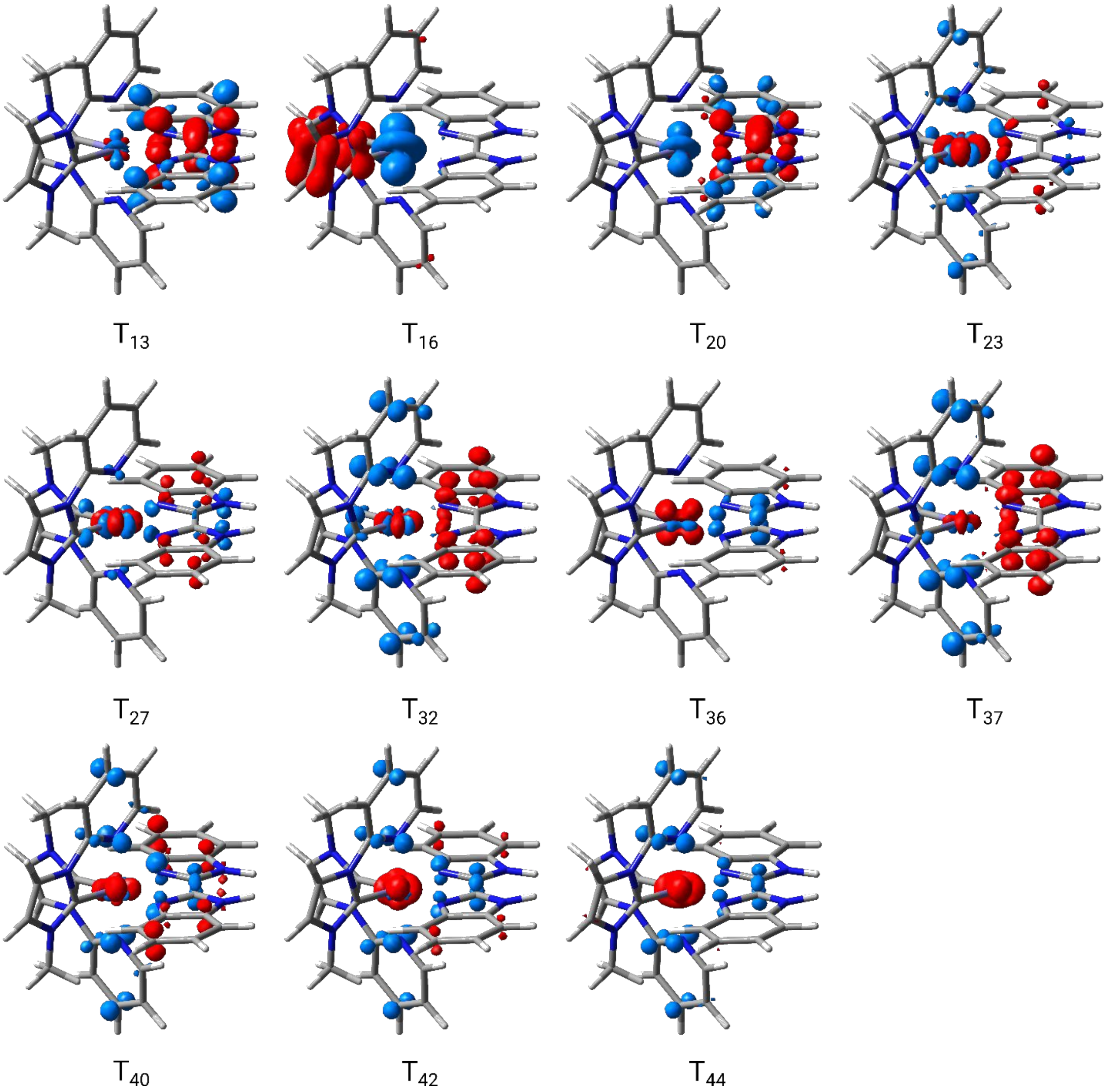

Figure S 57: Charge density differences (CDDs) of prominent triplet-triplet excitations involved in the UV/vis absorption of ***protonated C2 in ACN*** *within ^{3}MLCT equilibrated structure. Charge transfer takes place from red to blue.*

Table S 29: Calculated vertical excitation energies (E^e), wavelengths (λ), oscillator strengths (f), spin contamination ($\langle s^2 \rangle$), and singly-excited configurations of the main excited triplet-triplet transitions involved in the initial absorption of ***protonated C2 in ACN within ³MC equilibrated geometry***.

State	Transition Type	Weight / %	E^e / eV	λ / nm	f	s^2
T_{10}	$MLCT_{NHCp}$, MC	81	2.17	570	0.021	3.03
T_{13}	$MLCT_{NHCp}$, $ILCT_{NHCp}$	75	2.41	514	0.024	3.35
T_{20}	$MLCT_{BBI}$<br>MC, $LMCT_{NHCp}$	56<br>21	2.68	462	0.015	2.64
T_{21}	MC, $LMCT_{NHCp}$<br>$MLCT_{NHCp}$, $ILCT_{NHCp}$<br>MC, $ILCT_{NHCp}$	24<br>18<br>17	2.72	456	0.010	3.58
T_{25}	$MLCT_{NHCp}$, $ILCT_{NHCp}$	76	2.89	428	0.010	3.14
T_{30}	$MLCT_{NHCp}$	90	3.29	377	0.028	2.89
T_{34}	$ILCT_{BBI}$<br>$ILCT_{BBI}$	43<br>34	3.44	360	0.501	2.23
T_{41}	$LMCT_{NHCp}$	47	3.61	344	0.020	2.29
T_{43}	$LMCT_{NHCp}$, $ILCT_{NHCp}$	32	3.66	339	0.070	2.52
T_{45}	$MLCT_{BBI}$	35	3.69	336	0.090	2.50

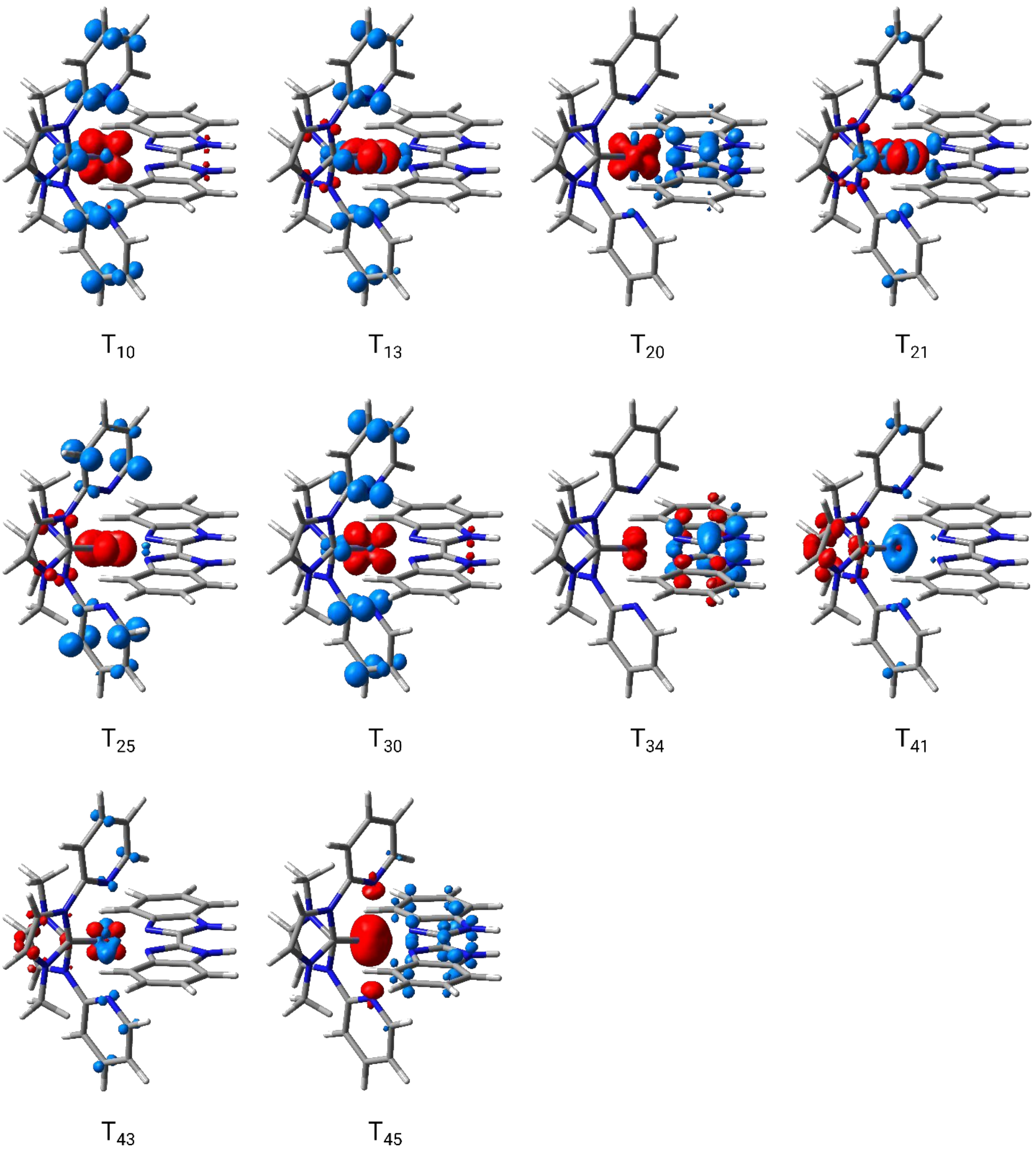

Figure S 58: Charge density differences (CDDs) of prominent triplet-triplet excitations involved in the UV/vis absorption of ***protonated C2 in ACN*** *within ^{3}MC equilibrated structure. Charge transfer takes place from red to blue.*

*Table S 30: Calculated vertical excitation energies (E^e), wavelengths (λ), oscillator strengths (f), spin contamination (<$\langle s^2 \rangle$>), and singly-excited configurations of the main excited quintet-quintet transitions involved in the initial absorption of **protonated C2 in ACN within ⁵MC equilibrated geometry**.*

State	Transition Type	Weight / %	E^e / eV	λ / nm	f	s^2
Q_6	$MLCT_{NHCp}$, MC	47	2.03	611	0.026	6.43
	MC, $MLCT_{BBI}$	29				
Q_9	MC, $MLCT_{BBI}$	31	2.35	528	0.030	6.62
	$MLCT_{NHCp}$, MC	26				
	$MLCT_{NHCp}$	15				
Q_{17}	$LMCT_{BBI}$, $ILCT_{BBI}$	36	2.97	418	0.032	6.84
	$ILCT_{BBI}$, $LMCT_{BBI}$	28				
	$ILCT_{BBI}$	23				
Q_{34}	$ILCT_{BBI}$, $LMCT_{BBI}$	31	3.58	346	0.419	6.16
	$LMCT_{NHCp,BBI}$	31				
	$ILCT_{BBI}$	22				
Q_{35}	$LMCT_{NHCp,BBI}$	30	3.63	342	0.326	6.23
	$LMCT_{NHCp,BBI}$	29				
	$ILCT_{BBI}$, $LMCT_{BBI}$	19				
	$ILCT_{BBI}$	15				
Q_{37}	ILCT	29	3.66	339	0.040	7.41

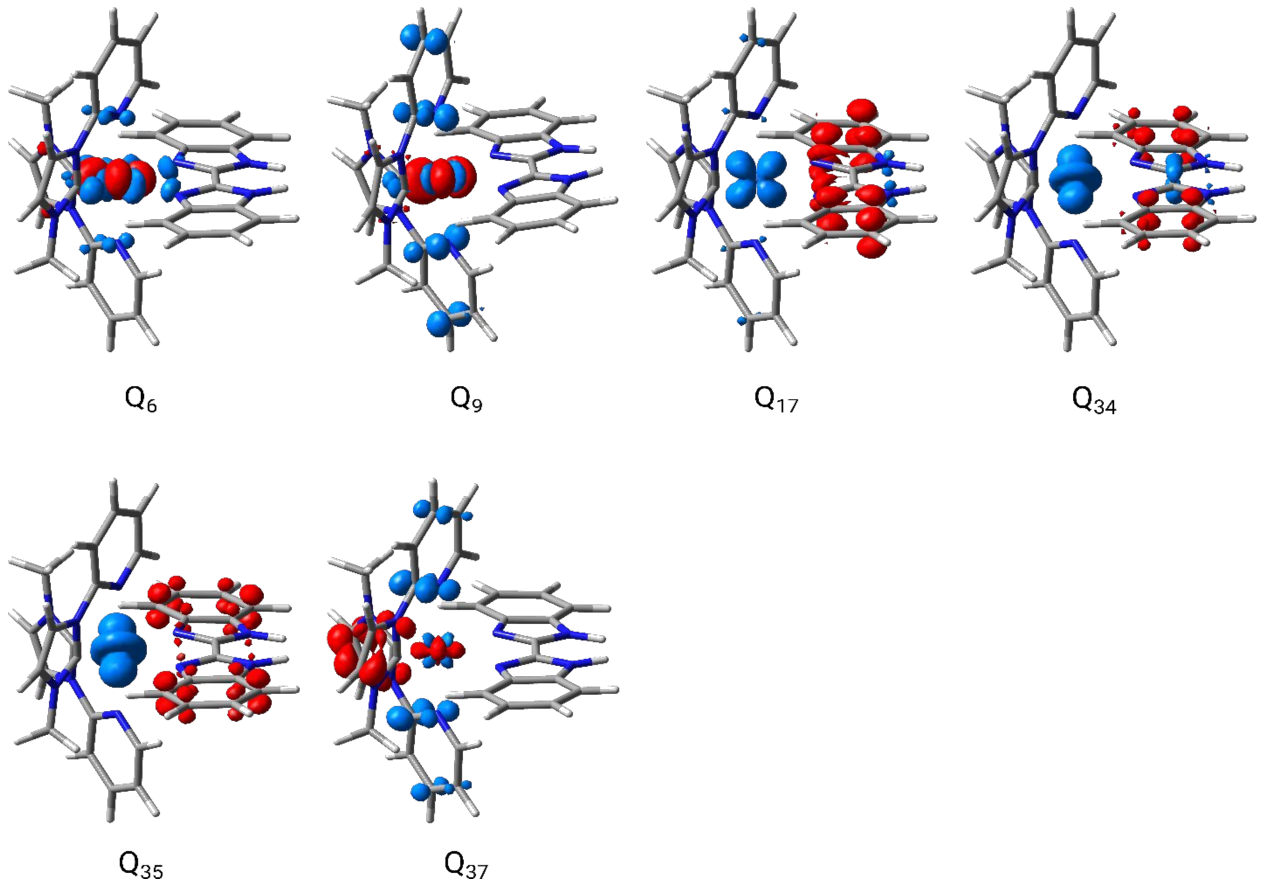

*Figure S 59: Charge density differences (CDDs) of prominent quintet-quintet excitations involved in the UV/vis absorption of **protonated C2 in ACN** within ⁵MC equilibrated structure. Charge transfer takes place from red to blue.*

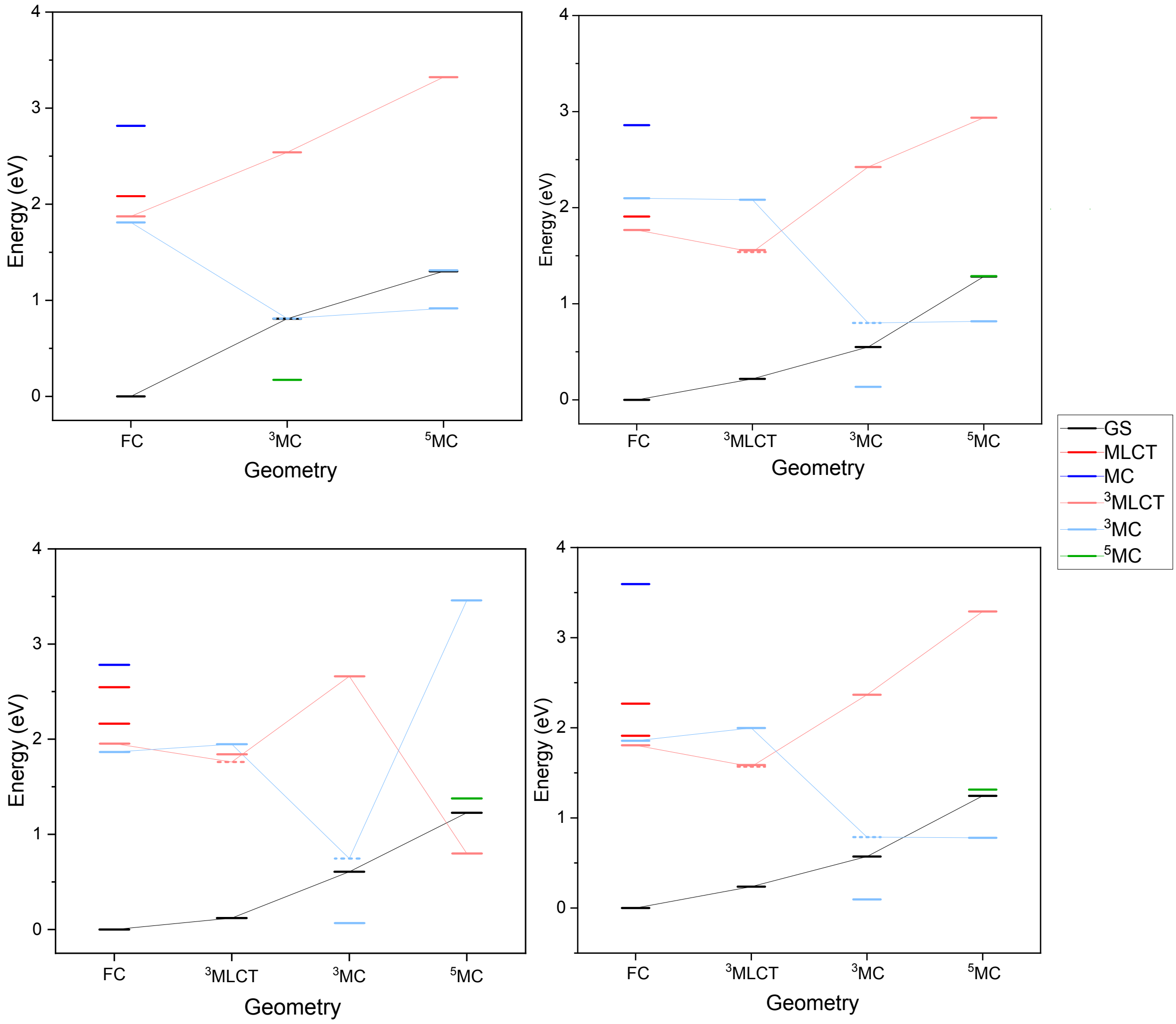

Figure S 60. Comparison of calculated ground (black), lowest $^{1/3/5}$MC (blue/sky-blue/green), and $^{1/3}$MLCT (red/pink) states in equilibrated FC, ^{3}MC, ^{5}MC, and ^{3}MLCT geometries for C1^{deprot} (A), C1^{prot} (B), C2^{deprot} (C), C2^{prot} (D). DMF and ACN were utilized as solvents for all deprotonated and protonated species, respectively. Bold bars label (restricted) DFT and TDDFT energies as obtained by singlet-singlet and singlet-triplet calculations, respectively. Dashed bars represent unrestricted DFT energies of the lowest energy triplet state..

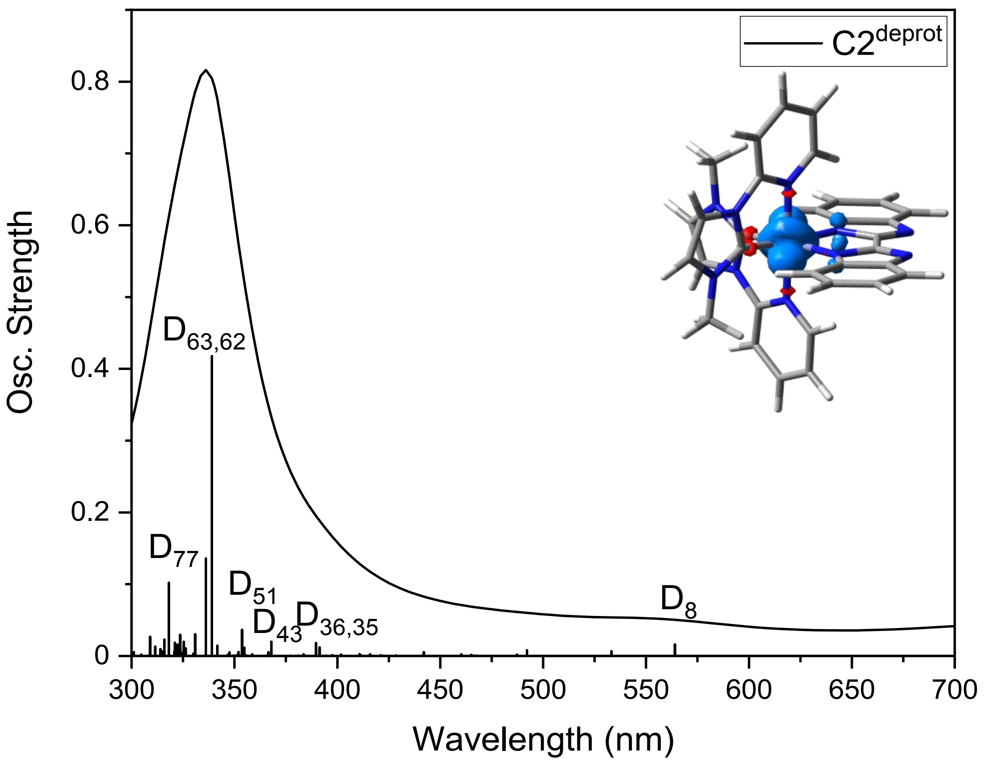

*Figure S 61. Simulated electronic absorption spectrum of oxidized **C2^{deprot}** in ACN; key electronic doublet excitations in the visible region are labeled. The transitions were broadened by Lorentzian functions with a full width at half maximum of 0.2 eV. Spin density indicates a formal Fe(III) doublet ground state.*

*Table S 31. Calculated vertical excitation energies (E^e), wavelengths (λ), oscillator strengths (f) and singly-excited configurations of the main excited doublet-doublet transitions (see spin contamination) involved in the absorption of oxidized **C2**^{deprot} in ACN.*

State	Transition Type	Weight / %	E^e / eV	λ / nm	f	s^2
D_8	$LMCT_{BBI}$, MC	69	2.20	564	0.016	0.80
	$LMCT_{BBI}$, MC	30				
D_{35}	LLCT	51	3.17	391	0.012	1.96
	ILCT	11				
D_{36}	LLCT	57	3.18	390	0.018	1.45
	LLCT	12				
D_{43}	ILCT, LLCT, MC	75	3.37	368	0.020	1.92
D_{51}	$LMCT_{BBI}$	44	3.51	354	0.036	1.59
	LLCT	11				
D_{62}	$ILCT_{BBI}$	29	3.66	339	0.418	1.09
	$ILCT_{BBI}$	24				
	$LMCT_{BBI}$	10				
D_{63}	LLCT, MC	14	3.69	336	0.136	1.74
	$ILCT_{NHCpy}$	9				
D_{77}	$ILCT_{BBI}$	33	3.90	318	0.102	1.38
	$ILCT_{BBI}$	15				
	$MLCT_{BBI}$	9				

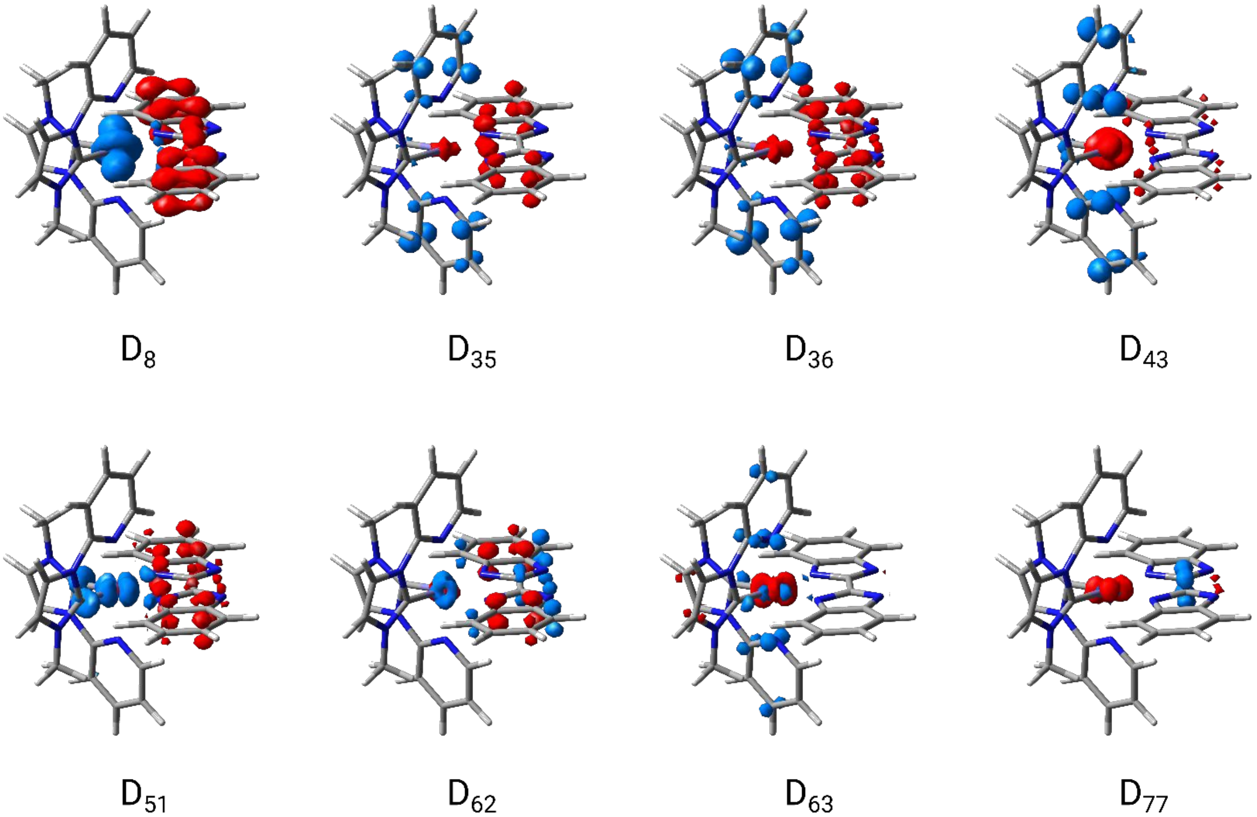

Figure S 62. Charge density differences (CDDs) of prominent doublet-doublet excitations involved in the UV/vis absorption of oxidized ***C2***^{deprot} *in ACN within the equilibrated doublet ground state geometry. Charge transfer takes place from red to blue.*

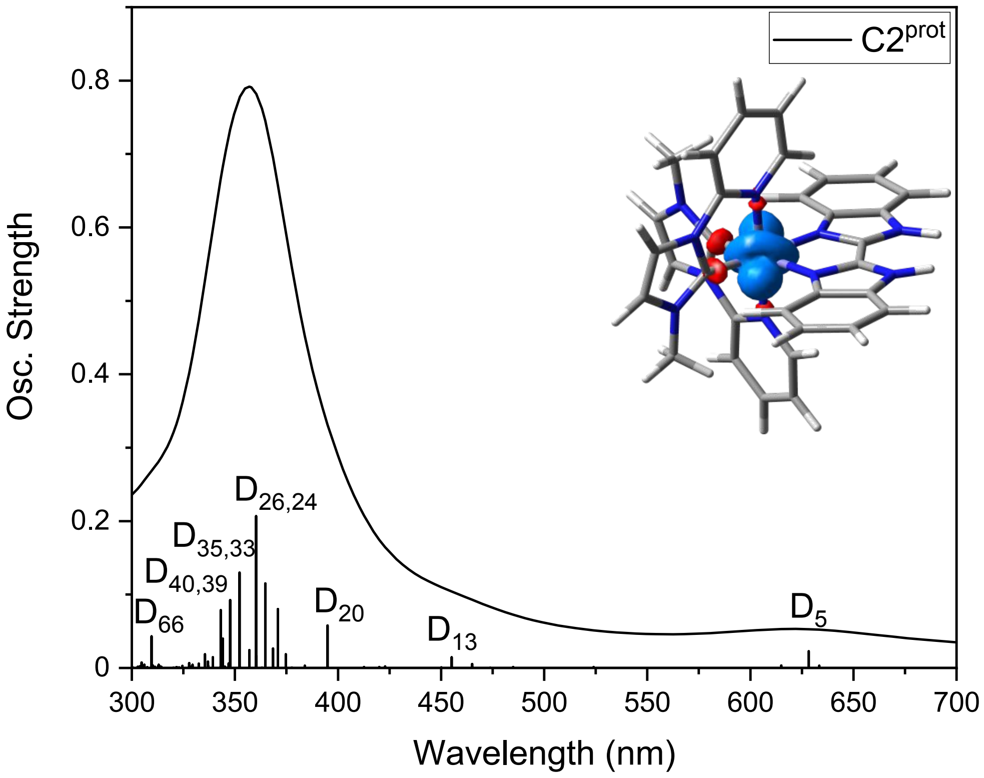

Figure S 63. Simulated electronic absorption spectrum of oxidized ***C2^{prot}*** *in ACN; key electronic doublet excitations in the visible region are labeled. The transitions were broadened by Lorentzian functions with a full width at half maximum of 0.2 eV. Spin density indicates a formal Fe(III) doublet ground state.*

*Table S 32. Calculated vertical excitation energies (E^e), wavelengths (λ), oscillator strengths (f) and singly-excited configurations of the main excited doublet-doublet transitions (see spin contamination) involved in the absorption of oxidized **C2**^{prot} in ACN.*

State	Transition Type	Weight / %	E^e / eV	λ / nm	*f*	s^2
D_5	$LMCT_{NHCpy}$	98	1.97	628	0.023	0.80
D_{13}	$LMCT_{NHCpy}$	80	2.72	455	0.014	0.86
D_{20}	$ILCT_{BBI}$	32	3.14	395	0.058	1.45
	$ILCT_{BBI}$, $MLCT_{BBI}$	16				
	$ILCT_{BBI}$	15				
	$ILCT_{BBI}$	13				
	$ILCT_{BBI}$	12				
D_{24}	LLCT, $LMCT_{BBI}$	33	3.34	371	0.080	1.40
	LLCT	12				
	$ILCT_{NHCpy}$	12				
D_{26}	$LMCT_{BBI}$	72	3.40	365	0.115	0.86
D_{33}	LLCT, MC	28	3.52	352	0.130	1.79
	LLCT	15				
D_{35}	$LMCT_{NHCpy}$	26	3.57	348	0.092	1.46
	LLCT	18				
	LLCT, MC	16				
D_{39}	$ILCT_{BBI}$	37	3.60	344	0.040	0.99
	$ILCT_{BBI}$	26				
	$MLCT_{NHCpy}$	14				
D_{40}	$MLCT_{NHCpy}$	51	3.61	343	0.079	1.71
	LLCT, MC	10				
D_{66}	$ILCT_{NHCpy}$	33	4.00	310	0.043	1.15
	$ILCT_{NHCpy}$	26				
	LLCT	14				

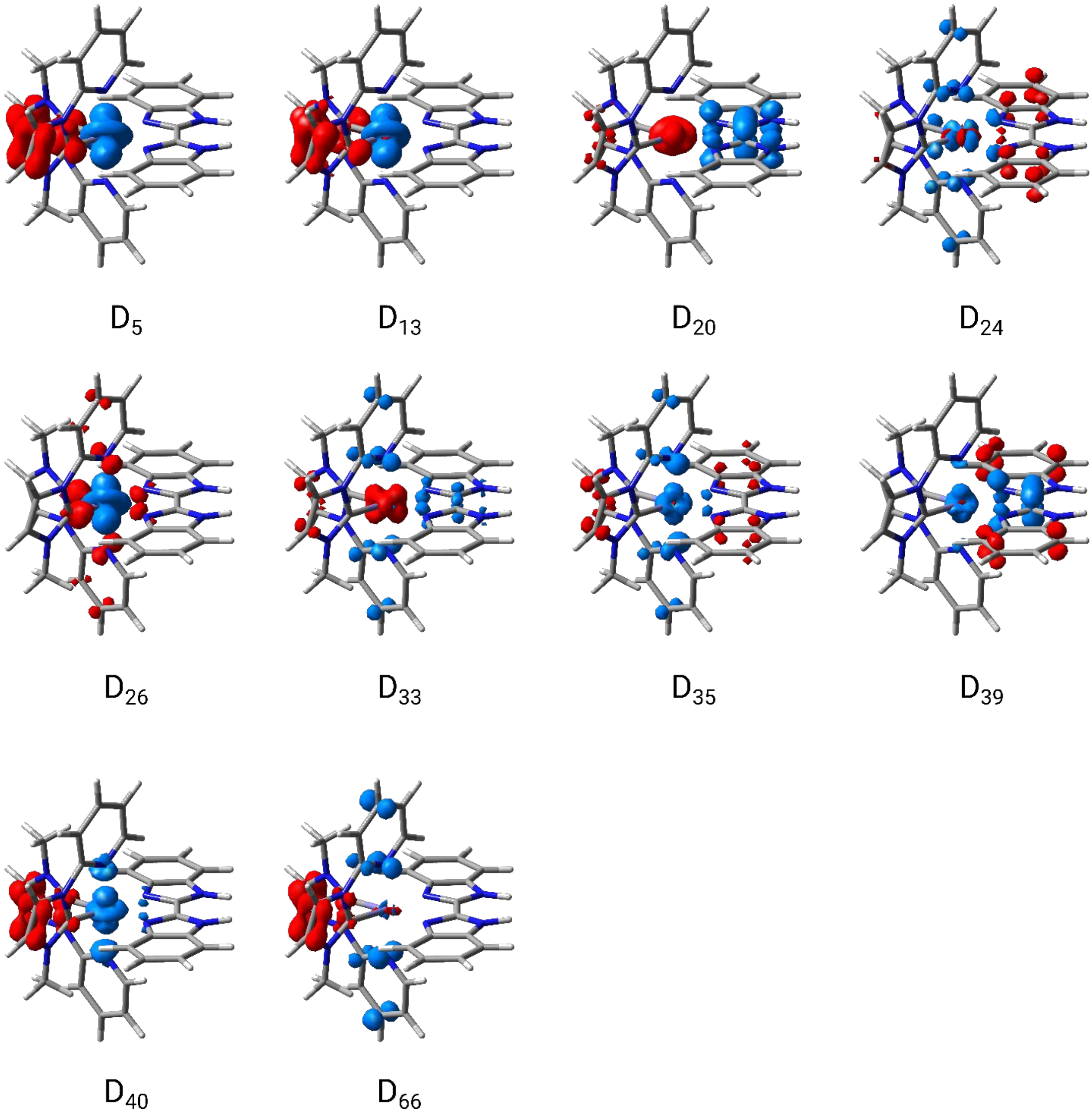

*Figure S 64. Charge density differences (CDDs) of prominent doublet-doublet excitations involved in the UV/vis absorption of protonated oxidized **C2**^{prot} in ACN within the equilibrated doublet ground state geometry. Charge transfer takes place from red to blue.*

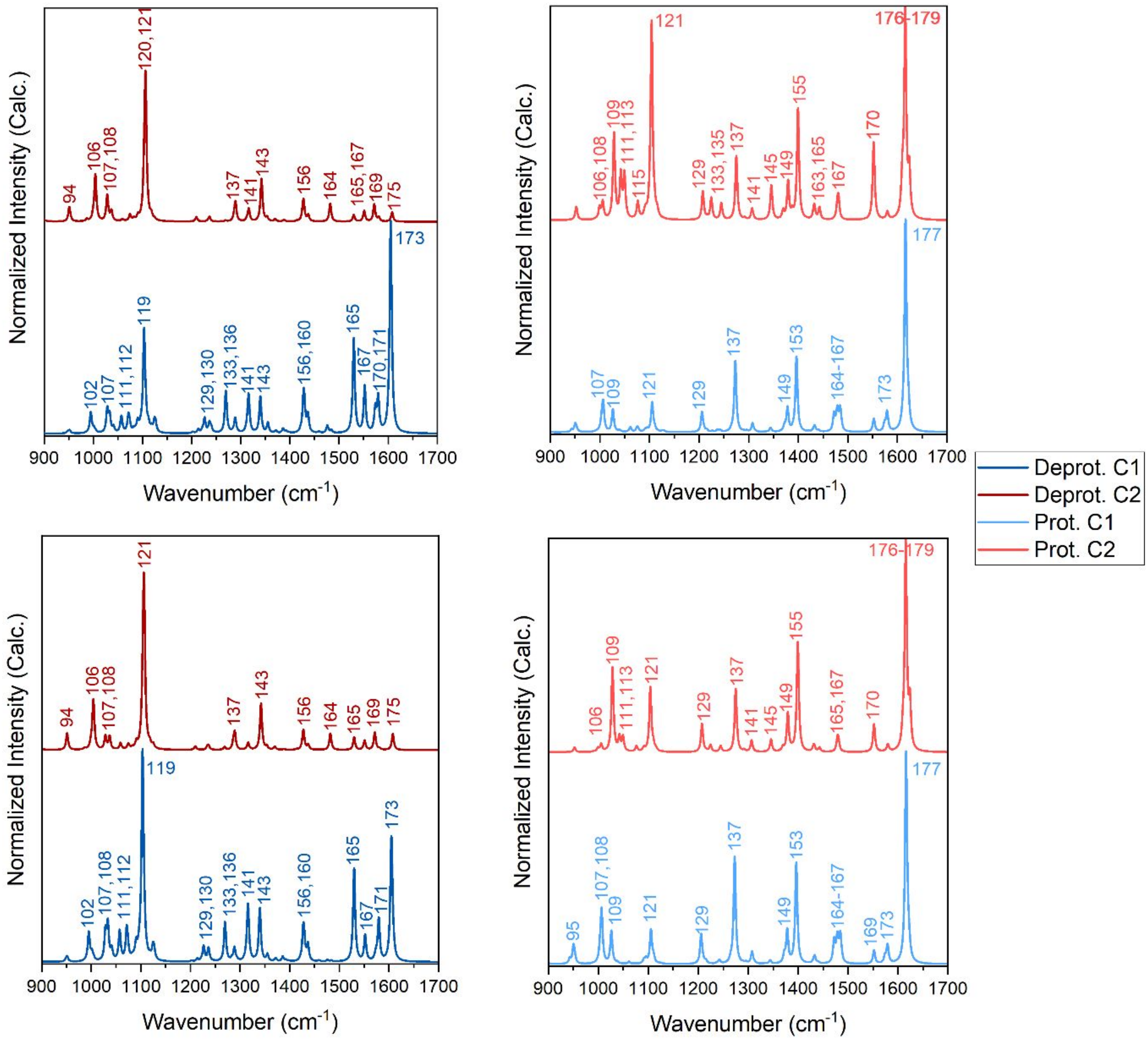

*Figure S 65: Simulated resonance-Raman spectra of **C1** (blue) and **C2** (red) in deprotonated and protonated states at the excitation wavelengths of 473 (top) and 532 (bottom) nm; see Tables S 33-S 36 for an in-depth assignment of the underlying vibrational normal modes.*

*Table S 33: Calculated resonance Raman active vibrational modes of deprotonated **C1** at excitation wavelengths of 473 and 532 nm (unscaled and scaled by the factor of 0.98). Modes with relative Raman intensities ≥ 0.05 are given.*

			**C1^{deprot}**		
Mode	Character	ν / cm^{-1}	ν_{scaled} / cm^{-1}	Rel. intensity (473 nm)	Rel. intensity (532 nm)
102	NHCp	1015.2	994.9	0.10	0.14
107	BBI/NHCp	1049.3	1028.3	0.11	0.13
108	NHCp	1054.3	1033.2	0.08	0.17
109	NHCp	1062.3	1041.0	0.03	0.05
111	NHCp	1078.7	1057.1	0.07	0.14
112	NHCp	1093.2	1071.3	0.08	0.15
116	NHCp/BBI	1112.5	1090.2	0.05	0.07
119	NHCp/BBI	1125.7	1103.2	0.49	1.00
120	BBI/NHCp	1129.0	1106.4	0.05	0.08
124	NHCp	1147.2	1124.2	0.04	0.06
129	NHCp	1251.4	1226.4	0.07	0.07
130	NHCp	1261.3	1236.1	0.05	0.06
133	BBI	1295.4	1269.5	0.21	0.19
136	Mixed	1314.8	1288.5	0.07	0.06
141	BBI/NHCp	1342.7	1315.8	0.18	0.28
143	NHCp	1367.0	1339.7	0.18	0.26
144	BBI	1382.7	1355.0	0.05	0.03
155	NHCp/BBI	1456.0	1426.9	0.04	0.07
156	Mixed	1457.3	1428.1	0.15	0.11
165	BBI	1561.1	1529.9	0.46	0.46
166	NHCp/BBI	1583.5	1551.8	0.07	0.04
167	NHCp/BBI	1583.8	1552.1	0.15	0.08
170	NHCp	1605.9	1573.8	0.10	0.03
171	BBI	1611.7	1579.5	0.17	0.20
173	NHCp	1637.6	1604.9	1.00	0.57
175	BBI	1640.7	1607.9	0.05	0.05

*Table S 34: Calculated resonance Raman active vibrational modes of protonated **C1** at excitation wavelengths of 473 and 532 nm (unscaled and scaled by the factor of 0.98). Modes with relative Raman intensities ≥ 0.05 are given.*

			**C1^{prot}**		
Mode	Mode Type	ν / cm^{-1}	ν_{scaled} / cm^{-1}	Intensity (473 nm)	Intensity (532 nm)
95	NHCp	969.7	950.3	0.03	0.07
107	NHCp	1024.5	1004.0	0.05	0.04
108	NHCp	1027.3	1006.7	0.13	0.25
109	BBI/NHCp	1047.2	1026.2	0.11	0.15
121	NHCp/BBI	1128.0	1105.4	0.14	0.16
129	BBI	1230.6	1206.0	0.10	0.14
137	BBI	1299.2	1273.2	0.34	0.51
141	BBI	1334.6	1307.9	0.04	0.06
149	BBI/NHCp	1406.5	1378.3	0.11	0.16
153	BBI	1425.1	1396.6	0.36	0.48
164	BBI/NHCp	1502.2	1472.2	0.08	0.10
166	BBI	1509.3	1479.1	0.08	0.09
167	NHCp	1514.7	1484.4	0.09	0.11
169	NHCp	1583.9	1552.2	0.05	0.06
173	BBI	1611.2	1578.9	0.07	0.07
177	BBI	1649.3	1616.4	1.00	1.00

*Table S 35: Calculated resonance Raman active vibrational modes of deprotonated **C2** at excitation wavelengths of 473 and 532 nm (unscaled and scaled by the factor of 0.98). Modes with relative Raman intensities ≥ 0.05 are given.*

			**C2^{deprot}**		
Mode	Mode Type	ν / cm^{-1}	ν_{scaled} / cm^{-1}	Intensity (473 nm)	Intensity (532 nm)
94	NHCp	970.5	951.1	0.07	0.08
106	NHCp	1024.6	1004.1	0.22	0.23
107	BBI/NHCp	1049.3	1028.3	0.12	0.06
108	NHCp	1057.9	1036.8	0.05	0.06
120	BBI/NHCp	1126.4	1103.9	0.17	0.20
121	Mixed	1128.7	1106.2	0.61	0.71
137	Mixed	1315.5	1289.2	0.09	0.08
141	BBI/NHCp	1343.0	1316.2	0.06	0.03
143	NHCp	1369.5	1342.1	0.20	0.22
156	NHCp	1456.7	1427.6	0.10	0.09
164	NHCp	1512.2	1481.9	0.08	0.08
165	BBI	1561.1	1529.9	0.03	0.06
167	NHCp	1582.6	1550.9	0.05	0.04
169	NHCp	1603.6	1571.5	0.08	0.08
175	NHCp	1640.8	1608.0	0.04	0.06

*Table S 36: Calculated resonance Raman active vibrational modes of protonated **C2** at excitation wavelengths of 473 and 532 nm (unscaled and scaled by the factor of 0.98). Modes with relative Raman intensities ≥ 0.05 are given.*

**C2**^{prot}					
Mode	Mode Type	ν / cm^{-1}	ν_{scaled} / cm^{-1}	Intensity (473 nm)	Intensity (532 nm)
108	NHCp/BBI	1026.4	1005.8	0.08	0.04
109	BBI/NHCp	1049.5	1028.5	0.42	0.41
111	NHCp	1063.6	1042.3	0.21	0.07
113	NHCp	1071.0	1049.5	0.21	0.07
115	NHCp	1098.5	1076.5	0.08	0.03
121	NHCp/BBI	1126.8	1104.3	0.97	0.31
129	BBI	1232.3	1207.7	0.14	0.14
133	NHCp	1249.7	1224.7	0.11	0.03
135	NHCp	1270.4	1245.0	0.08	0.03
137	BBI	1301.3	1275.2	0.31	0.30
141	BBI	1333.7	1307.0	0.05	0.05
145	NHCp	1373.3	1345.8	0.17	0.06
149	BBI/NHCp	1407.6	1379.5	0.18	0.18
155	BBI	1428.1	1399.5	0.54	0.53
160	Mixed	1461.9	1432.7	0.06	0.02
163	NHCp	1472.3	1442.8	0.06	0.02
165	BBI	1509.2	1479.0	0.07	0.06
167	NHCp/BBI	1511.4	1481.2	0.09	0.04
170	NHCp	1583.9	1552.2	0.38	0.13
176	NHCp	1642.8	1610.0	0.17	0.07
177	BBI	1648.7	1615.8	1.00	1.00
179	BBI	1657.0	1623.9	0.22	0.22

11. Crystallography

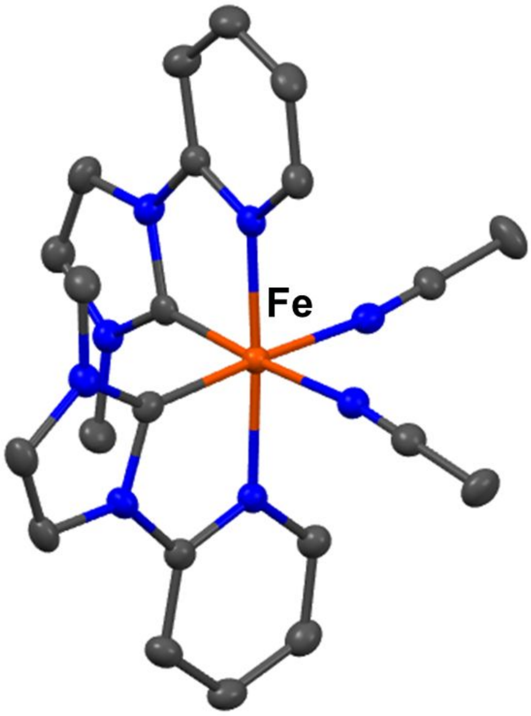

*Figure S 66: Solid-state structure of **P1** (Λ-isomer), single crystals obtained from acetonitrile/diethylether, ellipsoids drawn at 50% probability, hydrogen atoms and PF_6 counter ions omitted for clarity.*

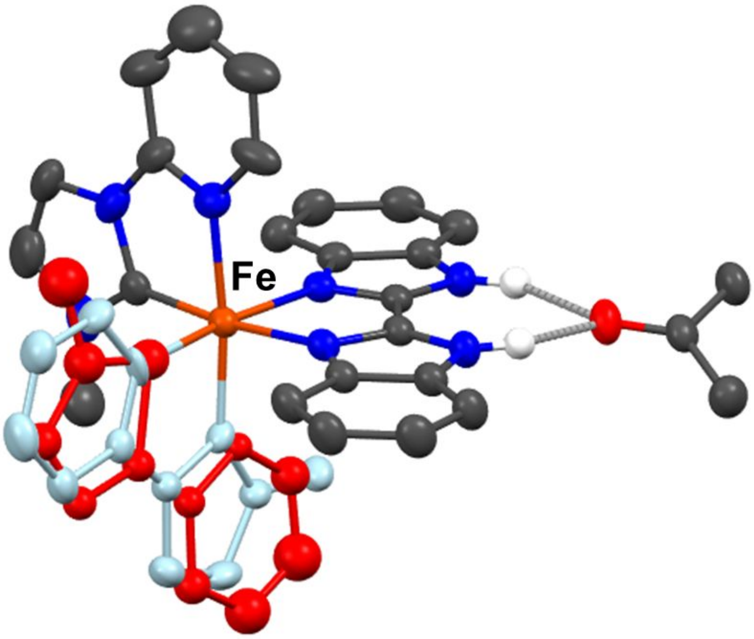

*Figure S 67: Solid-state structure of 7:3 **C2**:**C1** isomer mixture obtained from acetone/diethylether, ellipsoids drawn at 50% probability, **^{Me}NHCpy** ligand corresponding to isomer **C2** highlighted in red, **^{Me}NHCpy** ligand corresponding to isomer **C1** highlighted in light blue; hydrogen atoms, solvent molecules and PF_6 counter ions omitted for clarity except those engaging in hydrogen bonds.*

Table S 37: Crystal data and structure refinement for CCDC #2302719.

Identification code	**P1**
Empirical formula	$C_{22}H_{24}F_{12}FeN_8P_2$
Formula weight	746.26
Temperature/K	150.0
Crystal system	orthorhombic
Space group	$P2_12_12_1$
a/Å	10.6658(3)
b/Å	10.7577(3)
c/Å	24.2797(7)
α/°	90
β/°	90
γ/°	90
Volume/Å^3	2785.84(14)
Z	4
ρ_{calc}g/cm^3	1.7791
μ/mm^{-1}	0.768
F(000)	1507.3
Crystal size/mm^3	0.608 × 0.312 × 0.23
Radiation	Mo Kα (λ = 0.71073)
2Θ range for data collection/°	4.14 to 52.84
Index ranges	$-13 \leq h \leq 13$, $-13 \leq k \leq 13$, $-30 \leq l \leq 30$
Reflections collected	34077
Independent reflections	5725 [R_{int} = 0.0381, R_{sigma} = 0.0243]
Data/restraints/parameters	5725/0/409
Goodness-of-fit on F^2	1.035
Final R indexes [I>=2σ (I)]	R_1 = 0.0313, wR_2 = 0.0783
Final R indexes [all data]	R_1 = 0.0337, wR_2 = 0.0796
Largest diff. peak/hole / e Å^{-3}	0.54/-0.32
Flack parameter	0.341(18)

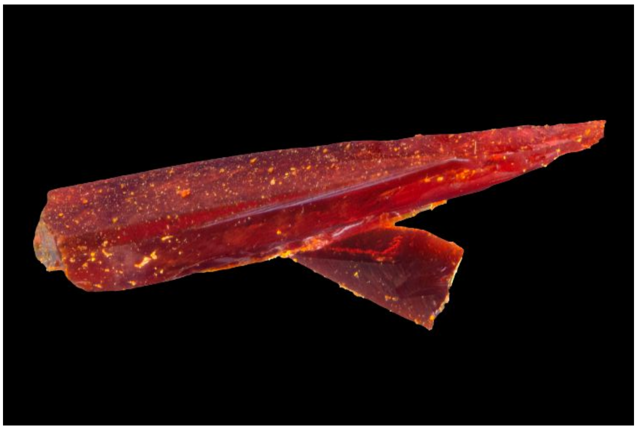

Figure S 68: Crystal of P1 grown from acetonitrile/diethylether; the image was created by Marius Müßler (M.Sc.) using focus-stacking with an approximate magnification of 8:1. For the final image 97 single frames were used.

Table S 38: Crystal data and structure refinement for 2302721.

Identification code	**C2**
Empirical formula	$C_{36}H_{40}F_{12}FeN_{10}O_2P_2$
Formula weight	990.57
Temperature/K	150.0
Crystal system	monoclinic
Space group	$P2_1/c$
a/Å	9.2108(2)
b/Å	30.7334(8)
c/Å	14.8642(4)
α/°	90
β/°	97.6880(10)
γ/°	90
Volume/Å^3	4169.92(18)
Z	4
ρ_{calc}g/cm^3	1.578
μ/mm^{-1}	0.540
F(000)	2024.0
Crystal size/mm^3	0.256 × 0.235 × 0.192
Radiation	MoKα (λ = 0.71073)
2Θ range for data collection/°	3.83 to 54.994
Index ranges	$-11 \leq h \leq 11, -39 \leq k \leq 39, -19 \leq l \leq 19$
Reflections collected	75171
Independent reflections	9560 [R_{int} = 0.0406, R_{sigma} = 0.0206]
Data/restraints/parameters	9560/0/575
Goodness-of-fit on F^2	1.004
Final R indexes [I>=2σ (I)]	R_1 = 0.0490, wR_2 = 0.1330
Final R indexes [all data]	R_1 = 0.0549, wR_2 = 0.1381
Largest diff. peak/hole / e Å^{-3}	1.15/-0.86

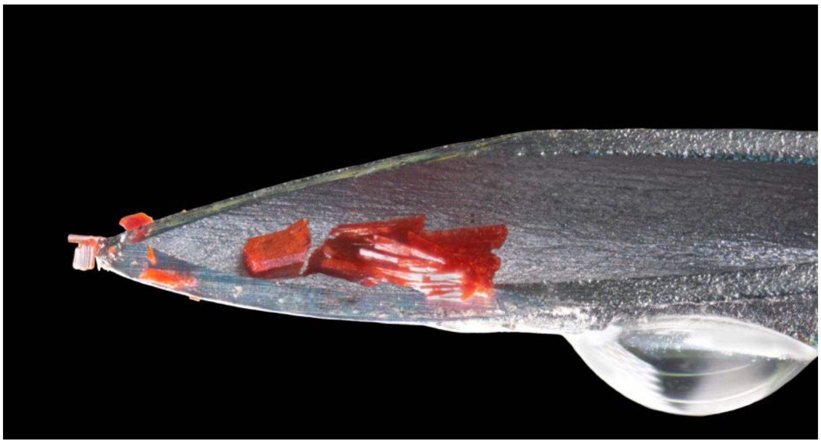

Figure S 69: Crystal of C2 grown from acetonitrile/methanol/diethylether, placed on the tip of a syringe needle; the image was created by Marius Müßler (M.Sc.) using focus-stacking with an approximate magnification of 8:1. For the final image 79 single frames were used.

Table S 39: Crystal data and structure refinement for 2302722.

Identification code	**C1**
Empirical formula	$C_{33}H_{32}F_{12}FeN_{10}OP_2$
Formula weight	930.47
Temperature/K	150.0
Crystal system	monoclinic
Space group	$P2_1/c$
a/Å	14.7690(6)
b/Å	18.6571(8)
c/Å	13.7745(6)
α/°	90
β/°	91.679(2)
γ/°	90
Volume/Å^3	3793.9(3)
Z	4
ρ_{calc}g/cm^3	1.629
μ/mm^{-1}	0.586
F(000)	1888.0
Crystal size/mm^3	0.347 × 0.252 × 0.198
Radiation	MoKα (λ = 0.71073)
2Θ range for data collection/°	3.676 to 55.238
Index ranges	$-19 \le h \le 18$, $-24 \le k \le 24$, $-17 \le l \le 17$
Reflections collected	82669
Independent reflections	8769 [R_{int} = 0.0721, R_{sigma} = 0.0392]
Data/restraints/parameters	8769/18/548
Goodness-of-fit on F^2	1.101
Final R indexes [I>=2σ (I)]	R_1 = 0.0781, wR_2 = 0.1882
Final R indexes [all data]	R_1 = 0.0830, wR_2 = 0.1910
Largest diff. peak/hole / e Å^{-3}	1.52/-0.68

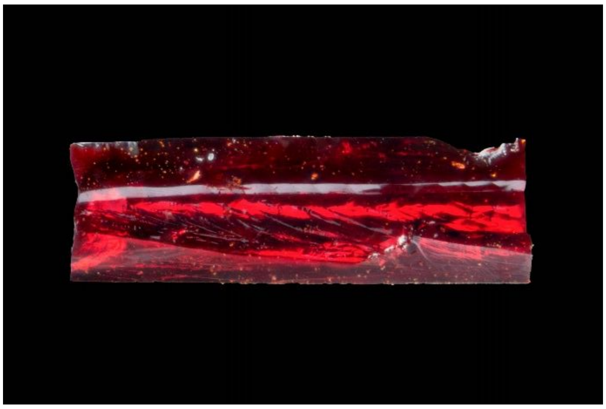

Figure S 70: Crystal of C2 grown from acetonitrile/methanol/diethylether, placed on the tip of a syringe needle; the image was created by Marius Müßler (M.Sc.) using focus-stacking with an approximate magnification of 8:1. For the final image 113 single frames were used.

Table S 40: Crystal data and structure refinement for 2302720.

Identification code	**C2/C1 (7:3)**
Empirical formula	$C_{35}H_{34}F_{12}FeN_{10}OP_2$
Formula weight	956.51
Temperature/K	150.0
Crystal system	monoclinic
Space group	$P2_1/c$
a/Å	15.0072(8)
b/Å	18.5573(9)
c/Å	14.0720(7)
α/°	90
β/°	92.283(2)
γ/°	90
Volume/Å^3	3915.8(3)
Z	4
ρ_{calc}g/cm^3	1.622
μ/mm^{-1}	0.570
F(000)	1944.0
Crystal size/mm^3	0.321 × 0.182 × 0.164
Radiation	MoKα (λ = 0.71073)
2Θ range for data collection/°	3.492 to 55.034
Index ranges	$-19 \leq h \leq 19, -24 \leq k \leq 24, -18 \leq l \leq 18$
Reflections collected	78038
Independent reflections	8983 [R_{int} = 0.0564, R_{sigma} = 0.0295]
Data/restraints/parameters	8983/0/603
Goodness-of-fit on F^2	1.083
Final R indexes [I>=2σ (I)]	R_1 = 0.0492, wR_2 = 0.1185
Final R indexes [all data]	R_1 = 0.0638, wR_2 = 0.1256
Largest diff. peak/hole / e Å^{-3}	1.08/-0.42

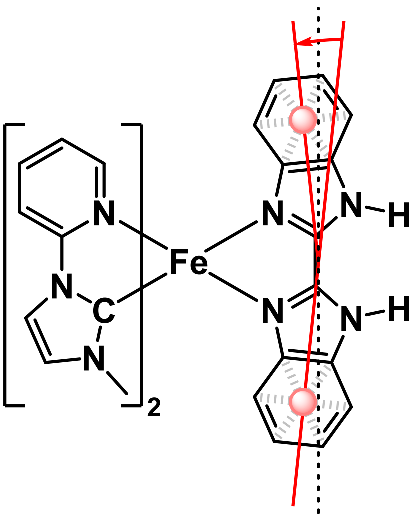

*Figure S 71: Schematic depiction of how bending angles of the 2,2'-bibenzimidazole ligand were determined for **C1**, **C2**, as well as complexes from literature, CCDC entries #182296 and #144399; red circles indicate centroids of the benzyl rings, angles were measured using Diamond 4.6.8, Crystal Impact GbR*

Table S 41: Crystal data and structure refinement for CCDC #2355848

Identification code	$[(18\text{-crown-6})K]_2S_2O_8$
Empirical formula	$C_{24}H_{48}K_2O_{20}S_2$
Formula weight	798.94
Temperature/K	150.0
Crystal system	monoclinic
Space group	$P2_1/n$
a/Å	11.735(8)
b/Å	8.366(6)
c/Å	18.865(11)
α/°	90
β/°	99.94(2)
γ/°	90
Volume/Å^3	1824(2)
Z	2
ρ_{calc}g/cm^3	1.454
μ/mm^{-1}	0.452
F(000)	844.0
Crystal size/mm^3	0.1 × 0.1 × 0.1
Radiation	MoKα (λ = 0.71073)
2Θ range for data collection/°	4.384 to 54.944
Index ranges	$-15 \le h \le 15$, $-10 \le k \le 10$, $-24 \le l \le 24$
Reflections collected	39786
Independent reflections	4165 [R_{int} = 0.0400, R_{sigma} = 0.0196]
Data/restraints/parameters	4165/0/217
Goodness-of-fit on F^2	1.035
Final R indexes [I>=2σ (I)]	R_1 = 0.0252, wR_2 = 0.0618
Final R indexes [all data]	R_1 = 0.0295, wR_2 = 0.0639
Largest diff. peak/hole / e Å^{-3}	0.27/-0.36

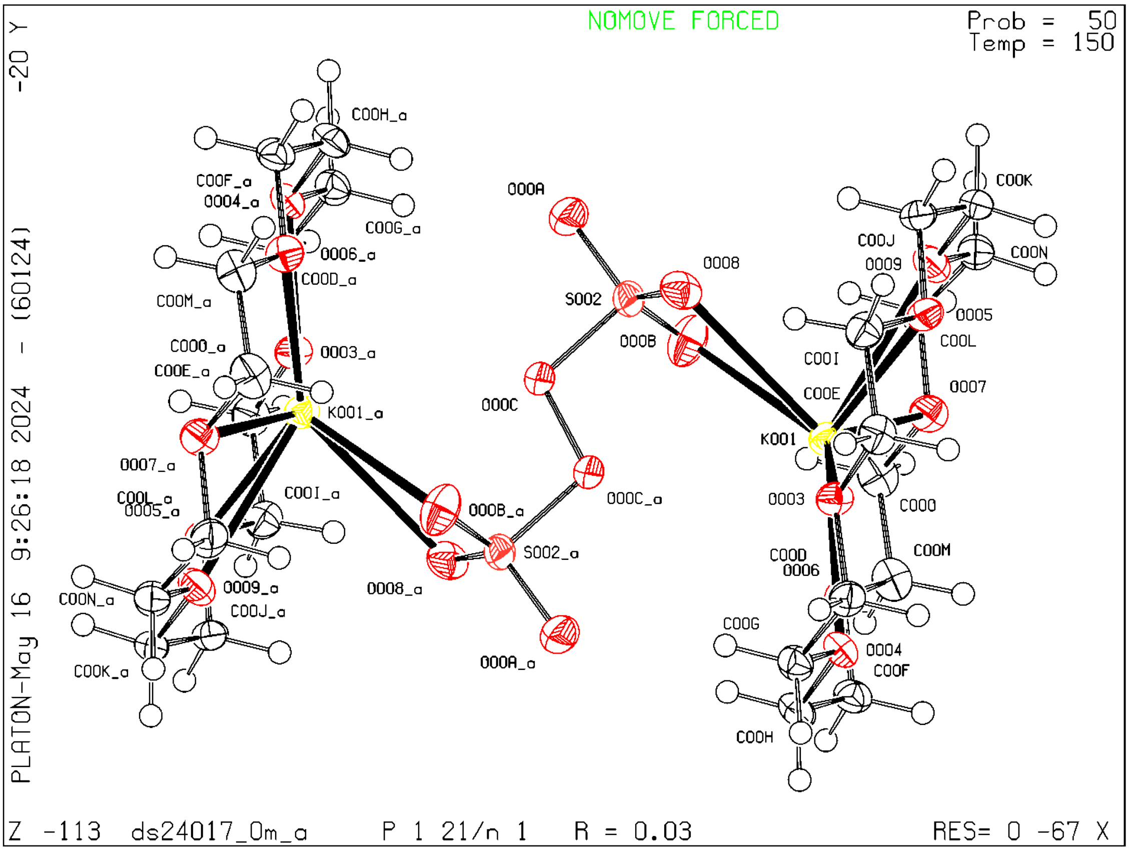

Figure S 72: ORTEP depiction of $[(18\text{-crown-}6)K]_2S_2O_8$

Table S 42: Crystal data and structure refinement for CCDC #2355849.

Identification code	C2-TFA
Empirical formula	$C_{36}H_{28}F_6FeN_{10}O_4$
Formula weight	834.53
Temperature/K	150.0
Crystal system	triclinic
Space group	P-1
a/Å	9.3825(3)
b/Å	11.2381(3)
c/Å	17.2025(5)
α/°	96.8670(10)
β/°	95.7170(10)
γ/°	97.6720(10)
Volume/Å^3	1772.37(9)
Z	2
ρ_{calc}g/cm^3	1.564
μ/mm^{-1}	0.514
F(000)	852.0
Crystal size/mm^3	0.1 × 0.1 × 0.1
Radiation	MoKα (λ = 0.71073)
2Θ range for data collection/°	4.124 to 54.998
Index ranges	$-12 \le h \le 12$, $-13 \le k \le 14$, $-22 \le l \le 22$
Reflections collected	40146
Independent reflections	8145 [R_{int} = 0.0497, R_{sigma} = 0.0336]
Data/restraints/parameters	8145/0/543
Goodness-of-fit on F^2	1.044
Final R indexes [I>=2σ (I)]	R_1 = 0.0380, wR_2 = 0.0855
Final R indexes [all data]	R_1 = 0.0457, wR_2 = 0.0894
Largest diff. peak/hole / e Å^{-3}	0.43/-0.38

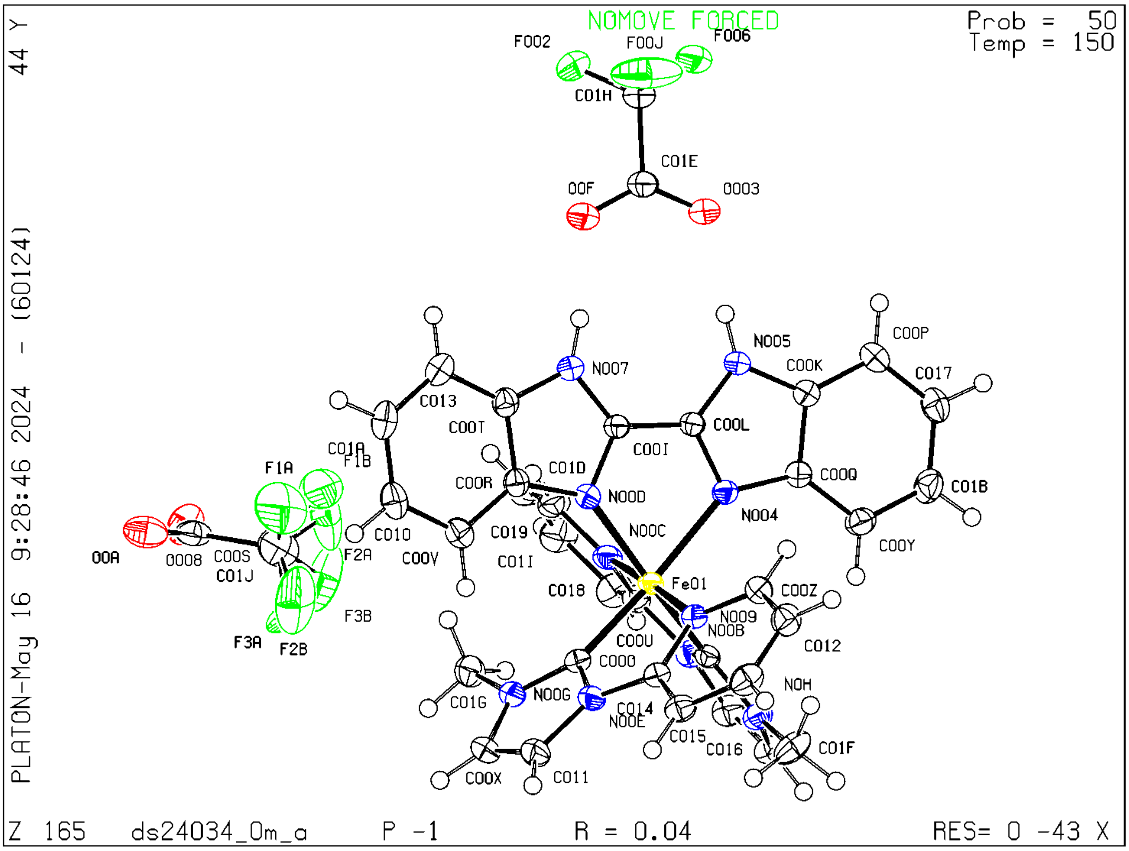

Figure S 73: ORTEP depiction of C2^{prot} trifluoroacetic (C2^{prot}-TFA)acid salt.

12. Reactivity Studies

12.1 Oxidation of C1 and C2 with $[(18\text{-crown-6})K]_2S_2O_8$

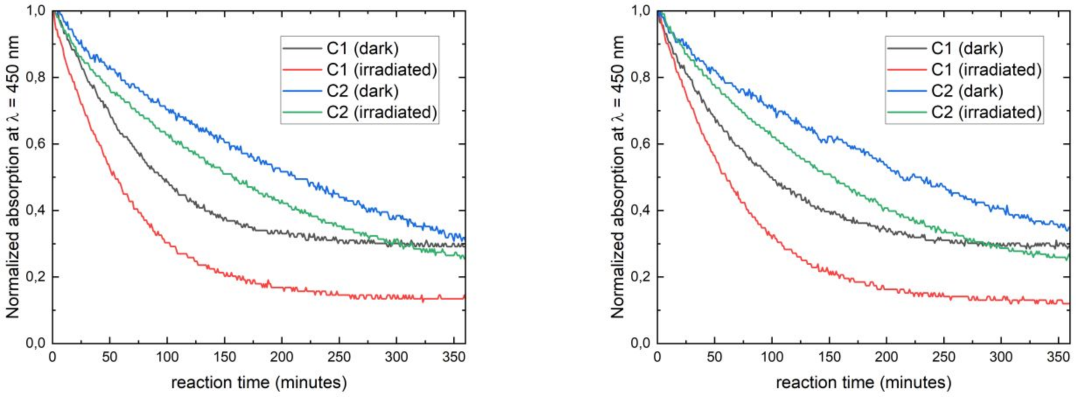

*Figure S 74: Oxidation of **C1** and **C2** (10-5 M) with $[(18\text{-crown-6})K]_2S_2O_8$ (10-4 M) in dry acetonitrile as characterized by a bleach of the MLCT absorption at 450 nm in solutions of the respective complexes; depicted are the results from two separate measurements; normalized absorption of identical solutions at λ = 450 nm measured over a period of 360 minutes in intervals of 1 minute; purple: C2 in the dark; light blue: C2 under irradiation with an LED stick at λ = 470 nm; maroon: **C1** in the dark; bright red: C2 under irradiation with an LED stick at λ = 470 nm; the respective UV-vis spectra are depicted in Figures S 75-78.*

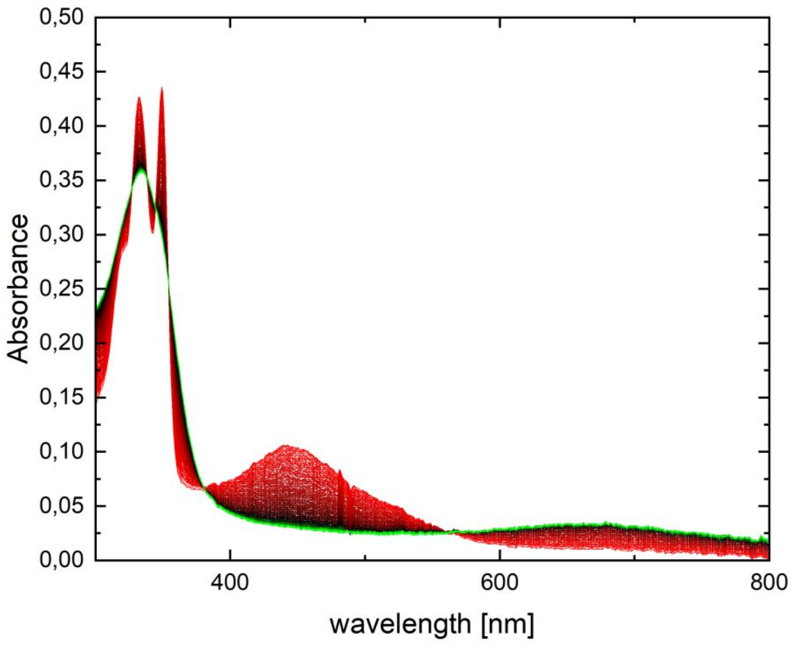

*Figure S 75 UV-vis spectra showing the oxidation of **C1** (10^{-5} M) with $[(18\text{-crown-6})K]_2S_2O_8$ (10^{-4} M) in acetonitrile solution in the absence of light over a time period of 6 hours; one spectrum recorded every minute, color code: red (0 minutes) over black to green (360 minutes)*

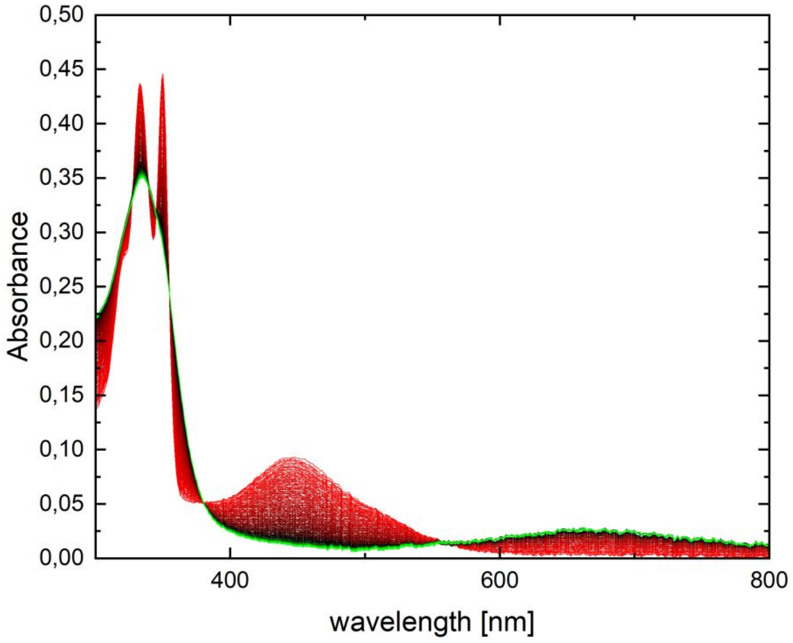

*Figure S 76: UV-vis spectra showing the oxidation of **C1** (10^{-5} M) with [(18-crown-6)K]$_2$S$_2$O$_8$ (10^{-4} M) in acetonitrile solution under irradiation with an LED at λ = 470 nm over a time period of 6 hours; one spectrum recorded every minute, color code: red (0 minutes) over black to green (360 minutes)*

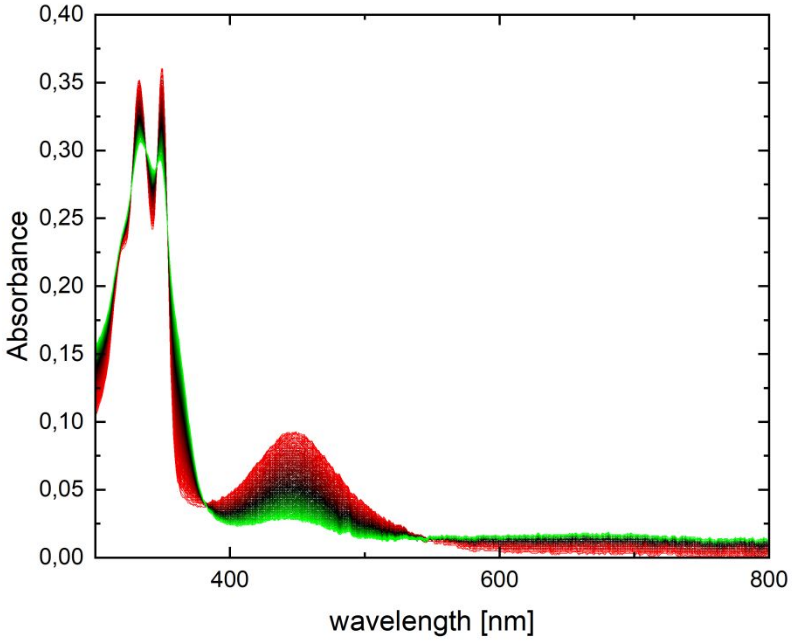

*Figure S 77 UV-vis spectra showing the oxidation of **C2** (10^{-5} M) with [(18-crown-6)K]$_2$S$_2$O$_8$ (10^{-4} M) in acetonitrile solution in the absence of light over a time period of 6 hours; one spectrum recorded every minute, color code: red (0 minutes) over black to green (360 minutes)*

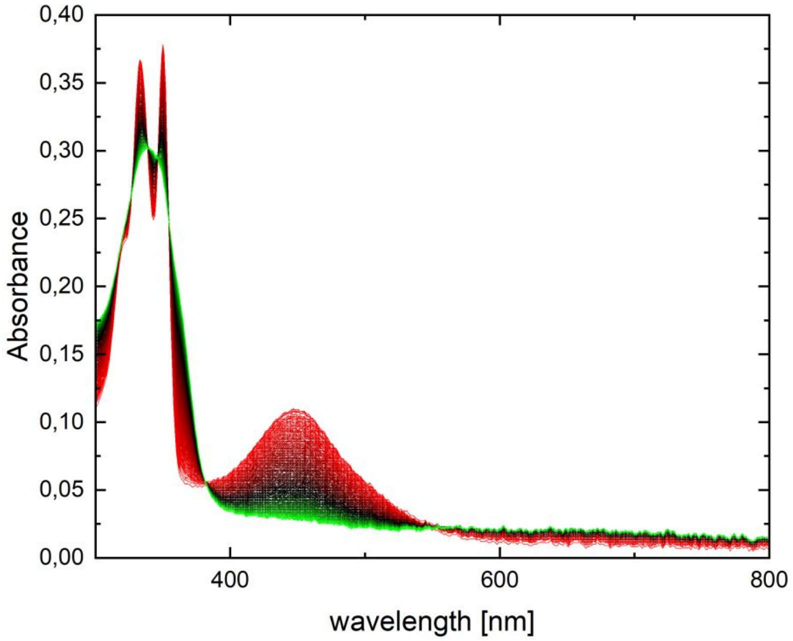

Figure S 78: UV-vis spectra showing the oxidation of ***C2*** *(10^{-5} M) with [(18-crown-6)K]$_2$S$_2$O$_8$ (10^{-4} M) in acetonitrile solution under irradiation with an LED at λ = 470 nm over a time period of 6 hours; one spectrum recorded every minute, color code: red (0 minutes) over black to green (360 minutes)*

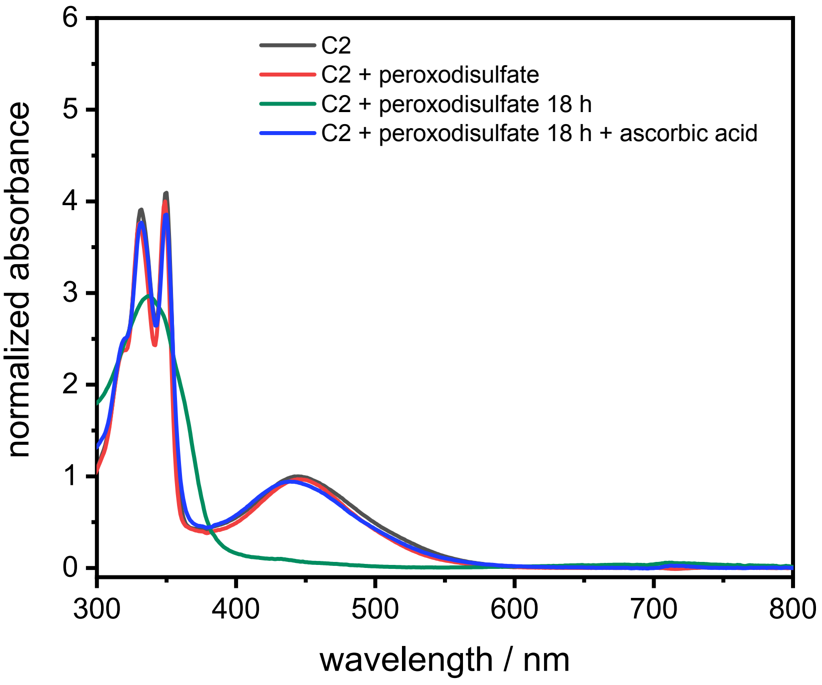

*Figure S 79: UV-vis spectra of **C2** (10^{-5} M, black) and after addition of 10 eq. $[(18\text{-crown-}6)K]_2S_2O_8$ (red); spectra of the oxidized complex after 18 hours at room temperature (green) and after addition of ascorbic acid (excess solid) showing the spectrum of fully recovered (blue) of **C2**. Spectra are normalized to the MLCT maximum of **C2***

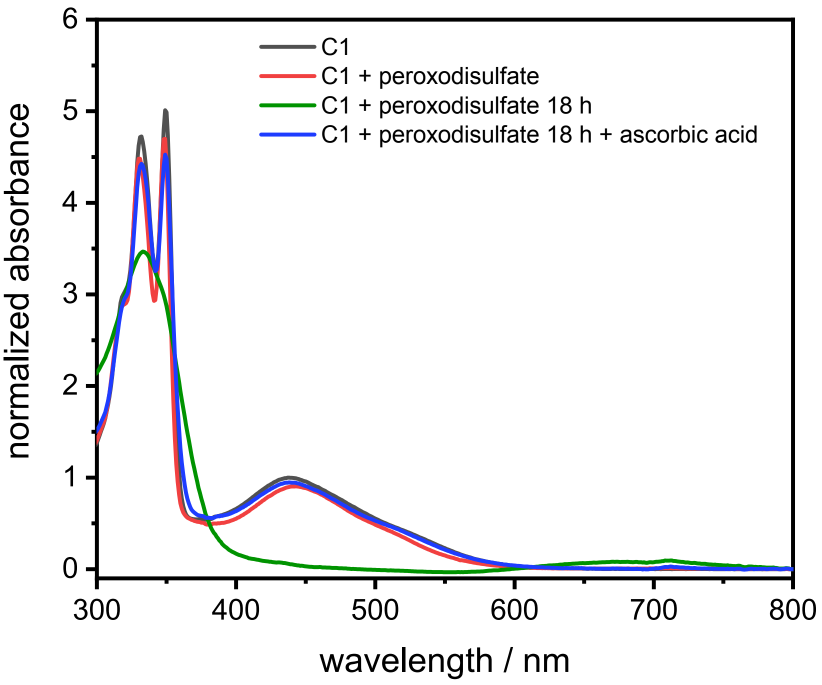

*Figure S 80: UV-vis spectra of **C1** (10^{-5} M, black) and after addition of 10 eq. [(18-crown-6)K]$_2S_2O_8$ (red); spectra of the oxidized complex after 18 hours at room temperature (green) and after addition of ascorbic acid (excess solid) showing the spectrum of fully recovered C1 (blue). Spectra are normalized to the MLCT maximum of **C1***

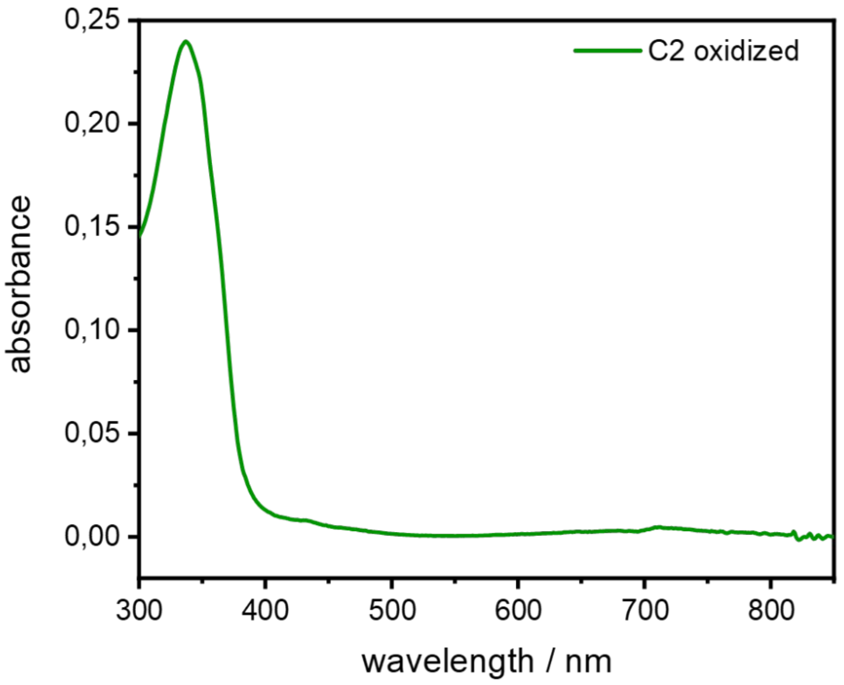

*Figure S 81: UV-vis spectrum of **C2** (10^{-5} M) after the reaction with peroxodisulfate.*

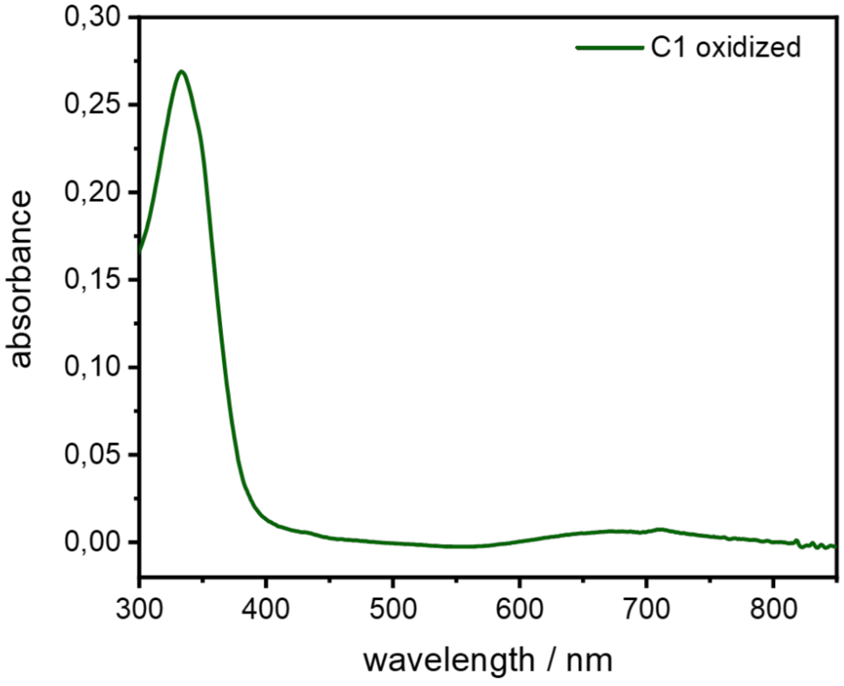

*Figure S 82: UV-vis spectrum of **C1**(10^{-5} M) after the reaction with peroxodisulfate.*

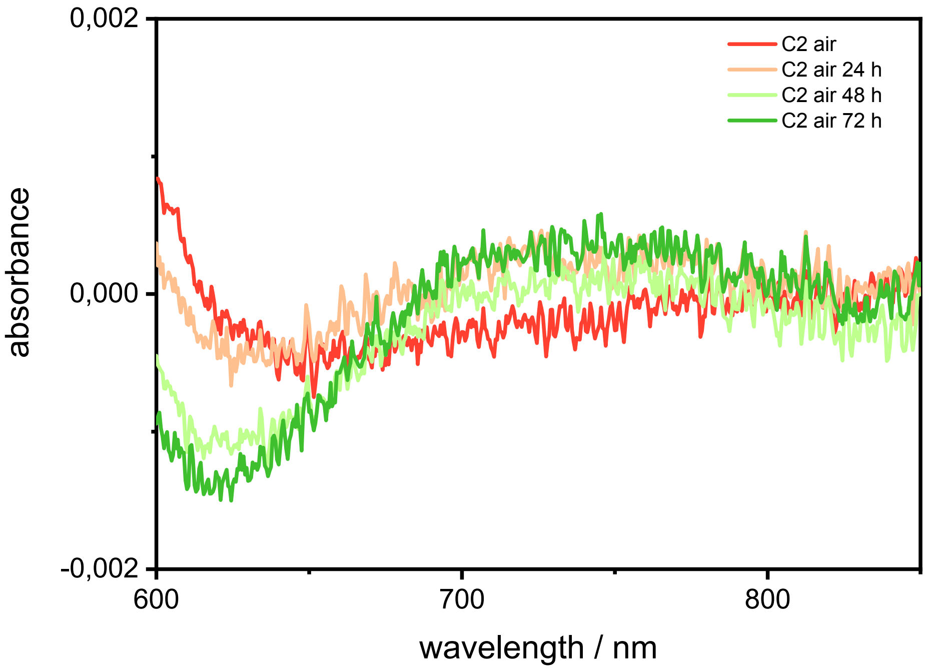

Figure S 83: Absorption spectrum of ***C2*** *(10^{-5} M) under irradiation in the presence of air showing the appearance of a broad absorption characteristic for oxidation of the chromophore.*

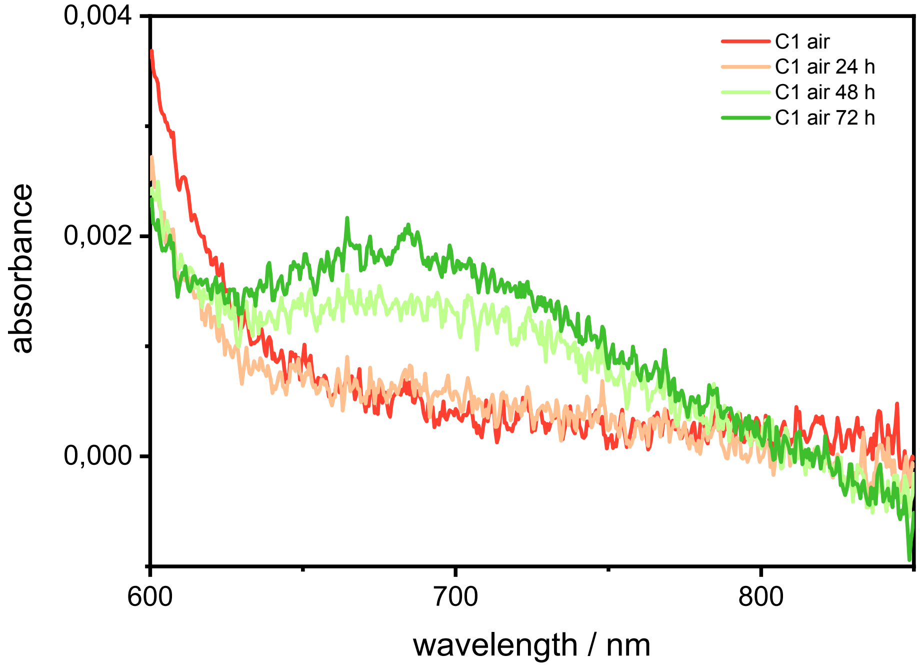

Figure S 84: Absorption spectrum of ***C1****(10^{-5} M) under irradiation in the presence of air showing the appearance of a broad absorption characteristic for oxidation of the chromophore.*

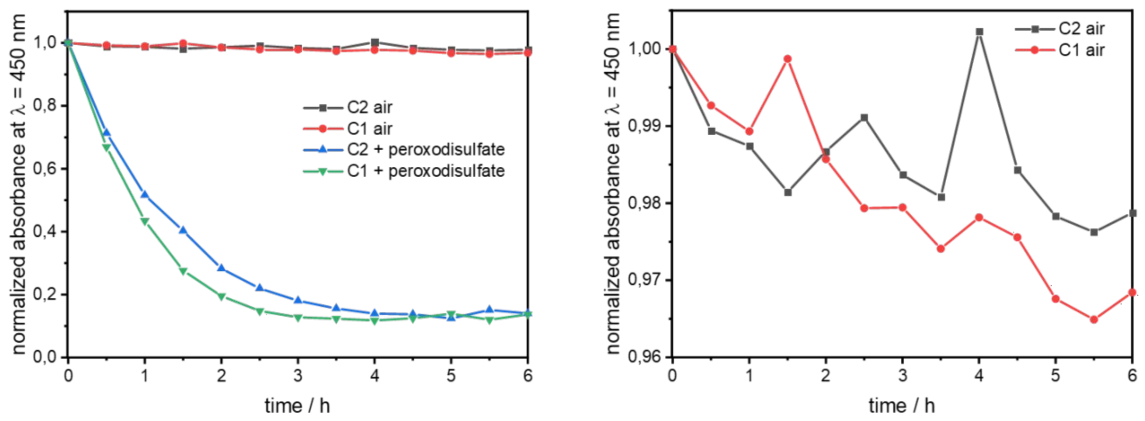

*Figure S 85: Left: Oxidation of **C1** and **C2** (10^{-5} M) under irradiation with an LED stick at λ = 470 nm in aerated acetonitrile in the presence (blue, green) and absence (red, black) of [(18-crown-6)K]$_2S_2O_8$ (10^{-4} M) as characterized by a bleach of the MLCT absorption at λ = 450 nm over a period of 6 hours; right: same data only in the absence of [(18-crown-6)K]$_2S_2O_8$.*

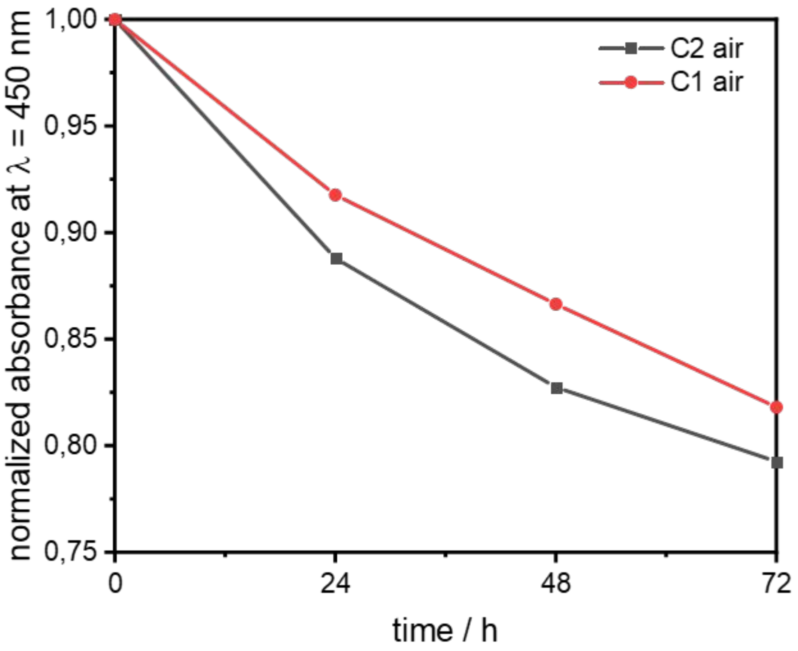

*Figure S 86: Oxidation of **C1** and **C2** (10^{-5} M) under irradiation with an LED stick at λ = 470 nm in aerated acetonitrile as characterized by a bleach of the MLCT absorption at λ = 450 nm over a period of 72 hours.*

12.2 Photostability

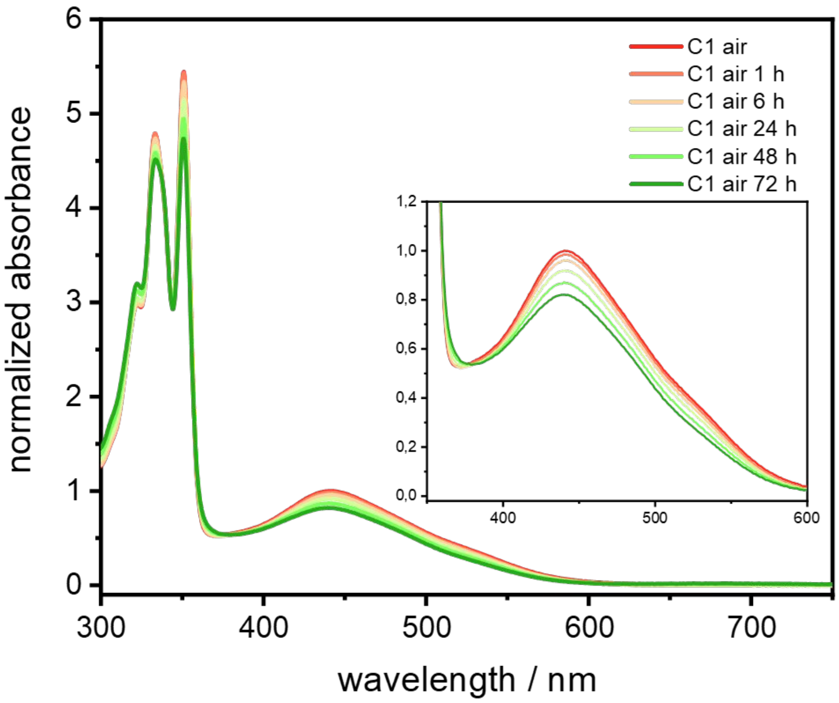

Figure S 87: Photostability test (c = 10^{-5} M): UV-vis spectra of C1 (aerobic) under irradiation; spectral changes are associated with oxidation of C1. Spectra are normalized to the MLCT maximum of ***C1***

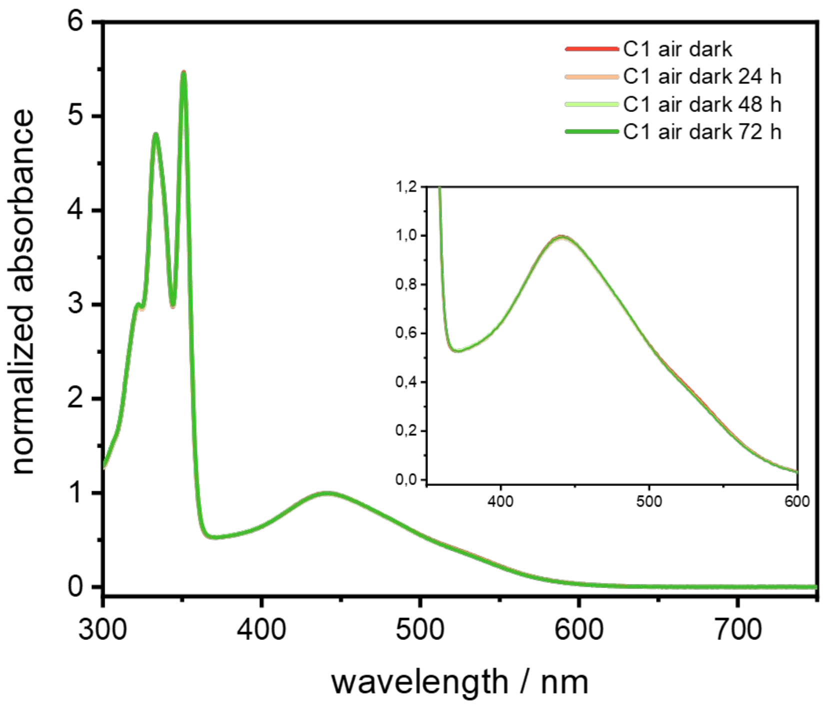

Figure S 88: Photostability test (c = 10^{-5} M): UV-vis spectra of C1 (aerobic) in the dark; no spectral changes can be observed. Spectra are normalized to the MLCT maximum of ***C1***

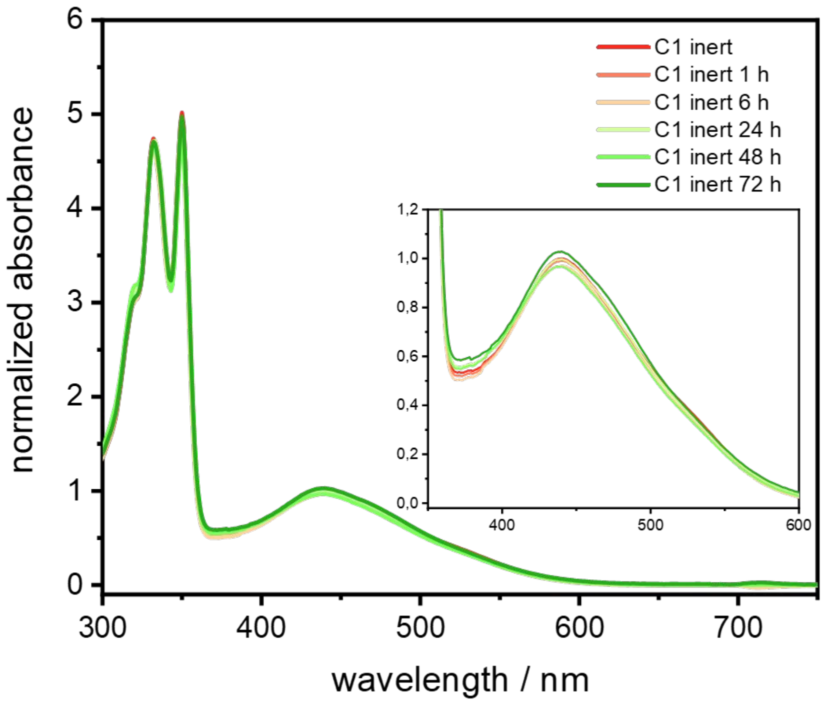

Figure S 89: Photostability test (c = 10^{-5} M): UV-vis spectra of C1 (inert) under irradiation; the small changes in the spectra are associated with drifts of the device, since all inert measurements are showing minor random changes in between measurements over time. Spectra are normalized to the MLCT maximum of ***C1***

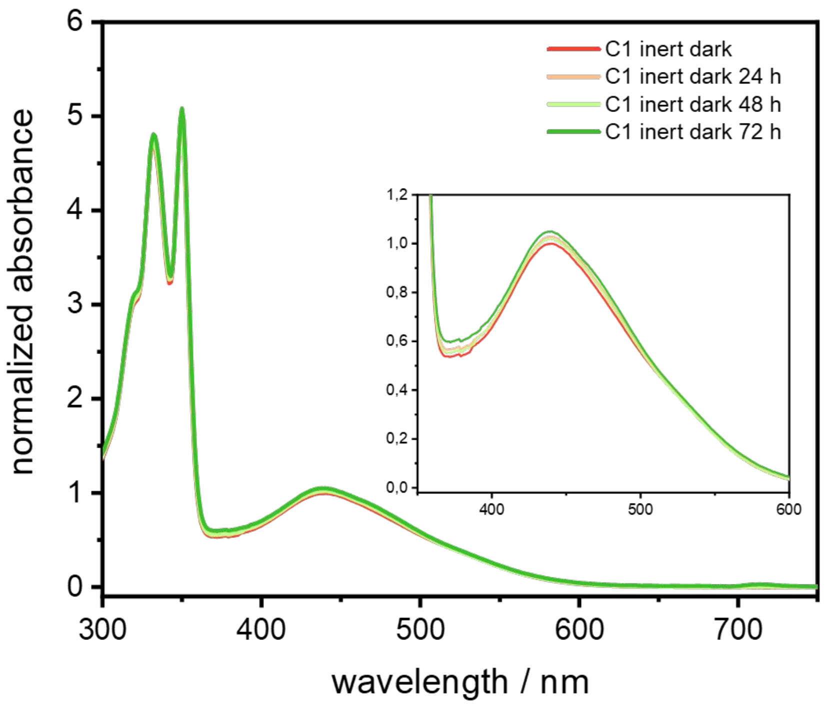

Figure S 90: Photostability test (c = 10^{-5} M): UV-vis spectra of C1 (inert) in the dark; the small changes in the spectra are associated with drifts of the device, since all inert measurements are showing minor random changes in between measurements over time. Spectra are normalized to the MLCT maximum of ***C1***

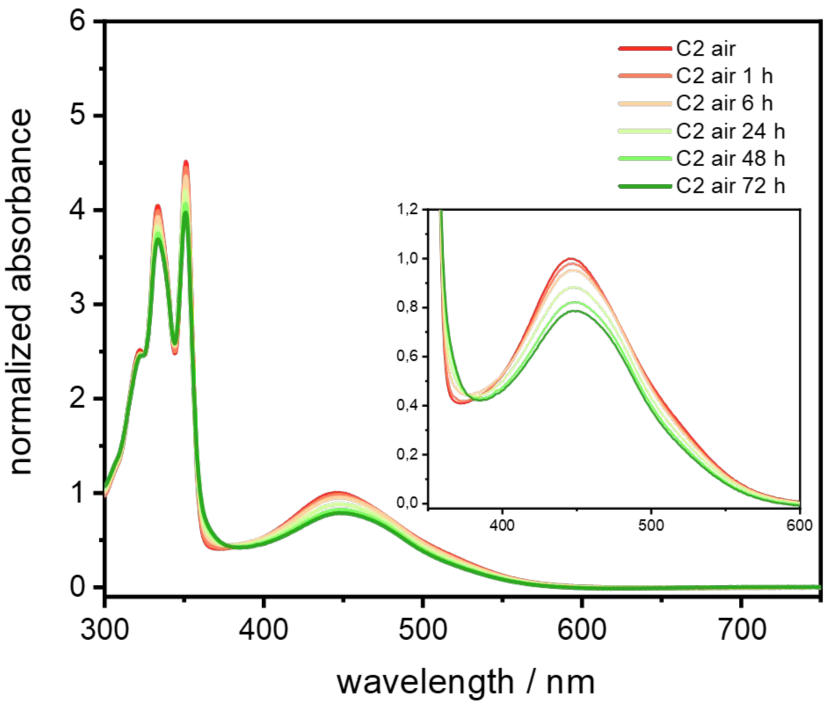

Figure S 91: Photostability test (c = 10^{-5} M): UV-vis spectra of C2 (aerobic) under irradiation; spectral changes are associated with oxidation of C2. Spectra are normalized to the MLCT maximum of ***C2***

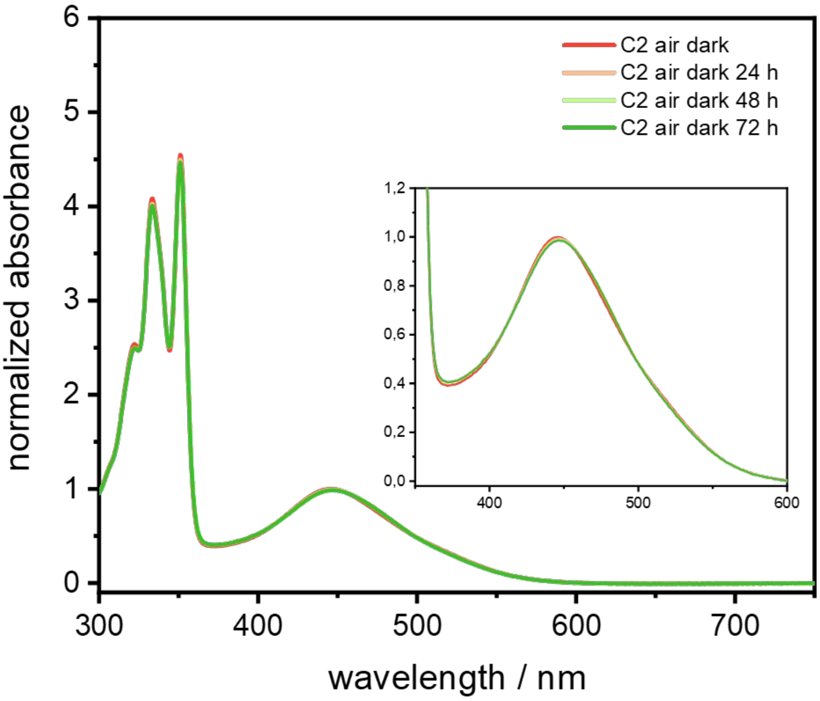

*Figure S 92: Photostability test (c = 10^{-5} M): UV-vis spectra of C2 (aerobic) in the dark; no spectral changes can be observed. Spectra are normalized to the MLCT maximum of **C2***

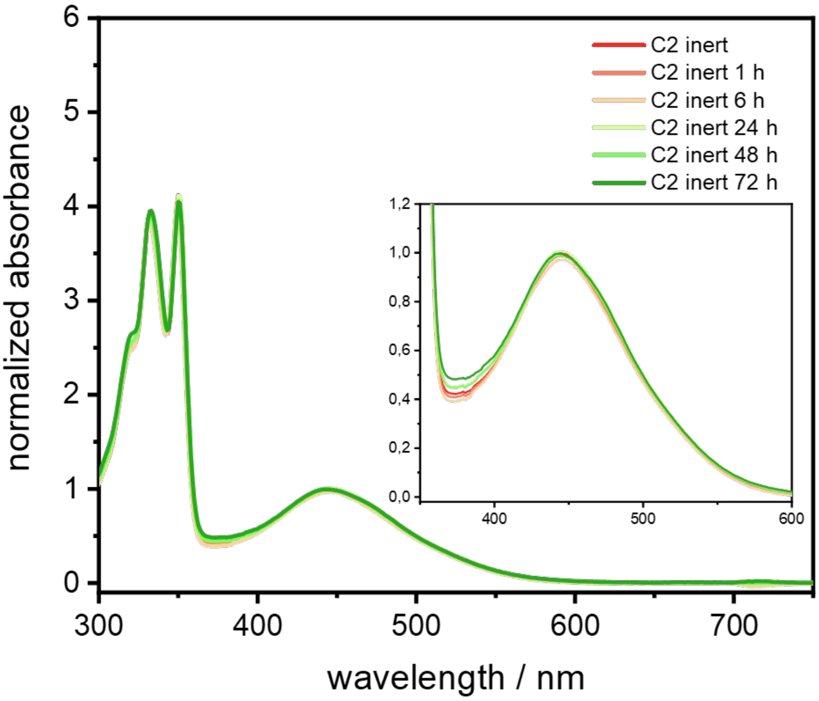

*Figure S 93: Photostability test (c = 10^{-5} M): UV-vis spectra of C2 (inert) under irradiation; the small changes in the spectra are associated with drifts of the device, since all inert measurements are showing minor random changes in between measurements over time. Spectra are normalized to the MLCT maximum of **C2***

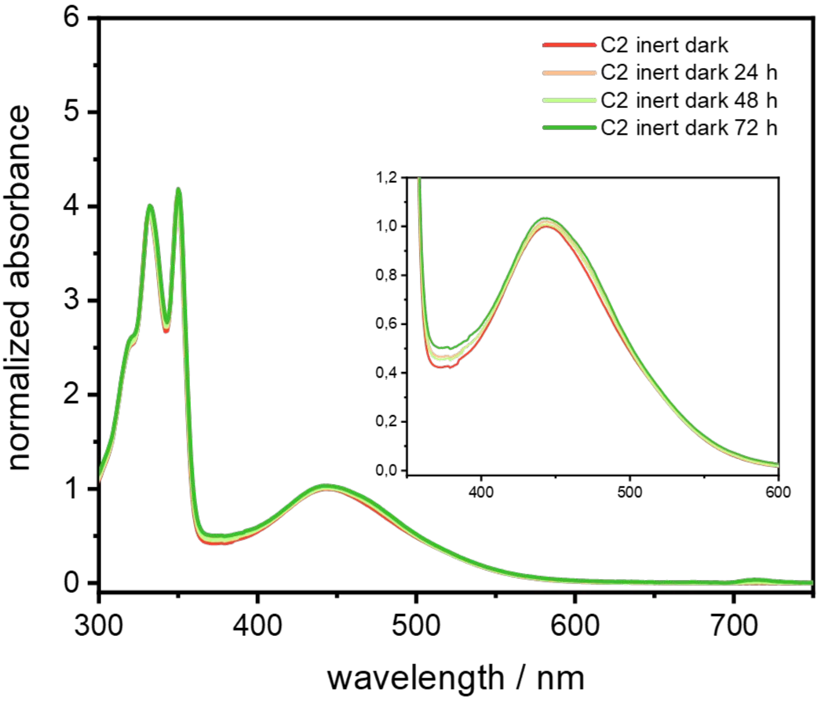

Figure S 94: Photostability test (c = 10^{-5} M): UV-vis spectra of C2 (inert) in the dark; the small changes in the spectra are associated with drifts of the device, since all inert measurements are showing minor random changes in between measurements over time. Spectra are normalized to the MLCT maximum of ***C2***

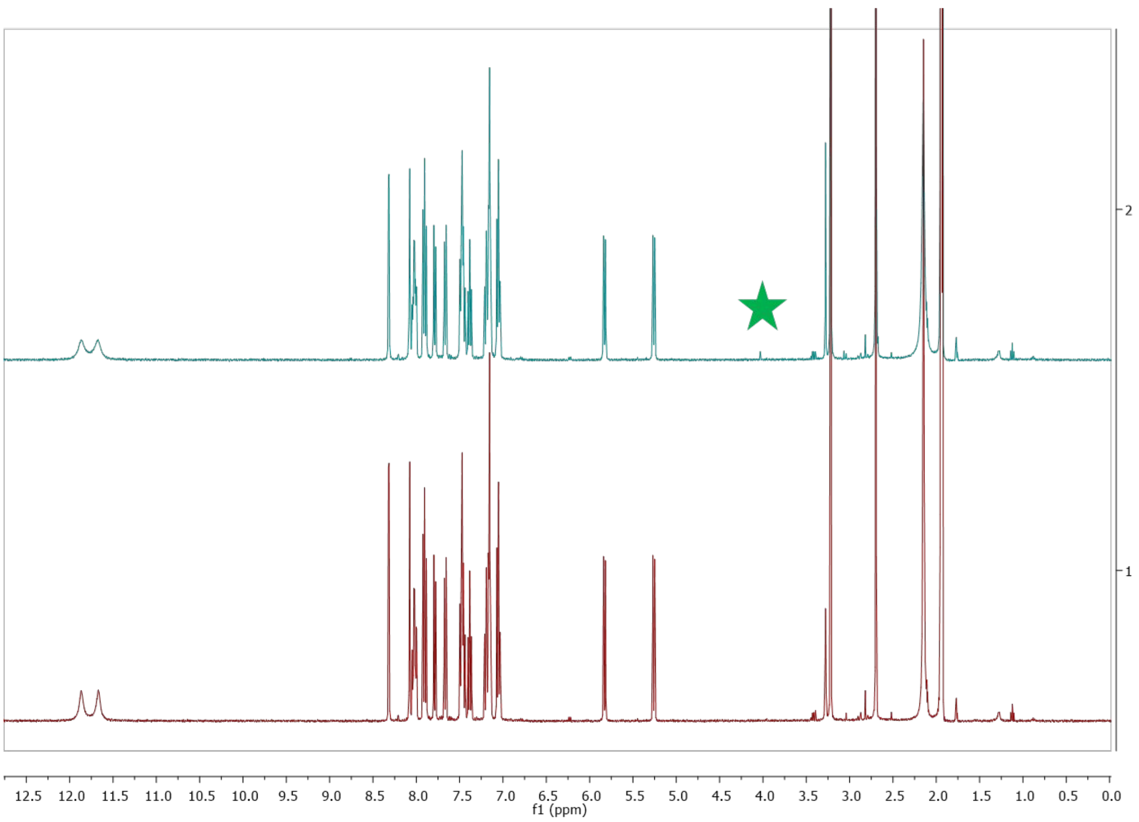

*Figure S 95: ^{1}H-NMR spectrum of **C1** in ACN-d_3 under irradiation (inert) (red :0 h; blue 72 h). The signal at 4.03 ppm (marked by green star) indicates the formation of P1*

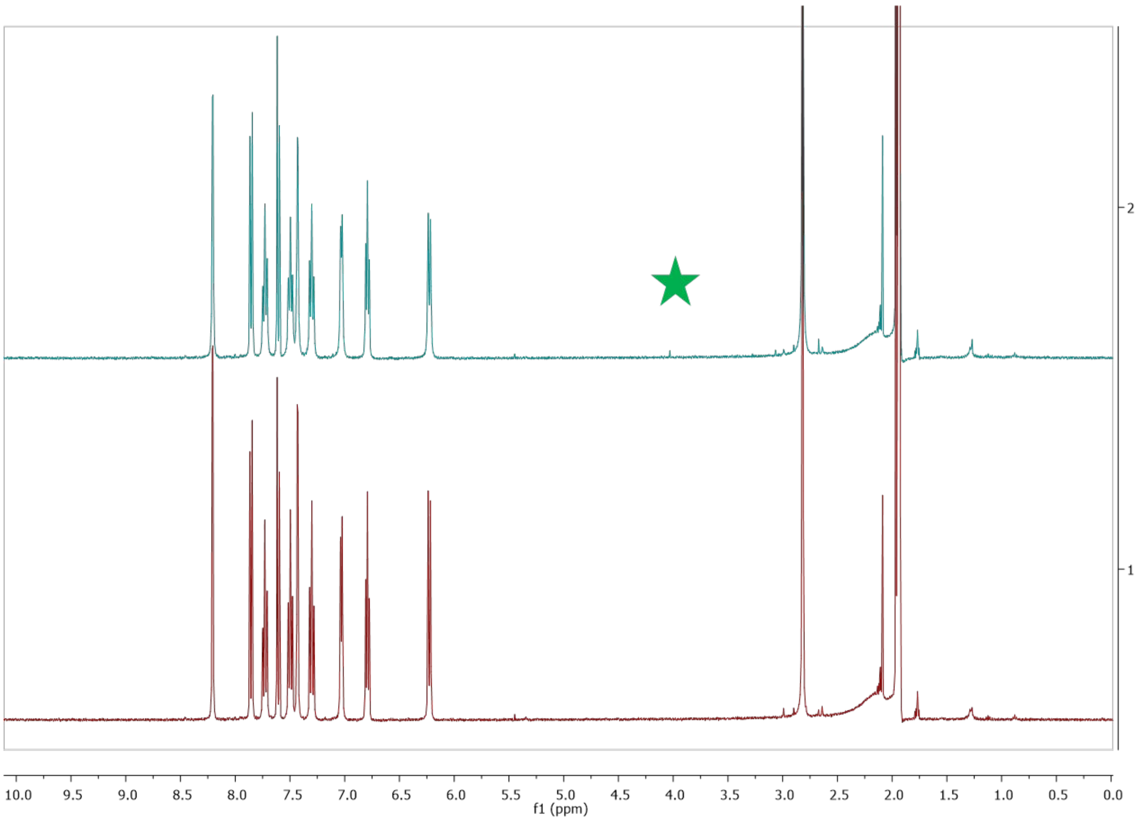

*Figure S 96: ^{1}H-NMR spectrum of **C2** in ACN-d_3 under irradiation (inert) (red :0 h; blue 72 h). The signal at 4.03 ppm (marked by green star) indicates the formation of P1.*

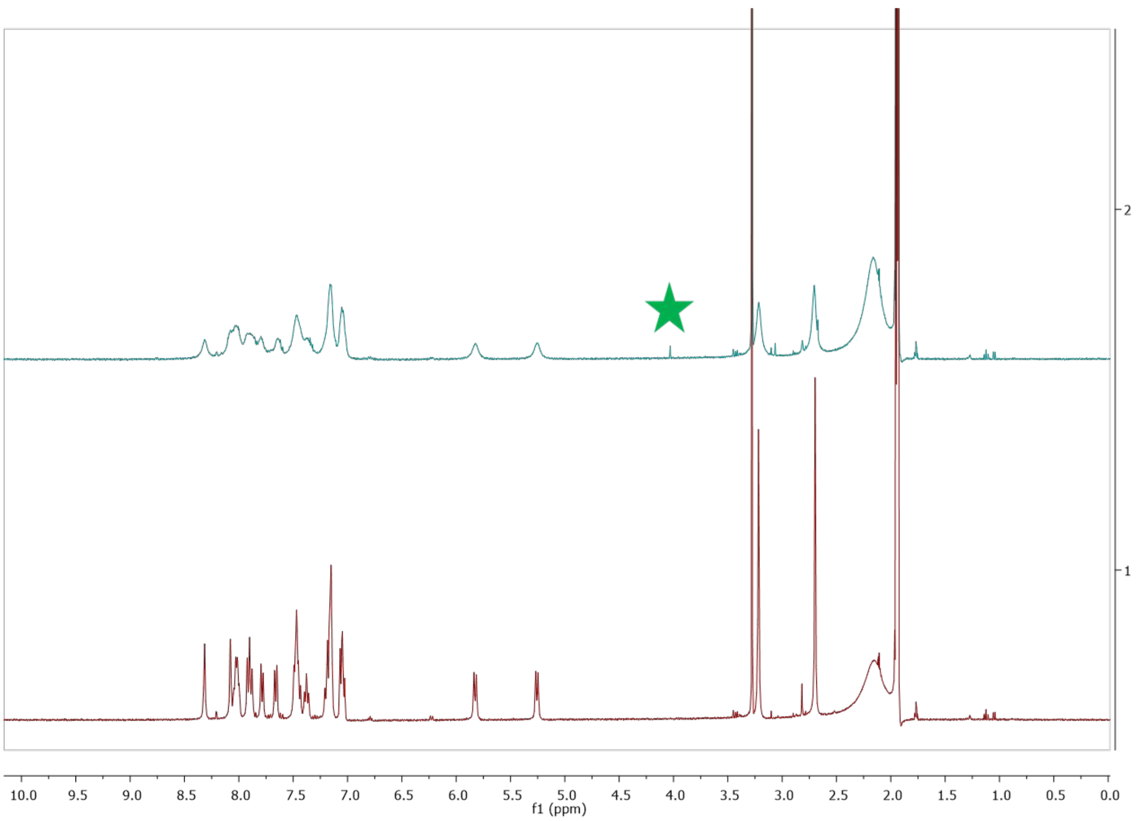

*Figure S 97: ^{1}H-NMR spectrum of **C1** in ACN-d_3 under irradiation (air) (red :0 h; blue 72 h). The signal at 4.03 ppm (marked by green star) indicates the formation of P1*

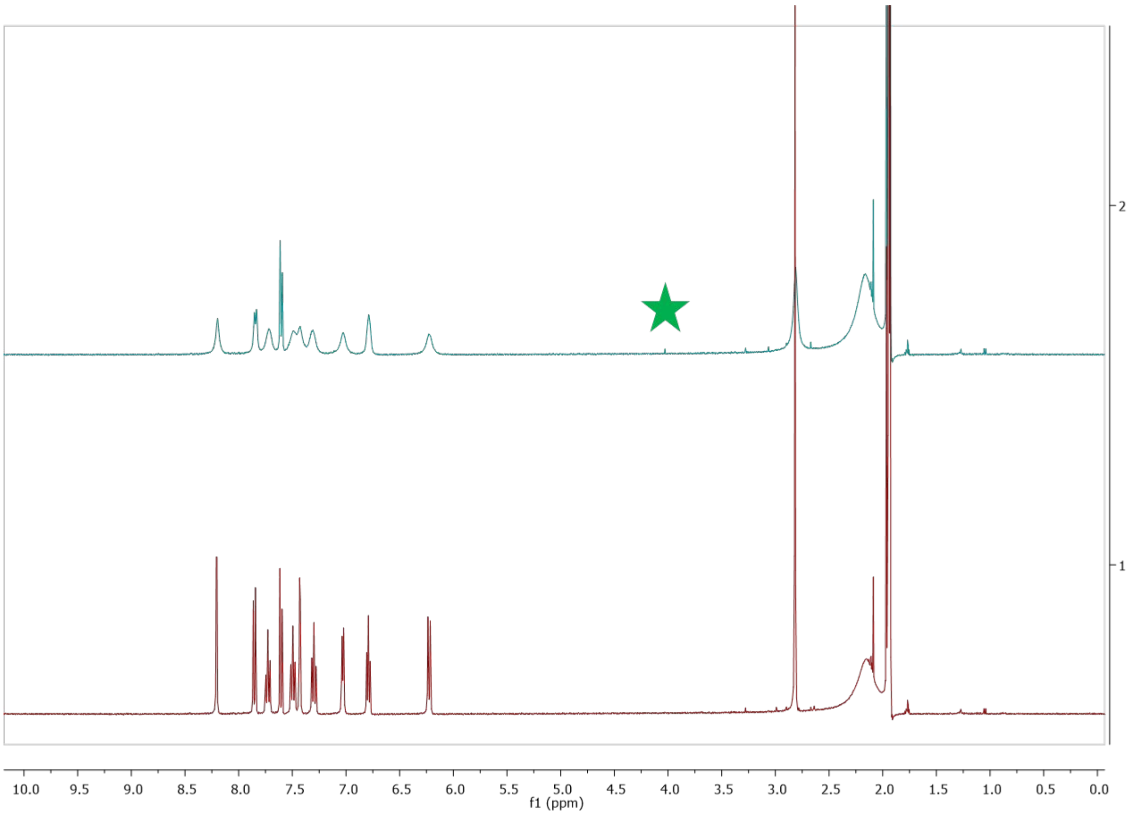

*Figure S 98: ^{1}H-NMR spectrum of **C2** in ACN-d_3 under irradiation (air) (red :0 h; blue 72 h). The signal at 4.03 ppm (marked by green star) the formation of P1*

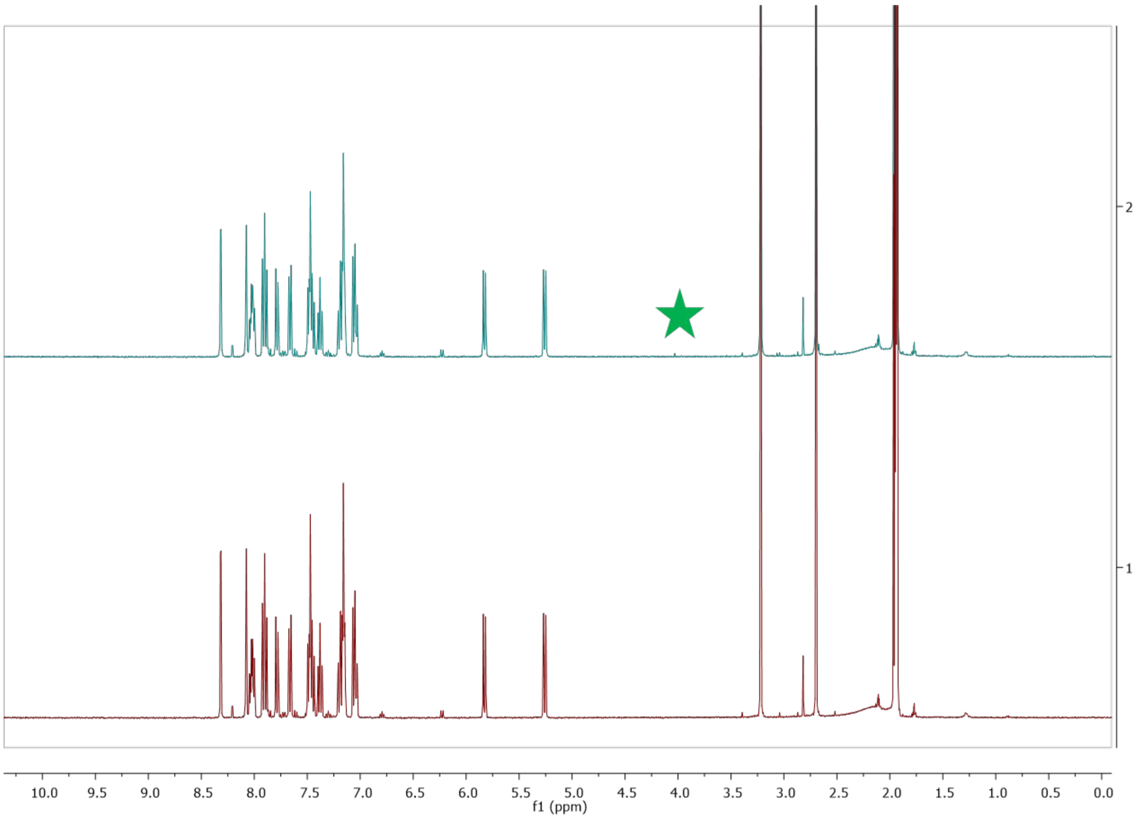

*Figure S 99: ^{1}H-NMR spectrum of **C1** in ACN-d_3 in the dark (inert) (red :0 h; blue 72 h). The signal at 4.03 ppm (marked by green star) indicates the formation of P1*

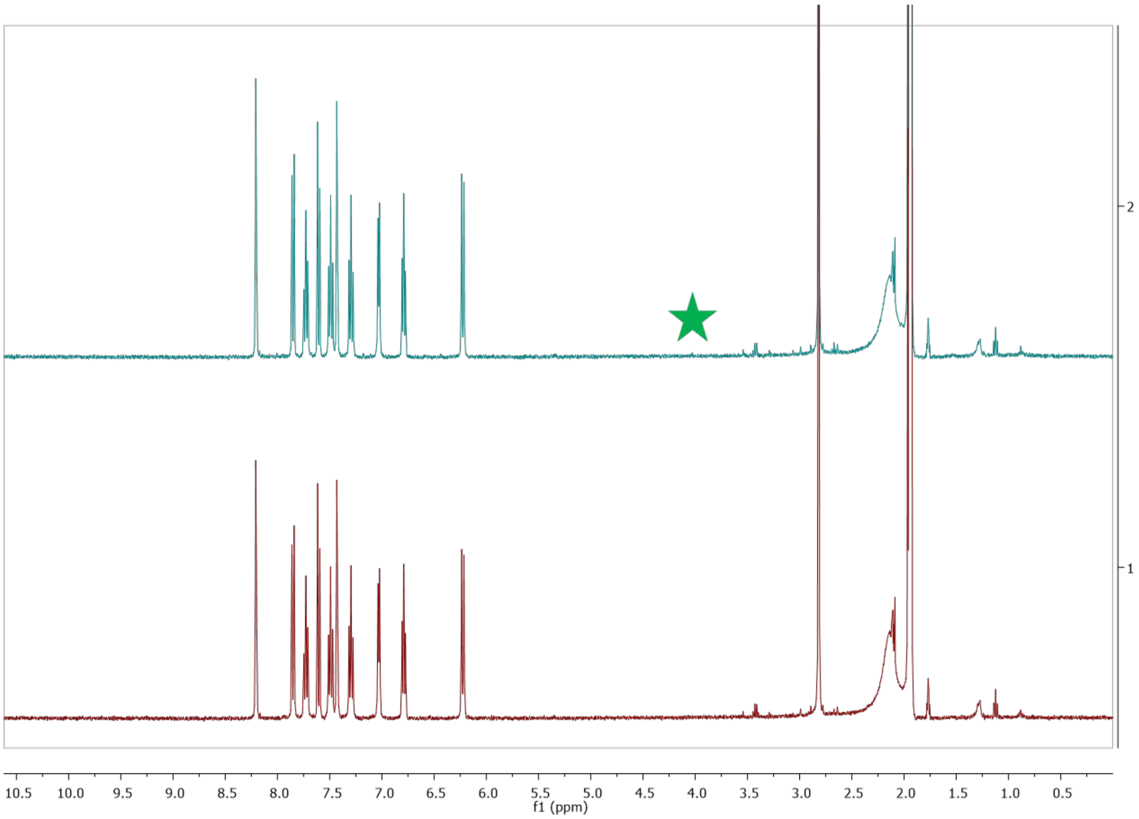

*Figure S 100: ^{1}H-NMR spectrum of **C2** in ACN-d_3 in the dark (inert) (red :0 h; blue 72 h). The signal at 4.03 ppm (marked by green star) indicates the formation of P1*

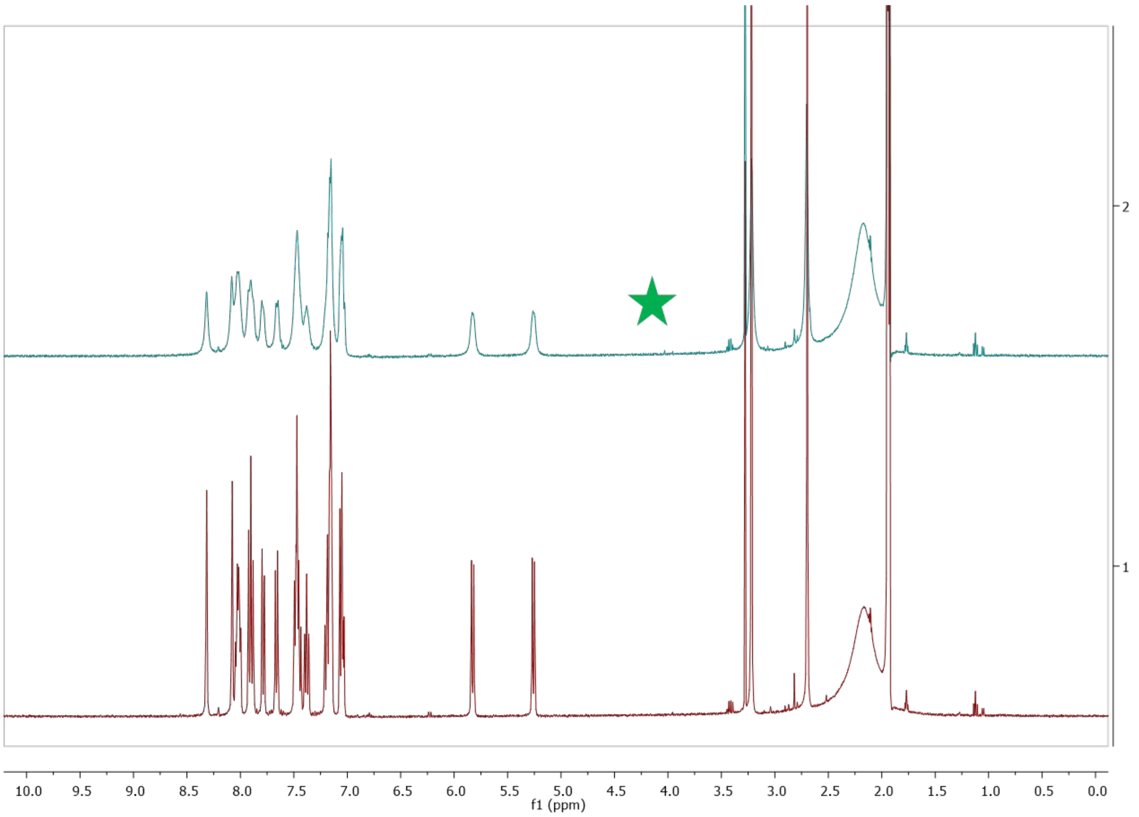

*Figure S 101: ^{1}H-NMR spectrum of **C1** in ACN-d_3 in the dark (air) (red :0 h; blue 72 h). The signal at 4.03 ppm (marked by green star) indicates the formation of P1*

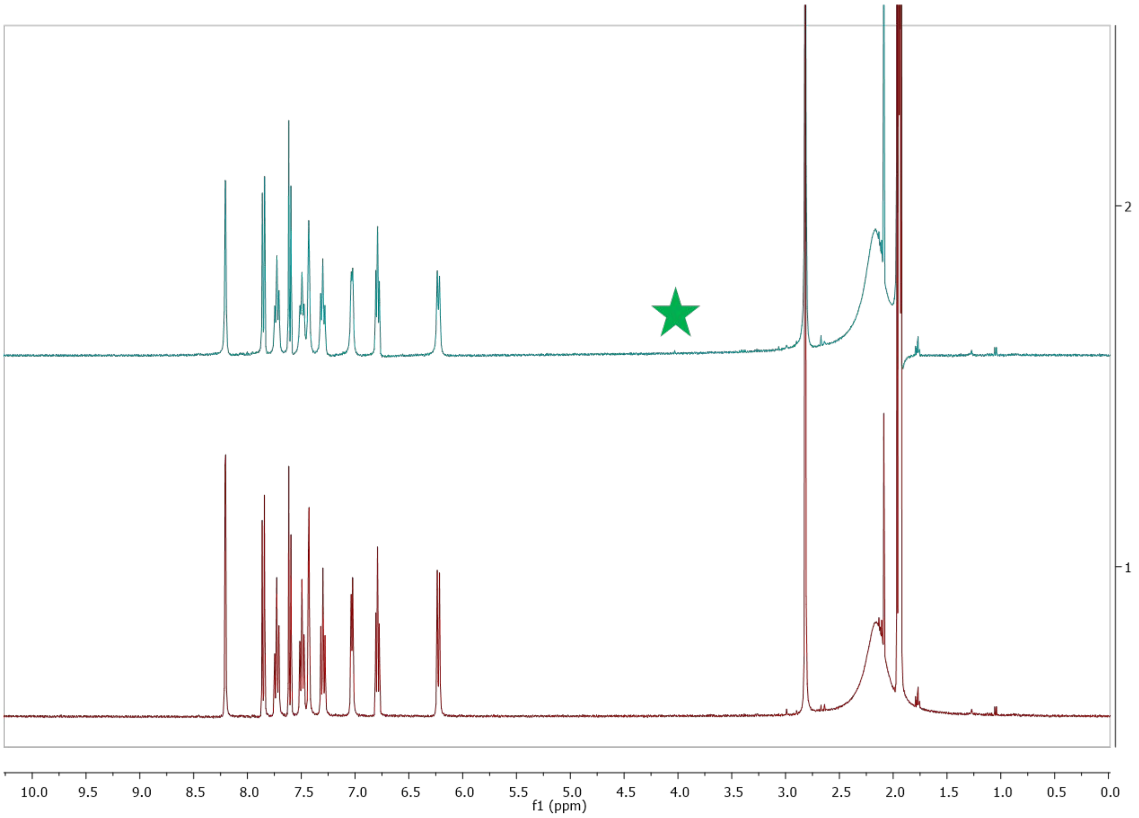

*Figure S 102: ^{1}H-NMR spectrum of **C2** in ACN-d_3 in the dark (air) (red :0 h; blue 72 h). The signal at 4.03 ppm (marked by green star) indicates the formation of P1.*

12.3 Counter ion exchange

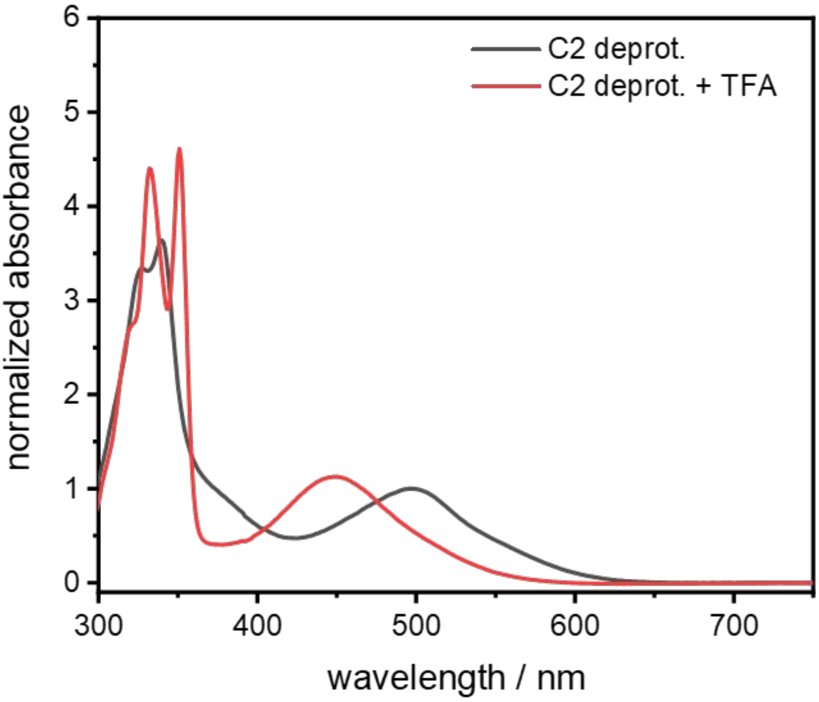

Figure S 103: UV-vis spectra of C2^{deprot} (10^{-5} M) in DMF; before (black) and after (red) addition of trifluoroacetic acid. Spectra are normalized to the MLCT maximum of ***C2***

13. References

[1] T. B. Rauchfuss, Ed. , in *Inorganic Syntheses*, Wiley, **2010**, pp. 129–147.
[2] R. D. Patil, M. Dutta, S. Pratihar, *Organometallics* **2022**, *41*, 2432–2447.
[3] G. M. Sheldrick, *Acta Crystallogr A Found Crystallogr* **2008**, *64*, 112–122.
[4] G. M. Sheldrick, *Acta Crystallogr C Struct Chem* **2015**, *71*, 3–8.
[5] C. F. Macrae, P. R. Edgington, P. McCabe, E. Pidcock, G. P. Shields, R. Taylor, M. Towler, J. Van De Streek, *J Appl Crystallogr* **2006**, *39*, 453–457.
[6] Gaussian 16, Revision C.01, M. J. Frisch, G. W. Trucks, H. B. Schlegel, G. E. Scuseria, M. A. Robb, J. R. Cheeseman, G. Scalmani, V. Barone, G. A. Petersson, H. Nakatsuji, X. Li, M. Caricato, A. V. Marenich, J. Bloino, B. G. Janesko, R. Gomperts, B. Mennucci, H. P. Hratchian, J. V. Ortiz, A. F. Izmaylov, J. L. Sonnenberg, D. Williams-Young, F. Ding, F. Lipparini, F. Egidi, J. Goings, B. Peng, A. Petrone, T. Henderson, D. Ranasinghe, V. G. Zakrzewski, J. Gao, N. Rega, G. Zheng, W. Liang, M. Hada, M. Ehara, K. Toyota, R. Fukuda, J. Hasegawa, M. Ishida, T. Nakajima, Y. Honda, O. Kitao, H. Nakai, T. Vreven, K. Throssell, J. A. Montgomery, Jr., J. E. Peralta, F. Ogliaro, M. J. Bearpark, J. J. Heyd, E. N. Brothers, K. N. Kudin, V. N. Staroverov, T. A. Keith, R. Kobayashi, J. Normand, K. Raghavachari, A. P. Rendell, J. C. Burant, S. S. Iyengar, J. Tomasi, M. Cossi, J. M. Millam, M. Klene, C. Adamo, R. Cammi, J. W. Ochterski, R. L. Martin, K. Morokuma, O. Farkas, J. B. Foresman, and D. J. Fox, Gaussian, Inc., Wallingford CT, **2016**.
[7] A. D. Becke, *The Journal of Chemical Physics* **1993**, *98*, 5648–5652.
[8] C. Lee, W. Yang, R. G. Parr, *Phys. Rev. B* **1988**, *37*, 785–789.
[9] F. Weigend, *Phys. Chem. Chem. Phys.* **2006**, *8*, 1057.
[10] F. Weigend, R. Ahlrichs, *Phys. Chem. Chem. Phys.* **2005**, *7*, 3297.
[11] S. Grimme, S. Ehrlich, L. Goerigk, *J Comput Chem* **2011**, *32*, 1456–1465.
[12] J. J. Sutton, D. Preston, P. Traber, J. Steinmetzer, X. Wu, S. Kayal, X.-Z. Sun, J. D. Crowley, M. W. George, S. Kupfer, K. C. Gordon, *J. Am. Chem. Soc.* **2021**, *143*, 9082–9093.
[13] S. Kupfer, *Phys. Chem. Chem. Phys.* **2016**, *18*, 13357–13367.
[14] C. Latouche, F. Palazzetti, D. Skouteris, V. Barone, *J. Chem. Theory Comput.* **2014**, *10*, 4565–4573.
[15] A. K. Mengele, C. Müller, D. Nauroozi, S. Kupfer, B. Dietzek, S. Rau, *Inorg. Chem.* **2020**, *59*, 12097–12110.
[16] D. Escudero, in *Transition Metals in Coordination Environments* (Eds.: E. Broclawik, T. Borowski, M. Radoń), Springer International Publishing, Cham, **2019**, pp. 259–287.
[17] G. E. Shillito, S. Rau, S. Kupfer, *ChemCatChem* **2023**, *15*, e202201489.
[18] L. González, D. Escudero, L. Serrano-Andrés, *ChemPhysChem* **2012**, *13*, 28–51.
[19] L. Zedler, A. K. Mengele, K. M. Ziems, Y. Zhang, M. Wächtler, S. Gräfe, T. Pascher, S. Rau, S. Kupfer, B. Dietzek, *Angew Chem Int Ed* **2019**, *58*, 13140–13148.
[20] B. Mennucci, C. Cappelli, C. A. Guido, R. Cammi, J. Tomasi, *J. Phys. Chem. A* **2009**, *113*, 3009–3020.
[21] J. Guthmuller, *The Journal of Chemical Physics* **2016**, *144*, 064106.
[22] J. Guthmuller, L. González, *Phys. Chem. Chem. Phys.* **2010**, *12*, 14812.
[23] M. Wächtler, J. Guthmuller, L. González, B. Dietzek, *Coordination Chemistry Reviews* **2012**, *256*, 1479–1508.
[24] R. Siebert, D. Akimov, M. Schmitt, A. Winter, U. S. Schubert, B. Dietzek, J. Popp, *ChemPhysChem* **2009**, *10*, 910–919.
[25] J. Kübel, R. Schroot, M. Wächtler, U. S. Schubert, B. Dietzek, M. Jäger, *J. Phys. Chem. C* **2015**, *119*, 4742–4751.
[26] B. Dietzek, T. Pascher, V. Sundström, A. Yartsev, *Laser Phys. Lett.* **2007**, *4*, 38–43.
[27] C. Müller, T. Pascher, A. Eriksson, P. Chabera, J. Uhlig, *J. Phys. Chem. A* **2022**, *126*, 4087–4099.
[28] K. Kunnus, M. Vacher, T. C. B. Harlang, K. S. Kjær, K. Haldrup, E. Biasin, T. B. Van Driel, M. Pápai, P. Chabera, Y. Liu, H. Tatsuno, C. Timm, E. Källman, M. Delcey, R. W. Hartsock, M. E. Reinhard, S. Koroidov, M. G. Laursen, F. B. Hansen, P. Vester, M. Christensen, L. Sandberg, Z. Németh, D. S. Szemes, É. Bajnóczi, R. Alonso-Mori, J. M. Glownia, S. Nelson, M. Sikorski, D. Sokaras, H. T. Lemke, S. E. Canton, K. B. Møller, M. M. Nielsen, G. Vankó, K. Wärnmark, V. Sundström, P. Persson, M. Lundberg, J. Uhlig, K. J. Gaffney, *Nat Commun* **2020**, *11*, 634.
[29] S. Gründemann, A. Kovacevic, M. Albrecht, J. W. Faller, R. H. Crabtree, *J. Am. Chem. Soc.* **2002**, *124*, 10473–10481.
